# Global burden of primary liver cancer by five etiologies and global prediction by 2035 based on global burden of disease study 2019

**DOI:** 10.1002/cam4.4551

**Published:** 2022-02-04

**Authors:** Yuan Liu, Jinxin Zheng, Jialing Hao, Rang Rang Wang, Xueni Liu, Peng Gu, Hongwei Yu, Yang Yu, Chuanxing Wu, Baochi Ou, Zhihai Peng

**Affiliations:** ^1^ Shanghai General Hospital Shanghai Jiao Tong University Shanghai China; ^2^ Department of Nephrology, Ruijin Hospital, Institute of Nephrology Shanghai Jiao Tong University School of Medicine Shanghai China; ^3^ The First Affiliated Hospital of Anhui Medical University Hefei China; ^4^ Department of General Surgery, Xiang'an Hospital of Xiamen University, School of Medicine Xiamen University Xiamen Fujian China; ^5^ Organ Transplantation Institute of Xiamen University, Fujian Provincial Key Laboratory of Organ and Tissue Regeneration, School of Medicine Xiamen University Xiamen China

**Keywords:** alcoholic use, HBV, HCV, liver cancer, NASH

## Abstract

**Background:**

Using data from the global burden of disease (GBD) between 1990 and 2019 to report the leading etiological factors and hazards for liver cancer by HBV (LCHB), HCV (LCHC), alcoholic use (LCAL), NASH (LCNA), and other causes (LCOT).

**Method:**

The estimated annual percentage change (EAPC) and age‐standardized incidence rate (ASR) in different districts, sex, and age are used to quantify the change of etiologies of liver cancer. Age‐period‐cohort models were performed to predict the primary liver cancer incidence and case numbers.

**Results:**

Based on the GBD database of the whole world for the five etiologies of liver cancer in 2019, the percentage of incidence of LCAL, LCHB, LCHC, LCNA, and LCOT are 18.4%, 41%, 28.5%, 6.8%, and 5.3%, respectively. Fiver etiologies of liver cancer show gender differences, with LCHB and LCAL being more prevalent in men, and LCHC, LCNA being more prevalent in women. Besides, live cancer of males is because of alcohol using and smoking, while the reason of liver cancer of females is drug use, high BMI and high fasting plasma glucose. Interestingly, the incidence of LCHC in women over 85 years old, LCNA in women over 75 years old, and LCOT in women over 75 years old were all higher than that in men. According to the future prediction, the incidence rate of liver cancer itself, as well as the five causes of liver cancer, tends to decrease gradually after 2019, while the incidence rate of LCNA in males will continue to increase until 2025.

**Conclusions:**

The incidence of liver cancer has been increasing and its major causes vary considerably at global, regional, or national levels, also vary by gender and age group.

## Graphical abstract

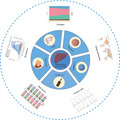

## 1 INTRODUCTION

Primary liver cancer, consisting of hepatocellular carcinoma, cholangiocarcinoma, and mixed carcinoma, is the sixth most commonly diagnosed cancer and the fourth leading cause of cancer death globally in 2018.^1^ On a global scale, East Asia has the highest number of people with liver cancer and Australia has the highest growth rate.^2^ In contrast, Central Europe and East Asia have a growth rate of incidence below 0 and the incidence of liver cancer in 2019 has declined by an estimated 10% compared to 1990. In addition to this, the age‐standardized incidence rates and estimated annual percentage change have declined, but the challenge on liver cancer treatment remains hard and huge work remains to be done to tackle the problem.

While previous studies have elaborated on the four major etiologies of liver cancer, including hepatitis B (LCHB), hepatitis C(LCHC), liver cancer attributed to alcohol use (LCAL), and other causes (LCOT) of liver cancer, in the last few years, liver cancer due to nonalcoholic steatohepatitis (NASH) has gradually gained attention as the fifth leading cause of liver cancer.^3^, ^4^, ^5^, ^6^, ^7^, ^8^ From a global perspective, the etiology of liver cancer can differ drastically from one region to another. For example, in East Asia, infection with hepatitis B and aflatoxin are the predominant causative factors, however, the burden of hepatitis B in Southern Latin America is the lowest.^9^ In Japan and Egypt, hepatitis C serves as the principal culprit and as to liver cancer due to alcoholism, the highest age‐standardized incidence, and the age‐standardized death rate is in Southeast Asia. Since the pathogenesis of liver cancer fluctuates from region to region, the prevention of its occurrence can be more efficiently based on the characteristics of each regional area.^9^

We used the GBD database to collect data on the etiology of liver cancer in 204 countries and regions over the three decades from 1990 to 2019, to explore the patterns and trends in liver cancer incidence. In a previous study, Liu et al. analyzed the incidence of liver cancer in each geographical region worldwide between 1990 and 2017,^10^ while there is a huge difference in 2019 data, showing the emergence of 2019 data analysis. In the present study, we analyzed the five causes of liver cancer, LCHB, LCHC, LCAL, and LCNA, as well as LCOT, at the global, regional, and national levels, and in addition, we focused on the differences in incidence between gender and age group, and explore the risk factors each liver cancers. Besides, we predicted the incidence of primary liver cancer in 2035. Our research can enhance and broaden previous studies and assist in the prevention of primary liver cancer in different regions and countries in new data 2019.

## 2 METHODS

### 2.1 Study data

Global Burden of Disease Study 2019 consisting of 204 countries and territories, estimates incidence, death, and others due to 369 diseases and injuries with two sexes, among which we observe the incidence of liver cancer. The estimation of incidence was based on DisMod‐MR 2.1, a Bayesian meta‐regression, ensuring consistency.^11^The database was led by the Institute for Health Metrics and Evaluation (IHME) and the GBD study is the most comprehensive worldwide observational epidemiological study to date. The data downloaded in http://ghdx.healthdata.org/. In this study, we observed the incidence of five etiologies of primary liver cancer, including hepatitis B, hepatitis C, alcohol consumption, NASH, and other causes, as well as total primary liver cancer. Other causes are not a major etiology of liver cancer and a collection of causes, including possibly aflatoxin, which is an important factor affecting liver cancer.^5^ We also observed different etiologies in an aspect of sociodemographic index (SDI) level, including low, low‐middle, middle, high‐middle, and high.

### 2.2 GBD risk factor hierarchy

To accommodate diverse interests, the GBD database has a risk factor hierarchy. Level 1 risk factors are behavioral, environmental, occupational, and metabolic.^12^ In this study, we chose behavioral and metabolic factors to explore the etiology of liver cancer. Behavioral factors include smoking, alcohol use, drug use, and so on. Metabolic factors include high fasting plasma glucose, high LDL cholesterol, high systolic blood pressure, and so on.

### 2.3 Ethics statement

This research was approved by the Ethics Committee of Shanghai General Hospital. The methods were carried out following the Declaration of Helsinki and its later amendments or comparable ethical standards.

### 2.4 Statistical analysis

We used the age‐standardized incidence rate (ASR) and estimated annual percentage change (EAPC) to quantify the trends of liver cancer from 1990 to 2019. The ASR is calculated according to a weighted mean of the age‐specific rates and the weights are taken from the population distribution of the standard population.^13^ The ASR is calculated by summing up the products of the age‐specific rates (ai) and the amounts of persons (Wi) in the same age subgroup, then dividing the sum of standard population weights. The former is as follows:

$$\mathrm{ASR}=\frac{\sum_{i=1}^{A}\text{aiWi}}{\sum_{i=1}^{A}\mathrm{Wi}}\times 100,000$$

The UIs (uncertainty intervals) were calculated from 1000 times in the ensemble and meta‐regression model, and produced 95% UIs which were defined by the 2.5th and 97.5th percentiles of the 1000 estimates draws.^14^ The EAPC is used to describe the magnitude of change in the trend on fitting a simple regression model and the formula written as y = α + βx + ɛ, in which y = ln(ASR), and x = calendar year, further its 95% confidence interval (CI) was also obtained from this linear regression model. Age‐period‐cohort models were performed to predict the primary liver cancer incidence and case numbers through 2035.^15^ The prediction was conducted in R software through the NORDPRED package, which has been recognized to perform well in projecting current trends in cancer incidence into the future. The number of new cases were predicted for the year 2035 by taking a weighted average of the projected incidence rates for the last two prediction periods, centering on 2035, and then applying the rates to the UN national population forecasts available for each country for that year. All statistical analysis was performed in R software.

## 3 RESULTS

### 3.1 Global liver cancer burden

**Figure 1:**
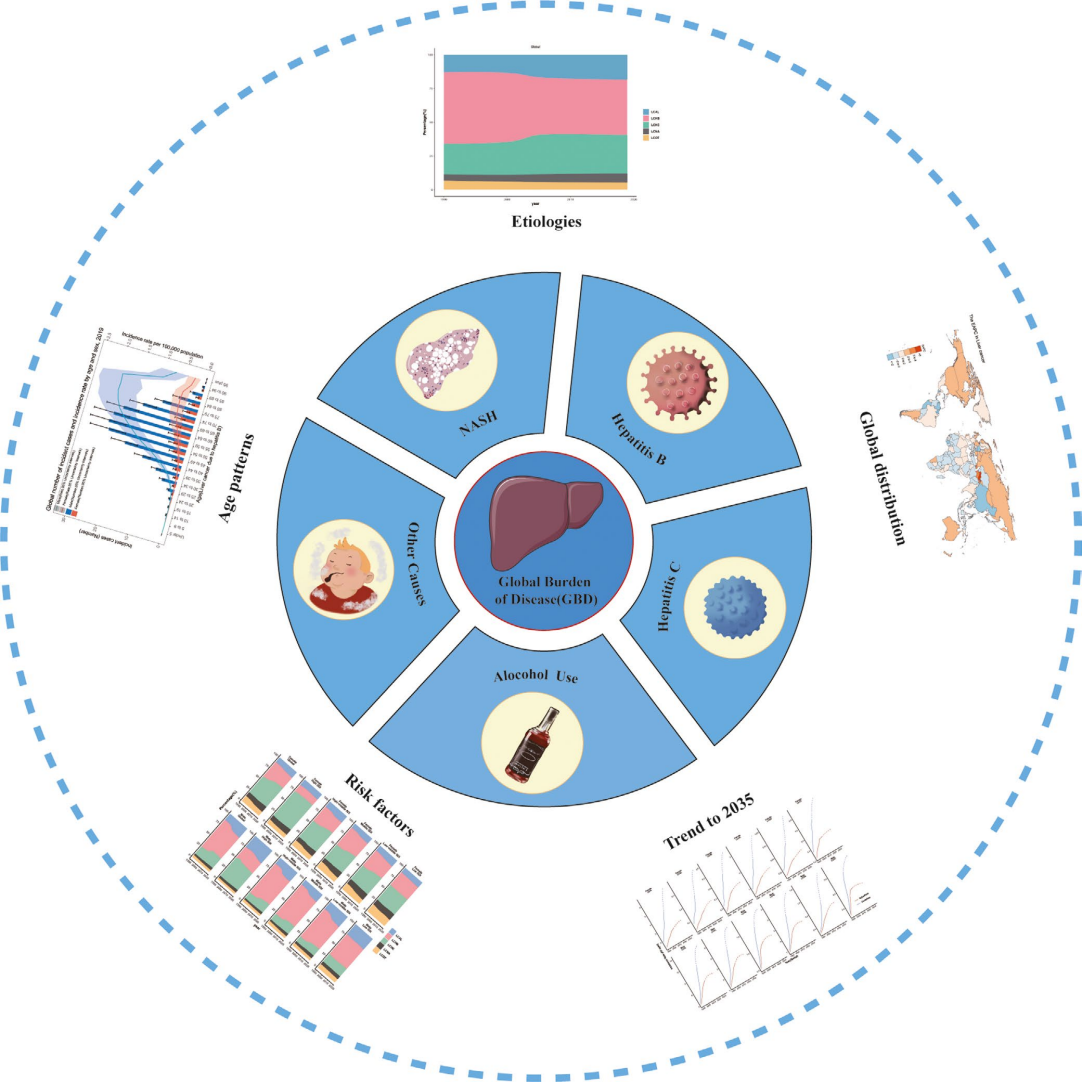
Landscape of the study. This study demonstrated the etiologies of liver cancer by HBV (LCHB), HCV (LCHC), alcoholic use (LCAL), NASH (LCNA), and other causes (LCOT). We used gender and age to quantify changes in the etiology of liver cancer and forecast the incidence of liver cancer in 2035

**Figure 2:**
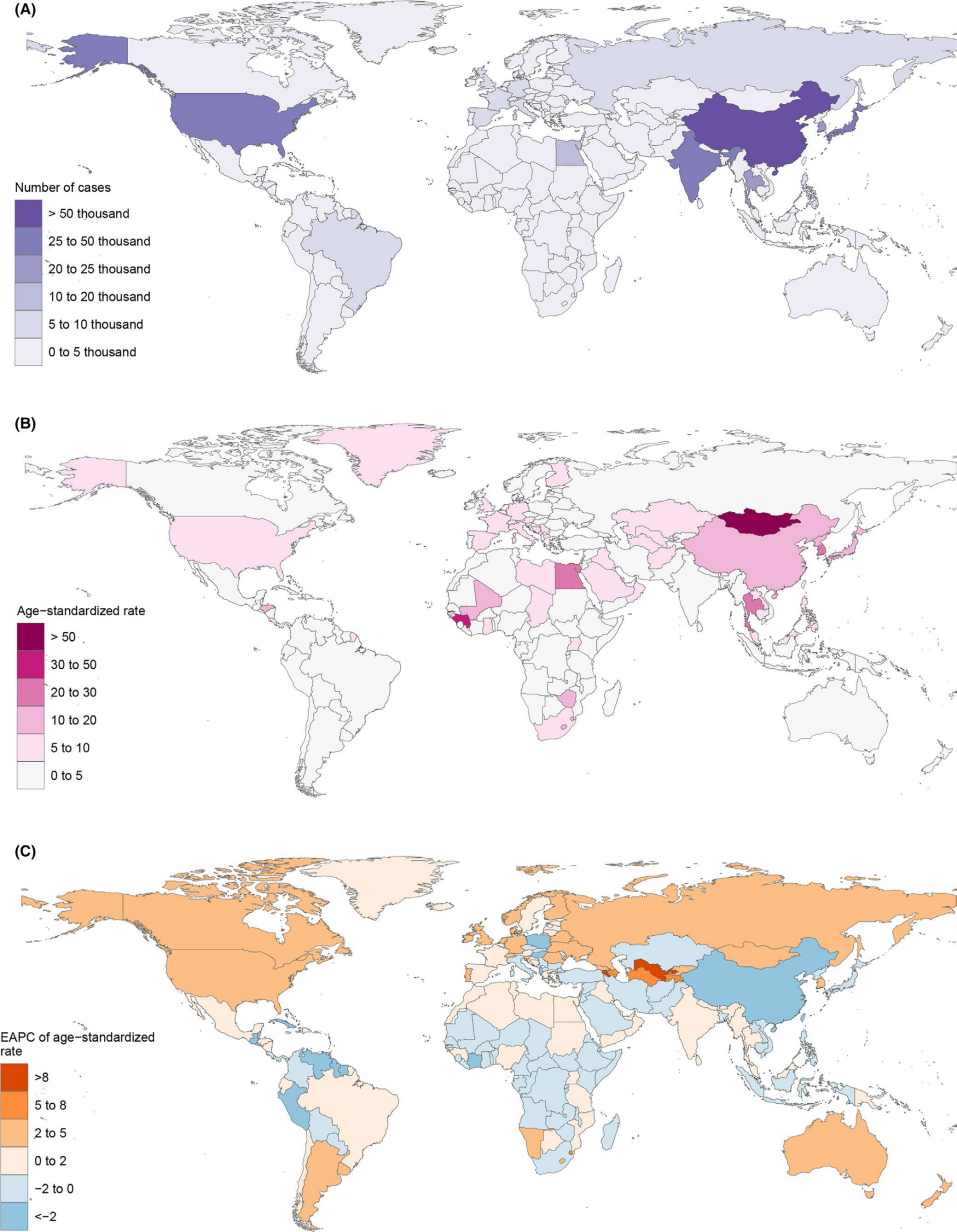
Global liver cancer burden. (A)The number of cases of global liver cancer; (B) age‐standardized rate of global liver cancer; (C) EPAC of the age‐standardized rate of global liver cancer

Figure 1 shows the landscape of the study. We analyzed the five leading etiologies of liver cancer, broken down by SDI, age, or sex. Besides, we also did a trend prediction of liver cancer for 2035. Globally, Liver cancer's incidence increased by 43.11% from 1990 to 2019. The most significant increase for ASR was detected in Australasia (Figure 2C, EAPC = 3.139, 95% CI: 2.87–3.407), followed by High‐income North America and Central Asia. Liver cancer in 2019 reported in GBD database, there are 218,000 HBV, 152,000 HCV, 36,000 NASH, 98,000 alcohol using, and 28,000 other incident cases (Table 1). As shown in Figure 2A, the topmost newly diagnosed liver cancer cases were reported in China in 2019 (210,462.35, 95%CI, 174,831.65‐251,195.06), followed by Japan and India. Liver cancer's ASR varies widely around the world and the maximum ASR is in Mongolia (Figure 2B, 105.22 per 100,000), followed by The Gambia (38.21 per 100,000) in 2019. The details of ASR, EAPC, and incidence for five etiologies of liver cancer are shown in Figure S1. It is worth mentioning that there is a big difference in amounts of primary liver cancer in 2019 compared to before, thus we explored the reason and showed the difference in regional and national levels between 2019 and 2017 in Figure S1.

**Table 1:** Global burden of primary liver cancer by gender, etiology, and region from 1990 to 2019

Characteristic	1990		2019		1990–2019
	Incident cases, no. ×10^3^ (95% UI)	ASR per 100000, no. (95% UI)	Incident cases, no. × 10^3^ (95% UI)	ASR per 100000, no. (95% UI)	EAPCs, no. (95% CI)
Global	373 (336 to 416)	8.98 (8.10 to 9.97)	534 (487 to 589)	6.51 (5.95 to 7.16)	−1.93 (−2.30 to −1.55)
Sociodemographic index					
High SDI	53 (52 to 55)	5.27 (5.11 to 5.39)	140 (126 to 154)	7.61 (6.88 to 8.36)	0.91 (0.47 to 1.35)
High‐middle SDI	109 (95 to 123)	9.92 (8.73 to 11.20)	107 (94 to 121)	5.34 (4.70 to 6.04)	−3.3 (−3.89 to −2.77)
Middle SDI	166 (144 to 194)	14.73 (12.80 to 17.04)	186 (162 to 211)	7.32 (6.41 to 8.28)	−2.86 (−3.41 to −2.31)
Low‐middle SDI	35 (31 to 39)	5.36 (4.85 to 5.93)	55 (50 to 62)	3.99 (3.60 to 4.44)	−1.62 (−1.94 to −1.30)
Low SDI	10 (9 to 12)	4.08 (3.58 to 4.63)	19 (17 to 22)	3.51 (3.11 to 3.91)	−0.28 (−0.36 to −0.21)
Gender					
Male	260 (227 to 298)	13.07 (11.42 to 14.87)	376 (335 to 422)	9.71 (8.69 to 10.84)	−1.92 (−2.33 to −1.51)
Female	113 (99 to 129)	5.22 (4.62 to 5.96)	158 (140 to 176)	3.63 (3.23 to 4.05)	−1.87 (−2.16 to 1.59)
Etiology					
HBV	198 (166 to 232)	4.60 (3.86 to 5.39)	219 (186 to 255)	2.62 (2.24 to 3.05)	−3.10 (−3.66 to −2.53
HCV	84 (73 to 96)	2.19 (1.90 to 2.49)	152 (132 to 175)	1.90 (1.64 to 2.17)	−1.04 (−1.27 to −0.81)
Alcohol	48 (38 to 59)	1.20 (0.96 to 1.47)	98 (79 to 120)	1.19 (0.96 to 1.45)	−0.40 (−0.57 to −0.22)
NASH	18 (15 to 21)	0.44 (0.36 to 0.53)	36 (29 to 45)	0.45 (0.37 to 0.55)	−0.51 (−0.79 to −0.24)
Others	25 (21 to 30)	0.56 (0.47 to 0.67)	28 (24 to 34)	0.35 (0.29 to 0.42)	−2.42 (−2.86 to −1.99)
Region					
North Africa and Middle East	11 (9 to 12)	6.08 (5.28 to 6.81)	28 (22 to 34)	6.29 (5.13 to 7.71)	0.49 (0.32 to 0.67)
High‐income Asia Pacific	28 (27 to 29)	13.77 (13.3 to 14.19)	68 (58 to 78)	15.56 (13.46 to 17.74)	−0.17 (−08 to 0.39)
Tropical Latin America	2 (2 to 2)	1.97 (1.88 to 2.04)	6 (5 to 6)	2.36 (2.22 to 2.48)	1.05 (0.89 to 1.21)
Eastern Sub‐Saharan Africa	2 (2 to 3)	2.93 (2.44 to 3.7)	5 (4 to 7)	3.13 (2.6 to 3.81)	−0.03 (−0.15 to 0.1)
Australasia	0.5 (0.5 to 0.5)	2.05 (1.97 to 2.13)	2 (2 to 3)	4.59 (3.72 to 5.68)	3.14 (2.87 to 3.41)
Western Sub‐Saharan Africa	5 (4 to 6)	5.45 (4.60 to 6.32)	10 (8 to 11)	4.93 (4.19 to 5.72)	−0.47 (−0.54 to −0.39)
Andean Latin America	1 (0.9 to 1)	4.90 (4.31 to 5.52)	2 (1 to 2)	3.11 (2.55 to 3.78)	−2.01 (−2.45 to −1.57)
Central Europe	8 (7 to 8)	5.25 (5.06 to 5.39)	7 (6 to 8)	3.29 (2.85 to 3.81)	−1.32 (−1.69 to −0.96)
Southern Latin America	0.7 (0.6 to 0.8)	1.56 (1.41 to 1.71)	2 (2 to 2)	2.33 (1.83 to 2.92)	2.05 (1.81 to 2.30)
South Asia	16 (13 to 18)	2.66 (2.2 to 3.08)	38 (33 to 43)	2.66 (2.30 to 3.05)	−0.02 (−0.10 to 0.06)
East Asia	241 (204 to 85)	25.26 (21.46 to 29.74)	217 (181 to 257)	10.43 (8.76 to 12.30)	−4.60 (−5.39 to −3.80)
Southeast Asia	17 (15 to 19)	6.43 (5.69 to 7.06)	43 (35 to 52)	7.07 (5.87 to 8.61)	0.31 (0.25 to 0.37)
Central Latin America	3 (3 to 3)	3.49 (3.24 to 3.68)	8 (7 to 9)	3.43 (2.96 to 3.97)	0.11 (−0.22 to 0.43)
High‐income North America	8 (7 to 8)	2.20 (2.14 to 2.25)	31 (26 to 37)	5.18 (4.28 to 6.18)	2.99 (2.79 to 3.20)
Caribbean	2 (1 to 2)	5.94 (5.52 to 6.30)	2 (1 to 2)	3.16 (2.63 to 3.77)	−2.09 (−2.89 to −1.28)
Southern Sub‐Saharan Africa	2 (1 to 3)	6.47 (4.52 to 10.72)	4 (4 to 5)	6.77 (6.07 to 7.61)	−0.43 (−1.00 to 0.14)
Oceania	0.1 (0.01 to 0.1)	3.65 (3.08 to 4.25)	0.2 (0.1 to 0.3)	3.28 (2.77 to 3.89)	−0.22 (−0.28 to −0.16)
Eastern Europe	4 (4 to 4)	1.52 (1.45 to 1.59)	9 (8 to 11)	2.84 (2.46 to 3.24)	2.52 (2.27 to 2.78)
Central Sub‐Saharan Africa	0.7 (0.6 to 0.8)	2.63 (2.20 to 3.08)	1 (1 to 2)	2.30 (1.84 to 2.86)	−0.61 (−0.68 to −0.539)
Central Asia	1 (1 to 2)	3.13 (2.79 to 3.47)	6 (5 to 7)	8.27 (7.22 to 9.41)	2.78 (2.24 to 3.32)
Western Europe	20 (19 to 21)	3.55 (3.43 to 3.64)	46 (40 to 53)	5.31 (4.59 to 6.12)	1.37 (1.19 to 1.55)

Abbreviations: ASR, age‐standardized incidence rate; EPAC, the estimated annual percentage change.

### 3.2 Liver cancer caused by five different etiologies

**Figure 3:**
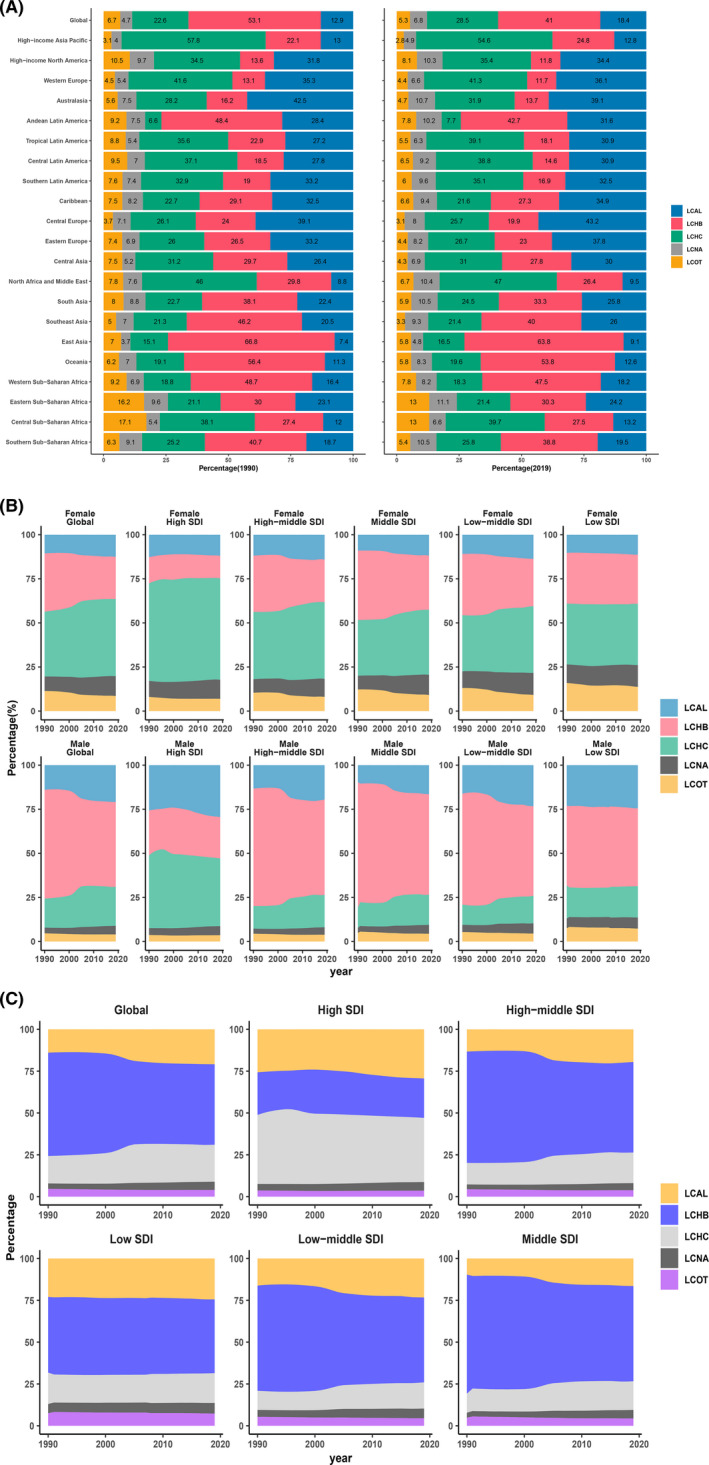
Liver cancer caused by five different etiologies**.** (A) Percentages of liver cancer caused by five etiologies worldwide in 1990 and 2019; (B) percentages of liver cancer caused by five etiologies in males and females from 1990 to 2019; (C) percentages of liver cancer caused by five etiologies globally and across different SDI regions

The newest GBD data released in 2019, the percentage of LCAL, LCHB, LCHC, LCNA, and LCOT are 18.4%, 41%, 28.5%, 6.8%, and5.3%, respectively. While the percentage of LCAL, LCHB, LCHC, LCNA, and LCOT are 12.9%, 53.1%, 22.6%, 4.7%, and 6.7%, respectively in 1990 (Figure 3A). As shown in Figure 3B, the percentage of LCAL and LCHB in men was greater than that in women, while the percentage of LCHC and LCNA in women was higher than that in men in 2019. Figure 3C shows that the percentage of LCHB is gradually decreasing, while LCAL and LCHC are gradually increasing globally, while in low‐SDI and high‐SDI regions, the percentage of LCHB and LCHC almost keeps stable. Interestingly, the proportion of risk factors for liver cancer in males is mostly because of alcohol using and smoking, while in females is drug use, high BMI, and high fasting plasma glucose (Figure S2). We speculated that the reason might be that drug use can destroy the immune microenvironment of the liver and accelerate the occurrence of liver cancer. In addition, drug use may cause damage to the liver, which is prone to becoming cancerous as it repairs the damage.

### 3.3 Trends of ASR in different SDI between male and female

**Figure 4:**
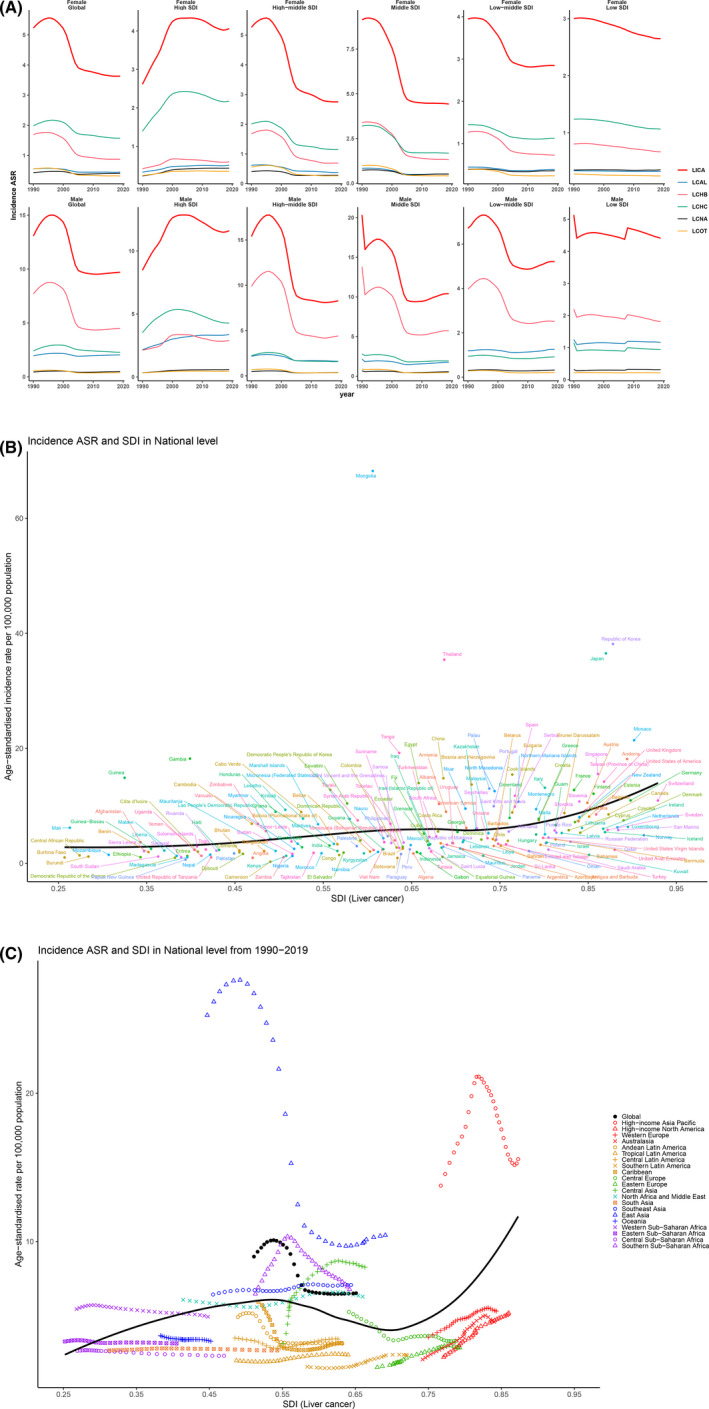
Trends of ASR in SDI between males and females. (A) The trend of liver cancer's ASR in males and females globally and across different SDI regions; (B) global distribution of SDI (liver cancer) and age‐standardized incidence rate in different countries; (C) global distribution of SDI (liver cancer) and age‐standardized incidence rate from 1990 to 2019 for different regions

In terms of ASR, from 1990 to 1996, the ASR rose at a steady speed in males, females, or both. From then until 2004, there was a pronounced decline in ASR. Between 2004 and 2019, the trend of liver cancer's ASR was relatively smooth (Figure 4A). However, the global ASR of liver cancer in females was on a downward curve. The ASR of LCHB and LCAL is much lower in females than in men, with a large disparity between both. On the contrary, the ASR of LCHC is lower in men than in women. Figure 4B demonstrated that the Mongolia has the highest ASR, followed by the Republic of Korea and Thailand in 2019. It is obvious that ASR gradually increased with the rise of SDI in liver cancer. In terms of change of ASR from 1990 to 2019, the more prominent region was East Asia, where the SDI peaked at roughly 0.47, followed by a rapid decline and a gradual leveling off of ASR (Figure 4C. In the high‐income Asia Pacific region, ASR increases rapidly with SDI and then decreases sharply. Among LCHB, the change is most pronounced in East Asia, with the ASR peaking when the SDI reaches about 0.5 (Figure S3).

### 3.4 Incidence rate by age and sex

**Figure 5:**
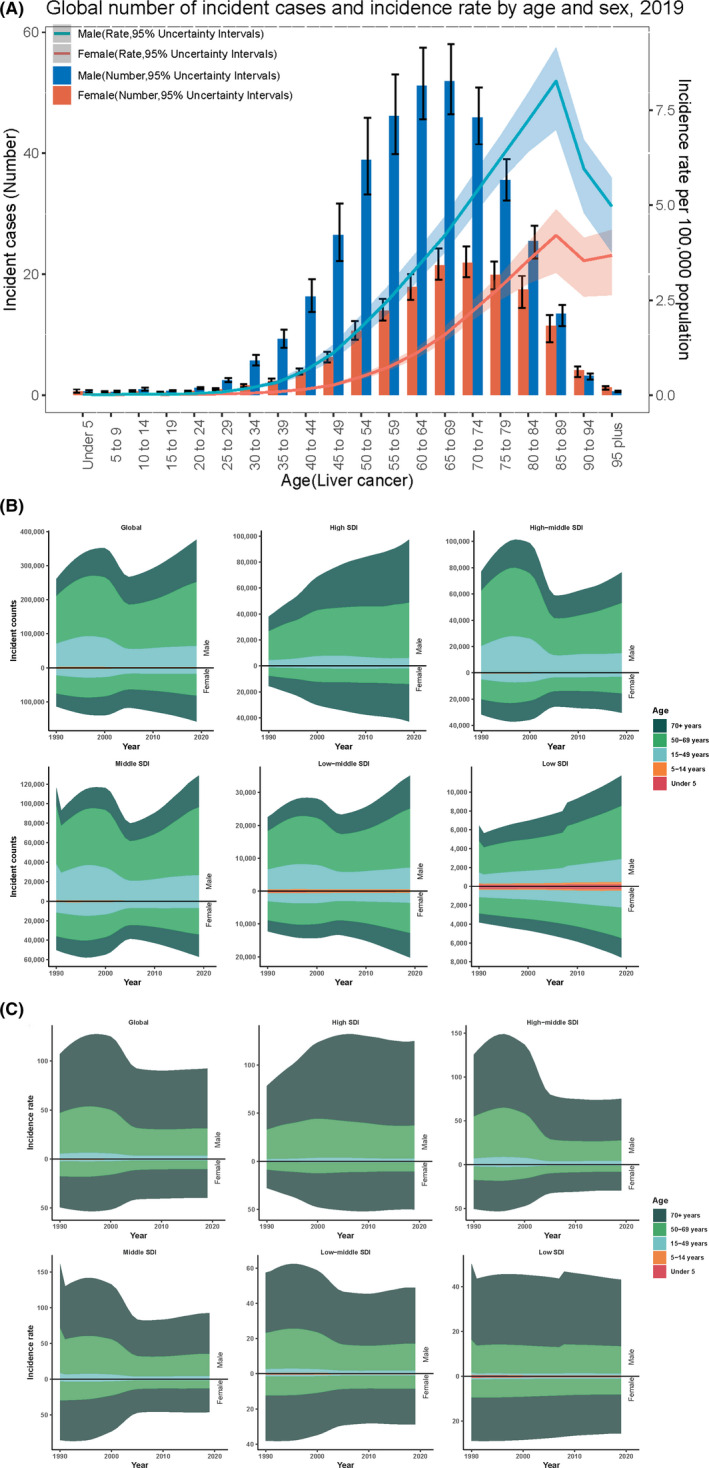
The correlation of age and incident cases of liver cancer by sex and SDI. (A) The global number of incident cases and incidence rate by age and sex of liver cancer; (B) distribution of the incident counts in different age groups from 1990 to 2019 globally and in different SDI regions; (C) distribution of the incident rates in different age groups from 1990 to 2019 globally and in different SDI regions

The global number of incident cases and incidence rate by age and sex in 2019 are shown in Figure 5. Geographically, the incidence rate goes up for both gender as we age to 89 years, but is always higher in men than in women and the rate begins to decline after age 89. As shown in Figure 5B,C, liver cancer is more likely to occur at an age of 50–69 years in men, while in the female the incidence of liver cancer is similar between the age of 50–69 years and 70+ years. No matter male and female, the increased rate of liver cancer at an age of 70+ years is higher than 50–69 years. It is to mention that the incidence of liver cancer in SDI district at an age less than 14 years is more distinct than others. Figure S4 showed that the incidence of LCHC in women over 85 years old, LCNA in women over 75 years old, and LCOT in women over 75 years old were all higher than that in men.

### 3.5 Prediction of the incidence rate of liver cancer in 2035

**Figure 6:**
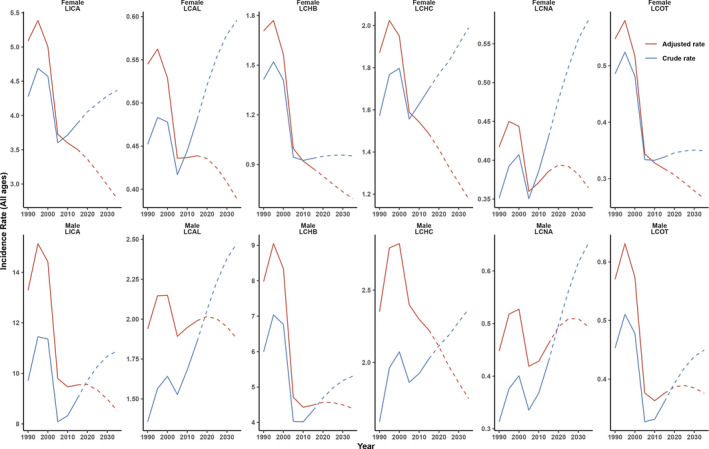
Prediction of the incidence rate (all ages) of primary liver cancer by sex in 2035. LICA: Liver cancer; LCAL: Liver cancer by alcoholic use; LCHB: Liver cancer by HBV; LCHC: Liver cancer by HCV; LCNA: Liver cancer by NASH; LCOT: Liver cancer by other causes

The incidence rate of liver cancer is shown in crude rate and adjusted rate, respectively. There are two clear turning points for LCAL, LCHB, LCHC, LCNA, LCOT, as well as for liver cancer (LICA), around 1996 and 2010 (Figure 6). The incidence rate of men is higher than that in females from 1990 to 2035. However, after adjusting the rate, the incidence rate is gradually declining to 2035 both in men and female for liver cancer. Interestingly, the incidence rate of LCNA in man will continue to increase until 2025 and then turn down. We also show the prediction of incidence rate to 2035 in China and the USA respectively in Figure S5.

## 4 DISCUSSION

Drawing on the GBD database of 204 countries, 21 regions for the five etiologies of liver cancer and the gender as well as age differences between men and women in 2019, the following conclusions were achieved: First, the percentage of LCAL, LCHB, LCHC, LCNA, and LCOT is 18.4%, 41%, 28.5%, 6.8%, and 5.3%, respectively in 2019. Second, the main causes of liver cancer show gender differences, with LCHB and LCAL being more prevalent in men, and LCHC, LCNA being more prevalent in women. Third, the proportion of liver cancer in males is mostly because of alcohol using and smoking, while in females is drug use, high BMI, and high fasting plasma glucose. Four, liver cancer is more likely to occur at an age of 50–69 years in men. Besides, the incidence of LCHC in women over 85 years old, LCNA in women over 75 years old, and LCOT in women over 75 years old were all higher than those in men. Fifth, according to the predicted model, the incidence rate of liver cancer itself, as well as the five causes of the disease, tends to decrease gradually after 2019, while the incidence rate of LCNA in men will continue to increase until 2025.

Of all the causes, LCHB and LCHC continue to dominate liver cancer, which is largely consistent with what has been said before.^5^ For over three decades, LCHB has been the leading etiology of liver cancer, but notably, its share has been declining. East and Southeast Asian countries have always had a high incidence of LCHB, for which China, Korea, India, Japan, and Thailand have made great efforts to reduce the incidence.^16^ China is the largest country with LCHB and the government has reduced the incidence of hepatitis B through vaccination, interruption of mother‐to‐child transmission and prevention of blood product transmission, but there is still a long way to go before the goals of reducing mortality and increasing diagnosis and treatment are met.^17^, ^18^, ^19^ Notably, the incidence of LCHB has declined in most SDI countries, except for countries with high‐SDI countries, which increased at a low steady rate.

Unlike hepatitis B, there is no specific vaccine for hepatitis C, which makes it more challenging to manage. However, benefiting from early screening for HCV and the advent of Direct‐Acting Antiviral, the ASR for LCHC has shown a declining pattern.^4^, ^20^, ^21^ The cause of Hepatitis C differs between countries. In developing countries, it is mainly due to infections of medical origin, contaminated needles, etc., while in developed countries it is mainly due to exposure to high‐risk factors and injection drug use, unclean sexual practices, etc^22^, ^23^ The exact association between HCV and liver cancer is unclear. The reasons might be that HCV in the liver causes a high turnover of liver cells by destruction and repairment. Besides, HCV might interfere with the mechanism that repairs damage to DNA within cells or destroys a key protective tumor‐suppressing gene.

The prevalence of LCHC in China has remained the highest, followed by Japan and the United States of America. Thanks to proactive efforts in a wide range of countries and regions, liver cancer caused by HBV has declined significantly, but the incidence rate of liver cancers caused by alcohol use and HCV are gradually increasing. In the US, nonalcoholic fatty liver disease and alcoholic liver disease are the most prevalent causes for liver transplant patients, even surpassing hepatitis C. One of the major reasons for this is the shift in lifestyle in modern times, the uncontrolled consumption of alcohol, premature exposure to alcohol, and poor social alcohol culture. Consequently, valid alcohol policies are of critical importance, such as raising taxes on alcoholic beverages, elevating the legal age at which alcohol can be purchased, and prohibiting alcohol as a gift or in advertisements.^24^, ^25^

NASH is a newly classified major cause of liver cancer in recent years and becomes more and more important. With the prevalence of obesity and metabolic syndrome, etc., the liver cancer resulting from NASH is on the rise year by year and should be given adequate attention,^8^ which is consistent with our study. Of the many countries and regions, India has the most significant case increase of NASH‐related liver cancer, followed by the United States of America and Thailand, while Poland has the greatest decline of LCNA. This phenomenon indicates that India has high levels of obesity and metabolic syndrome.^26^, ^27^, ^28^ Therefore, lifestyle interventions and dietary changes are crucial to reducing the incidence of LCNA.^6^, ^7^

As mentioned earlier, differences exist in the causes of liver cancer between men and women.^29^, ^30^ In the case of alcohol, for example, the incidence ASR of LCAL is higher in men than in women. Presumably because in contrast to women, men drink significantly more alcohol than women and interestingly, women are more sensitive to alcohol‐related damage than men.^31^, ^32^ Differences were also evident in HCV‐induced liver cancer, with a significantly higher proportion in women than men, which is consistent with studies showing that women over 60 have more HCV.^33^ In addition to this, HBV‐related liver cancer is more prominently found in postmenopausal females than in premenopausal women.^34^ Sex hormones could be the explanation for this phenomenon.^35^ In the case of liver cancer, estrogen has a protective effect on women and is associated with the immune response to HBV infection.^36^ In addition, the prevalence of LCNA is greater in women than in men. This is consistent with the results that NASH prevalence was substantially higher in women than men.^37^

For different age groups, there are also significant disparities in the incidence of liver cancer over the three decades. Incidence has risen among those older than 50 years in males, mainly due to an aging population and obesity. Conversely, it declined dramatically among those younger than 49 years of age in high‐SDI countries, primarily owing to the gains in HBV control.^10^

As can be seen, the global incidence rate of primary liver cancer is expected to slow down around 2027, thanks to the control of HBV, HCV, the main cause of liver cancer, and the availability of new treatment modalities. The WHO Hepatitis Eradication project and the public health policies of different countries have played a huge role in controlling hepatitis. At the same time, the incidence of NASH, alcoholic liver cancer, etc. is increasing year by year, which is enough to draw our attention. Active measures should be taken to intervene.

The strength of this study is that it explores the five etiological changes in liver cancer and the differences between men and women and different age groups based on GBD data over the three decades from 1990 to 2019. Projections of future liver cancer incidence were also made. However, some limitations still need to be noted: First, in some countries with low‐ and middle‐income levels, the constraints of medical care may lead to incomplete diagnostic data and biased results. Second, this study only covered the effects of individual etiologies and did not involve the combined effects of multiple etiologies, which should be taken into consideration in the following studies.

In summary, the prevention and treatment of liver cancer have progressed over the past three decades and the effects of previous vaccines are now beginning to be seen. Prevention and active treatment of HBV, HCV, etc. at the source have also been undertaken by various countries and the international community. However, with economic and social development and lifestyle changes, the number of liver cancers caused by new risk factors is increasing and should be taken seriously. This research provides insights into the causes of liver cancer and the differences between the sexes and age groups, which will help to better understand the current state of liver cancer prevention and treatment worldwide and to design more targeted measures.

## CONFLICT OF INTEREST

The authors of this manuscript have no conflict of interest to disclose.

## AUTHOR CONTRIBUTIONS

Conceptualization: Zhihai Peng and Yuan Liu, Methodology: Jinxin Zheng, Yuan Liu, Yang Yu, and Chuanxing Wu, Investigation: Rangrang Wang, Xueni Liu, and Peng Gu, Visualization: Jinxin Zheng and Yuan Liu, Funding acquisition: Zhihai Peng, Writing – original draft: Yuan Liu, Jialing Hao, and Hongwei Yu, Writing – review & editing: Yuan Liu, Jinxin Zheng, and Jialiang Hao.

## Supporting information


Figure S1

Figure S2

Figure S3

Figure S4

Figure S5

## Data Availability

The data described in this article are openly available in the GBD database (http://ghdx.healthdata.org/gbd‐results‐tool).
