# Supplementary figures and images for: Global burden of primary liver cancer by five etiologies and global prediction by 2035 based on global burden of disease study 2019
Source: Cancer Med. 2022 Feb 4;11(5):1310–23. doi: 10.1002/cam4.4551 (PMC8894698; doi:10.1002/cam4.4551)

A

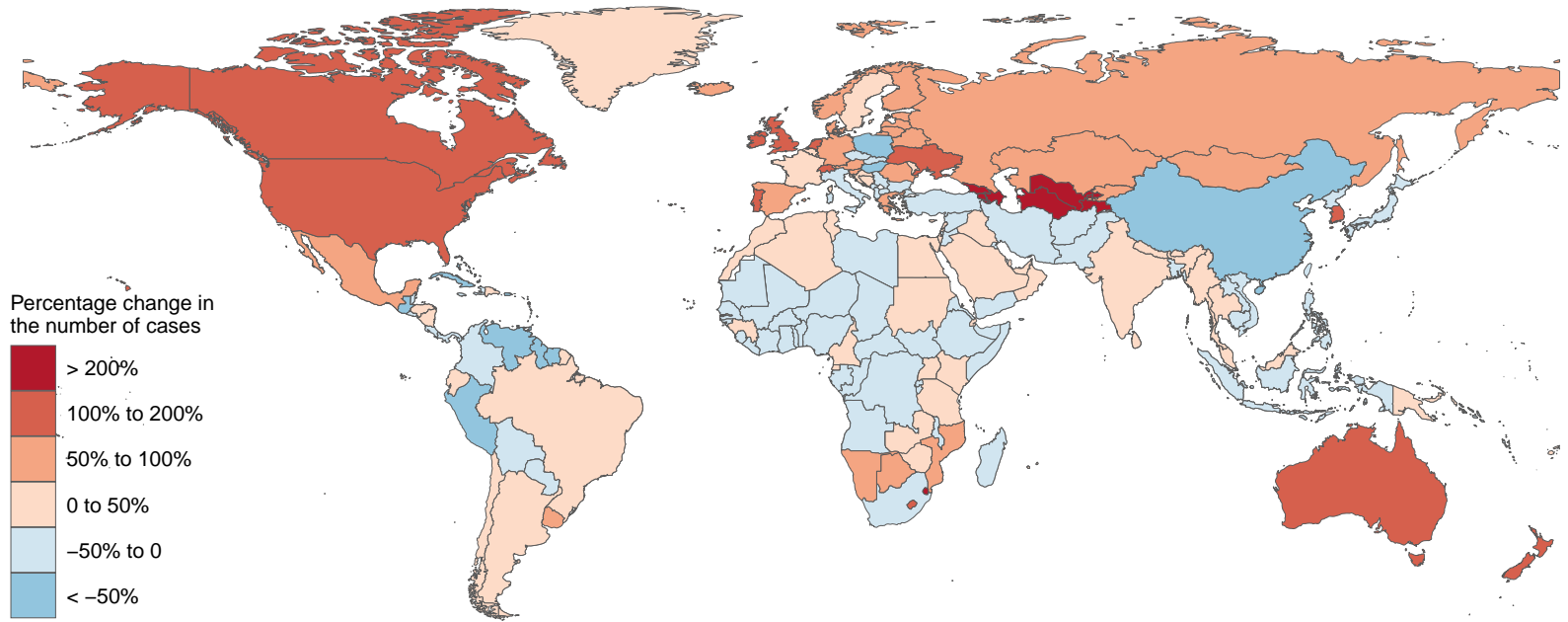

B

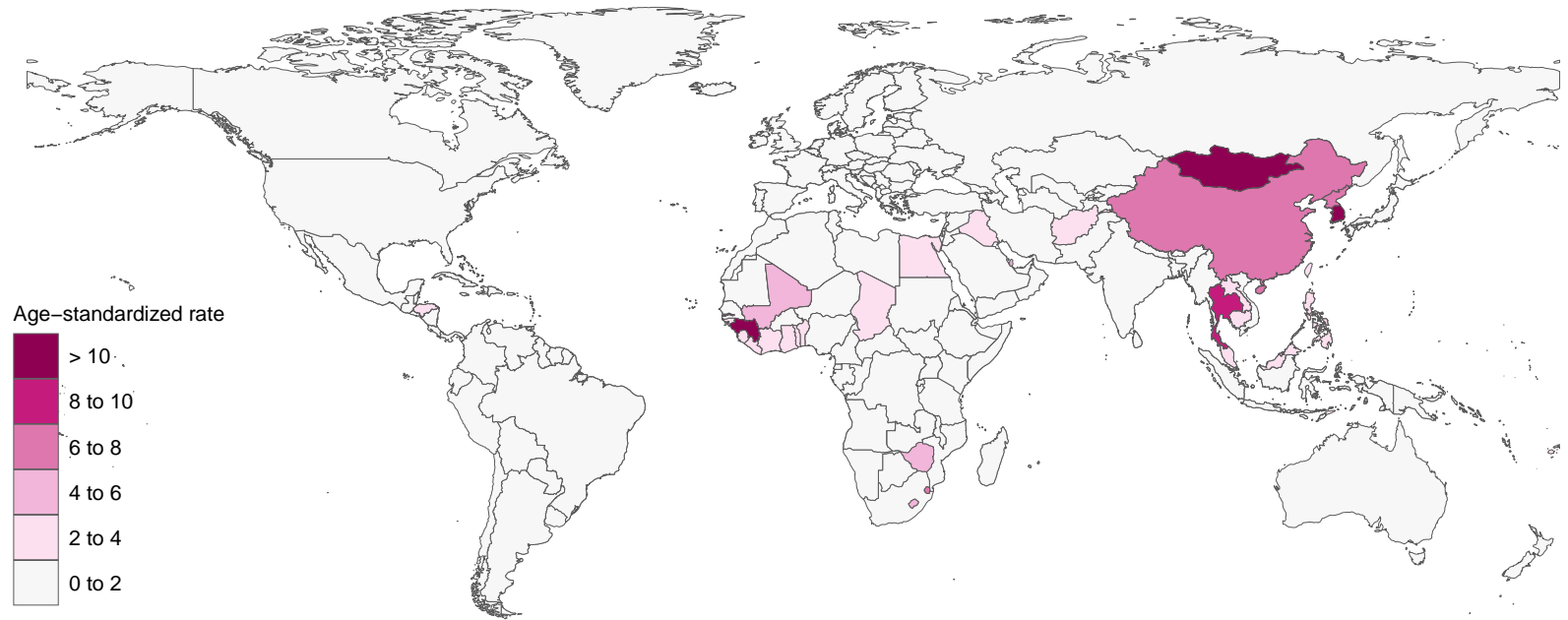

C

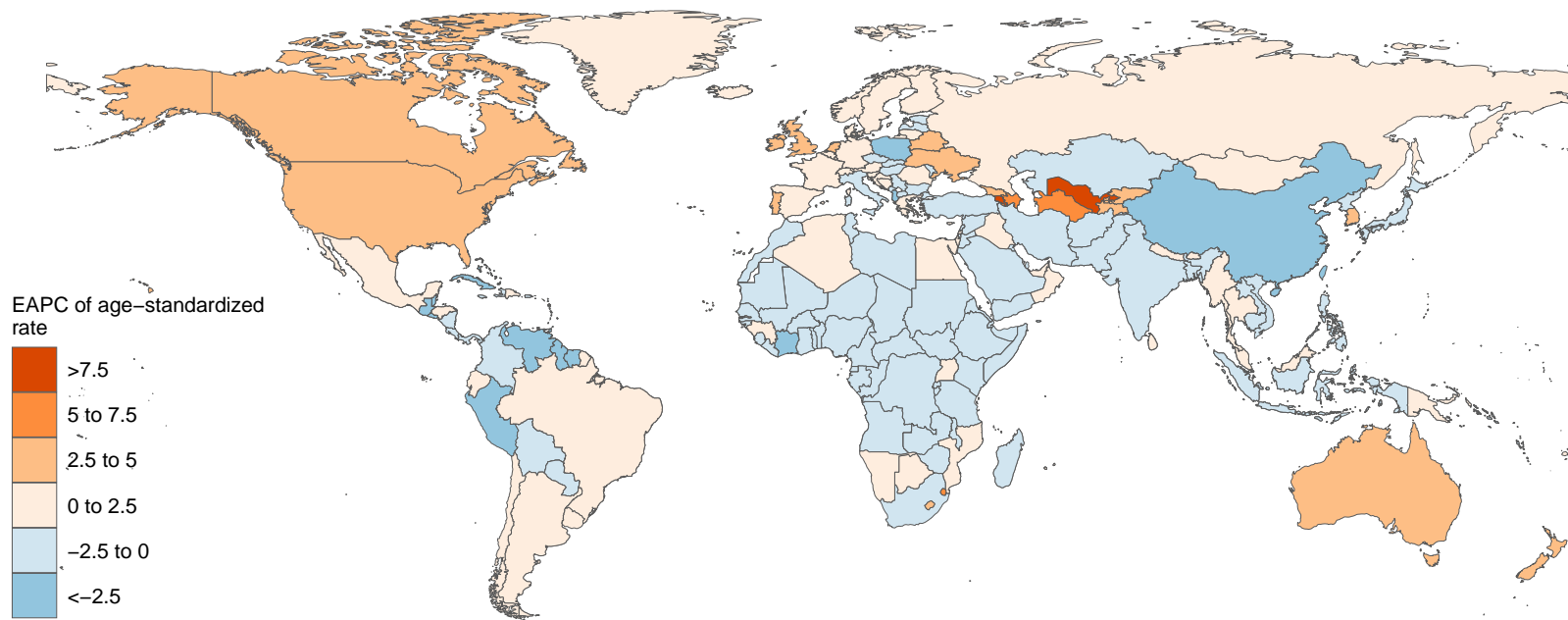

D

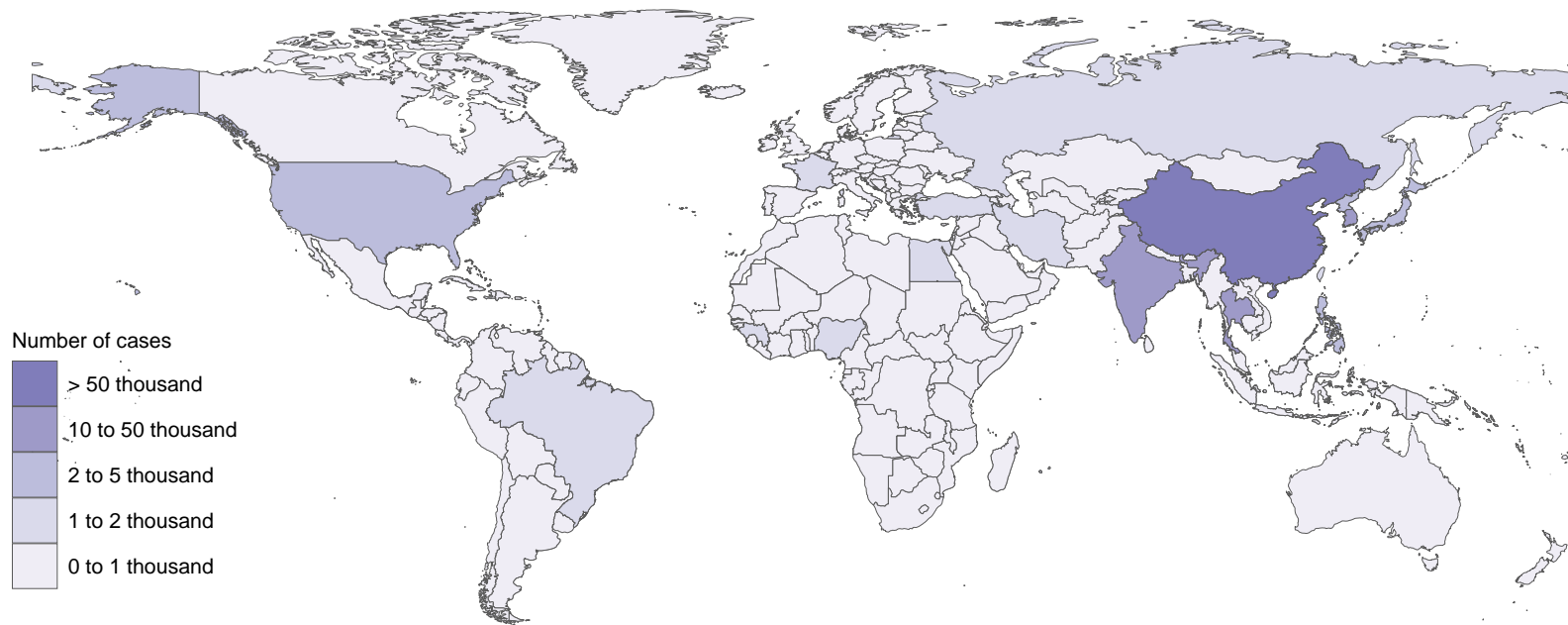

E

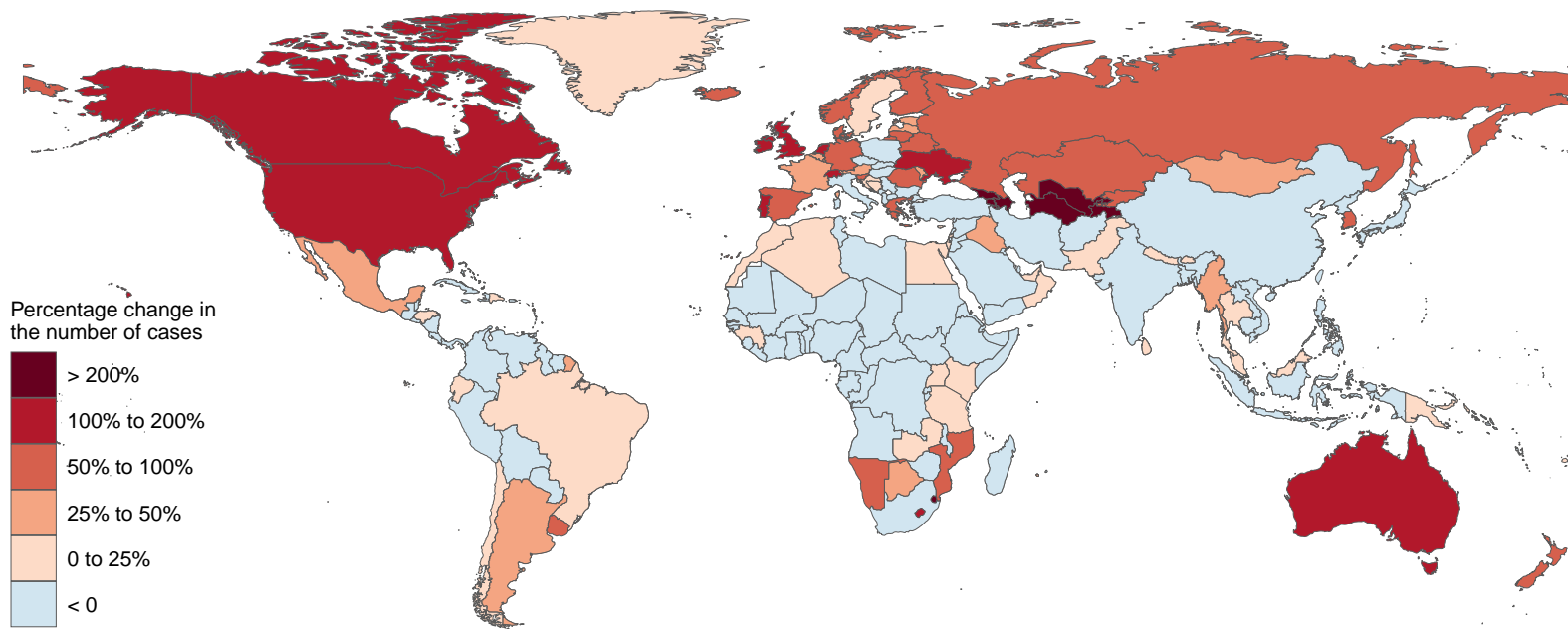

F

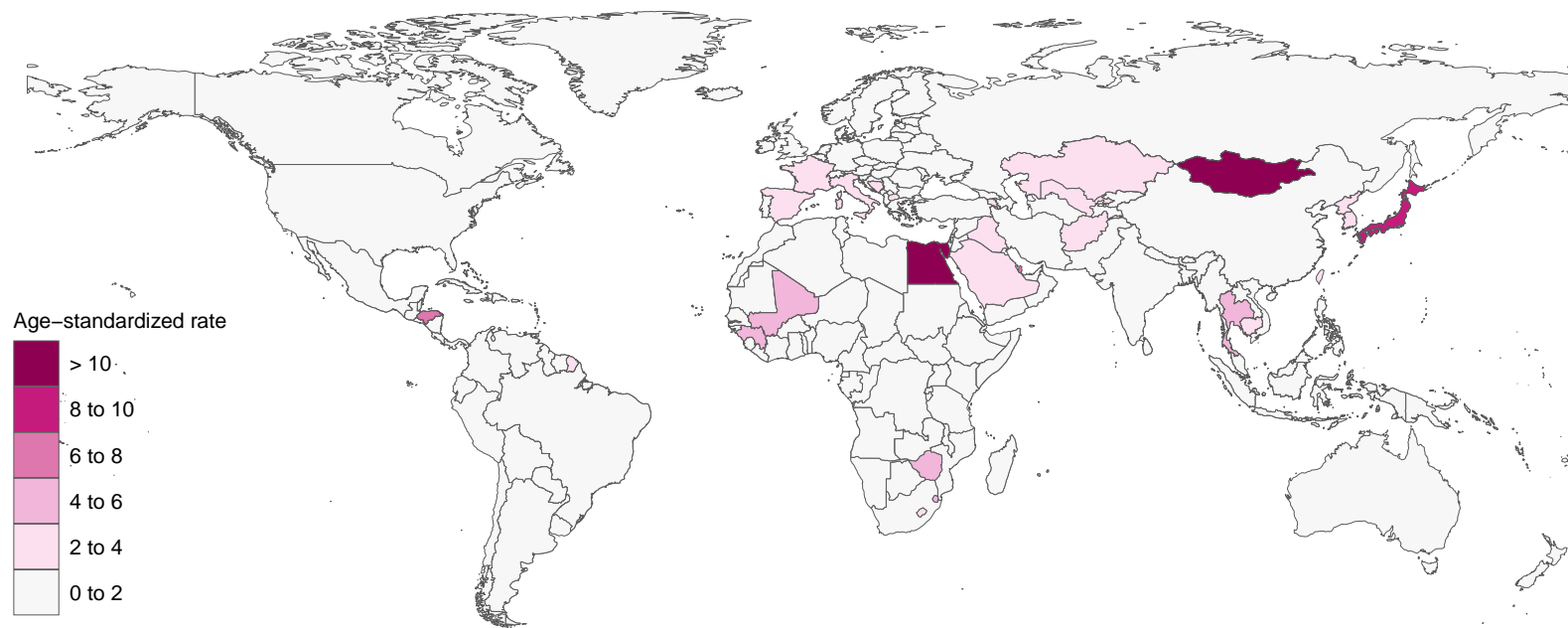

G

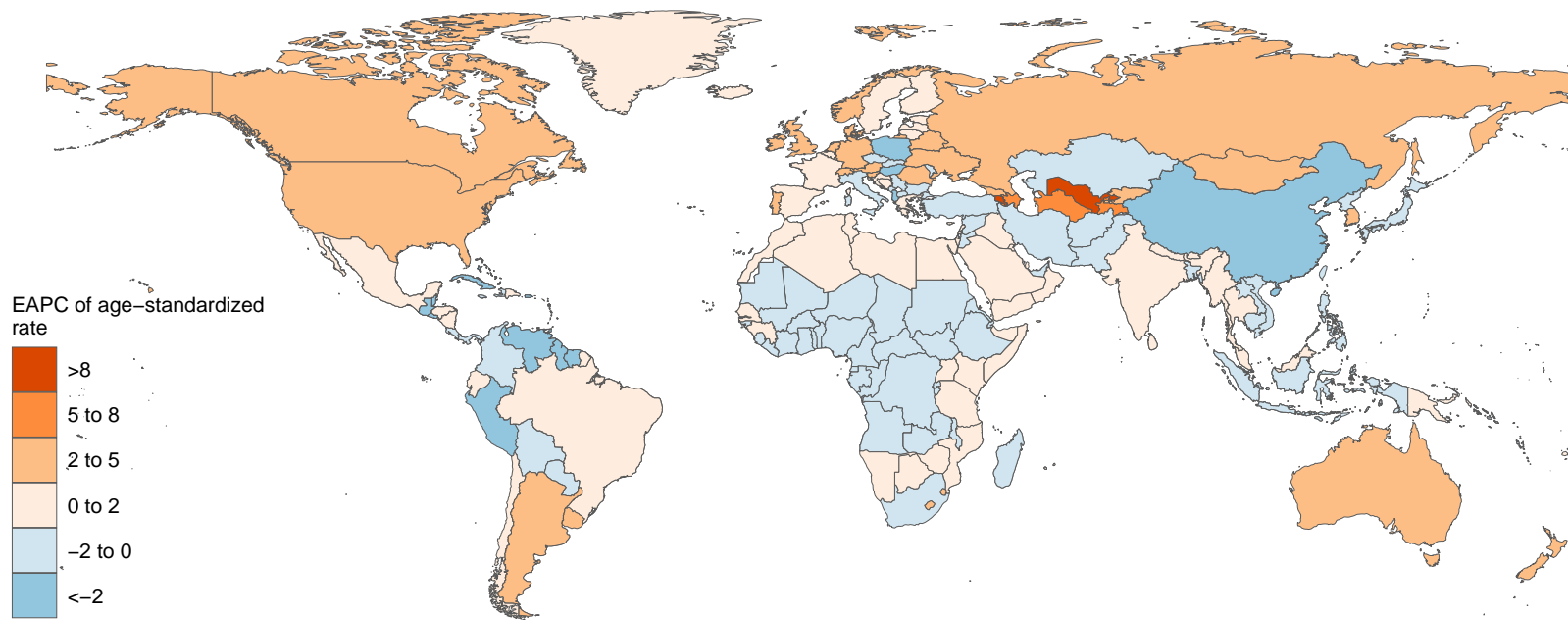

H

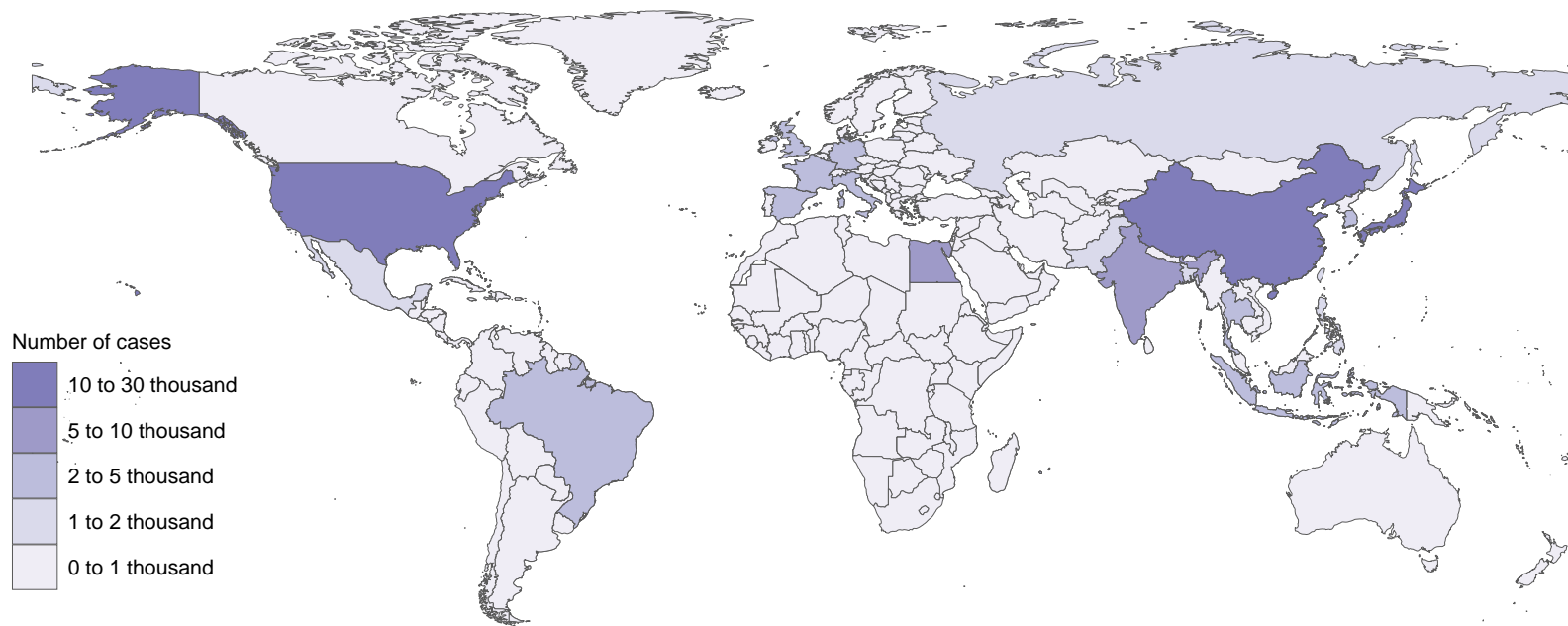

I

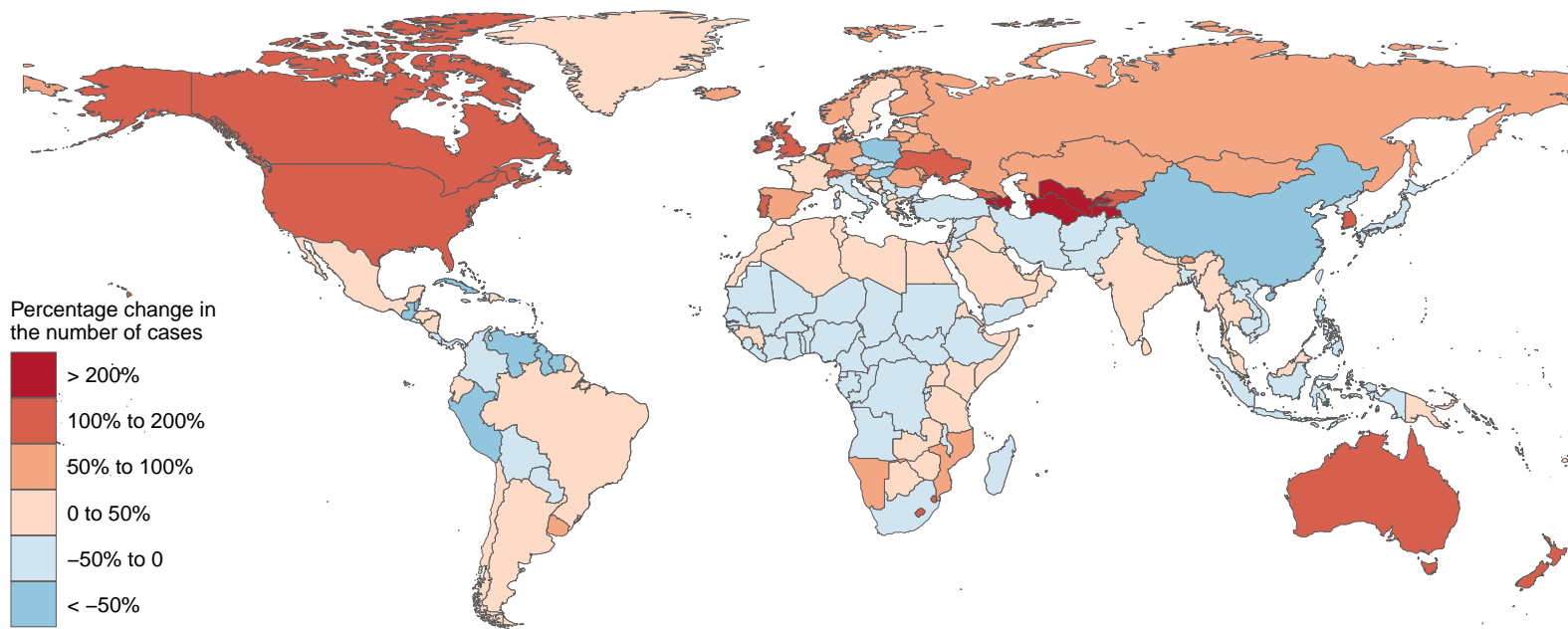

J

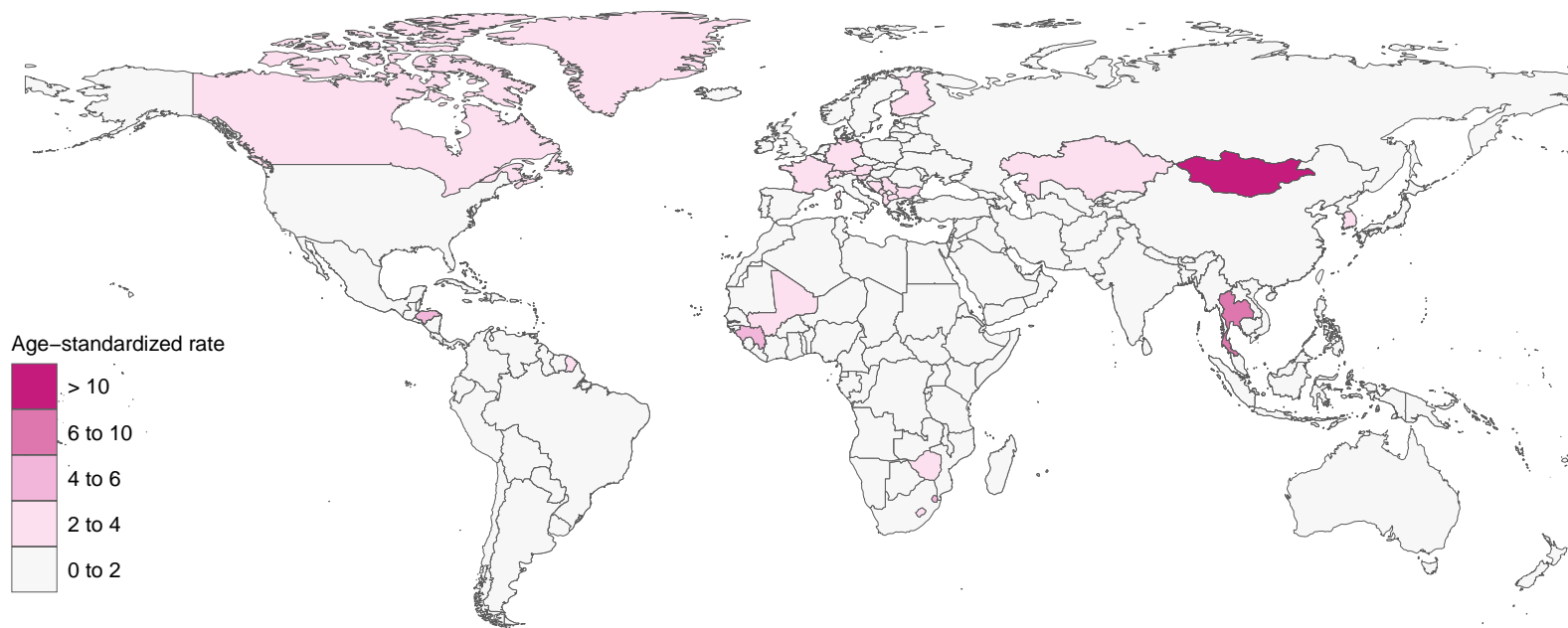

K

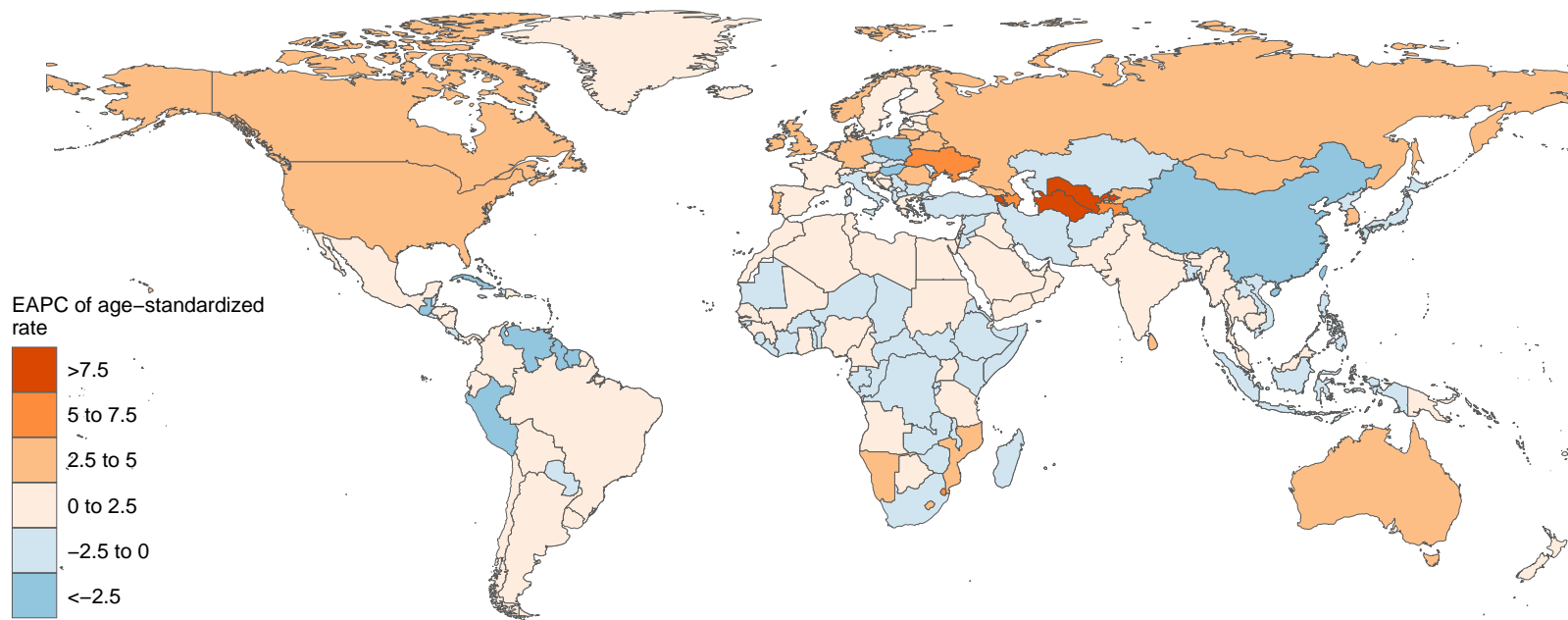

L

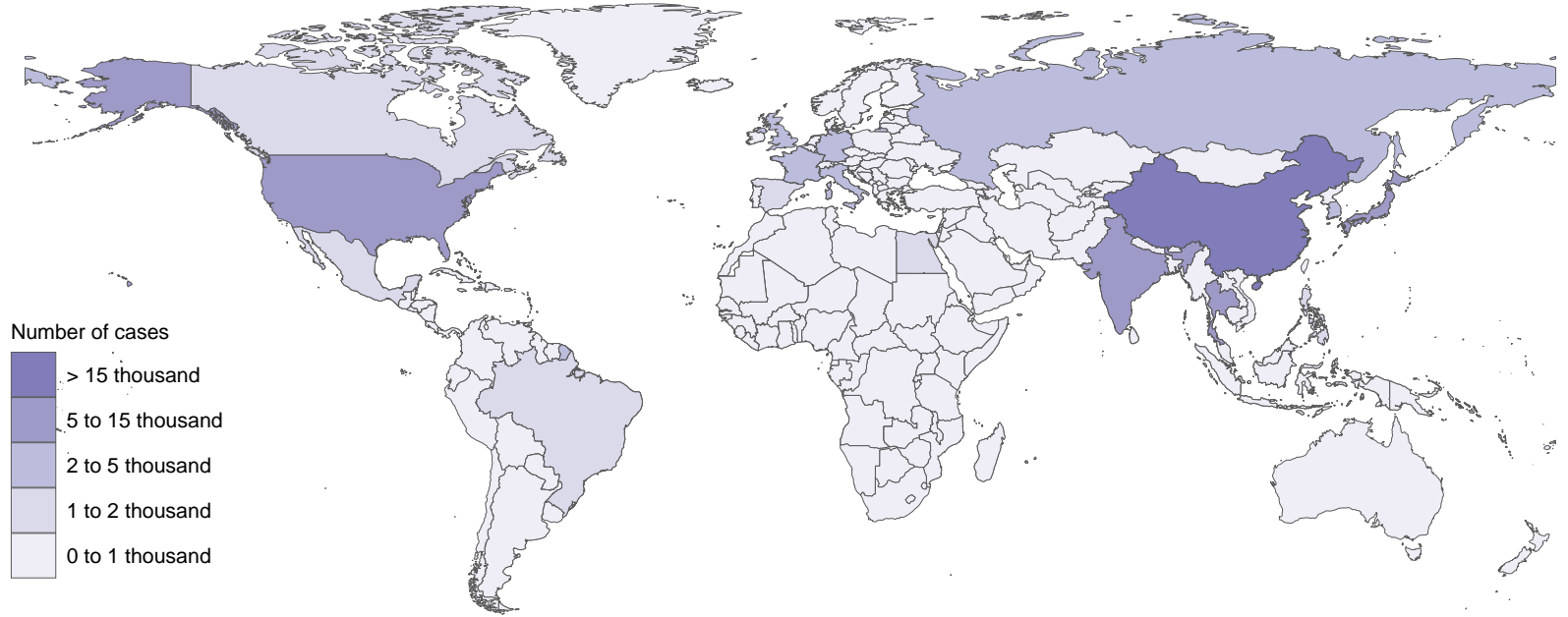

M

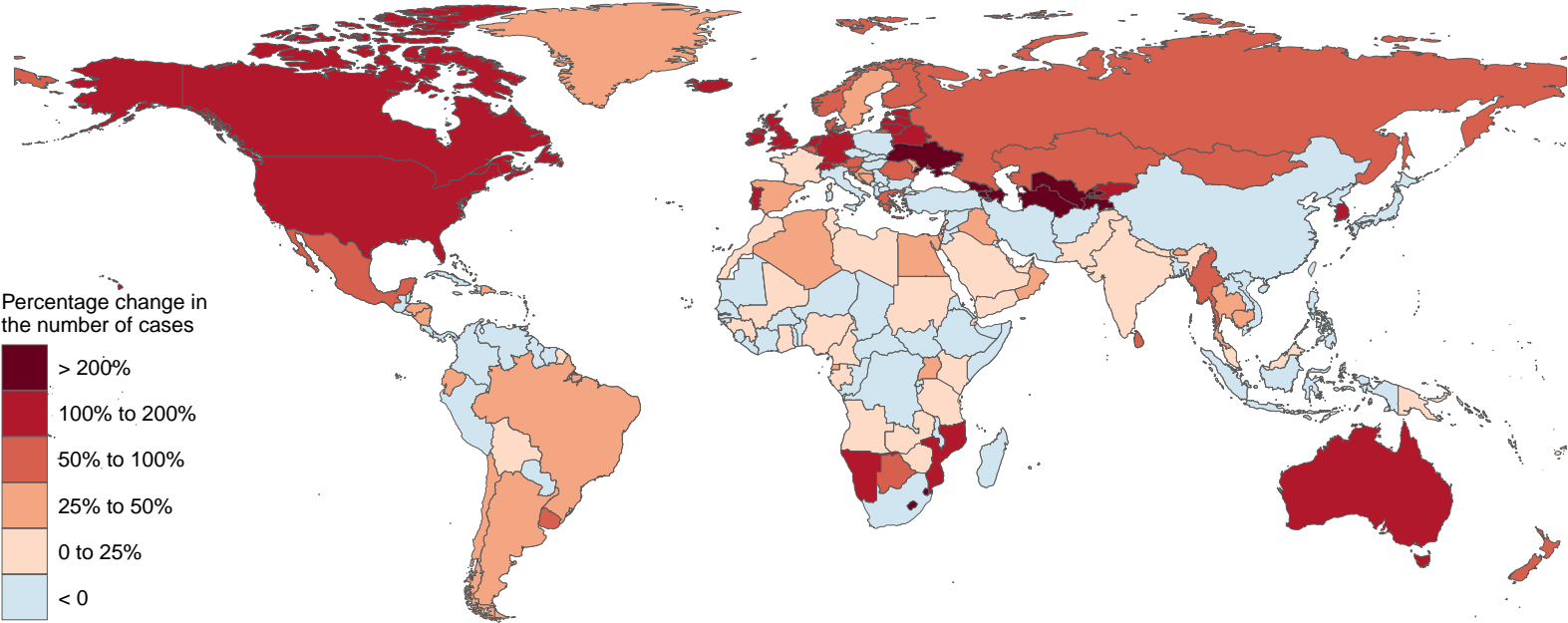

N

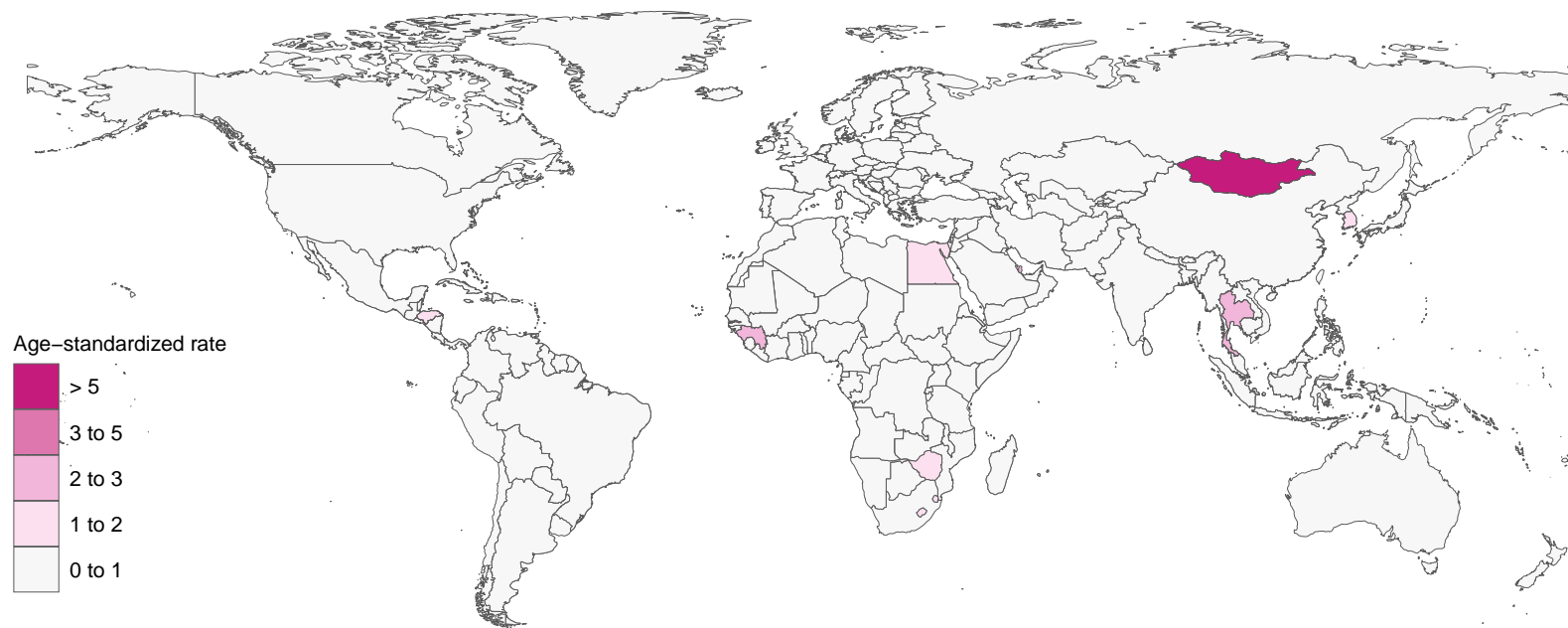

0

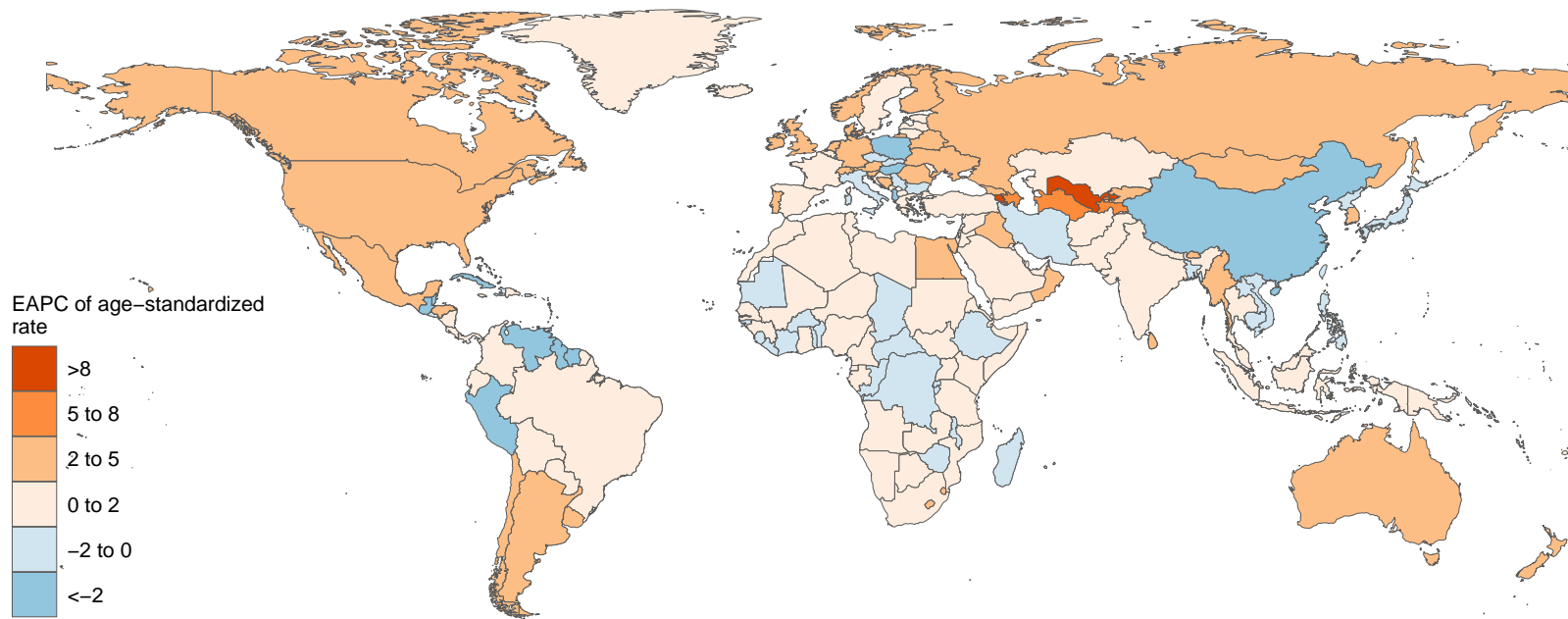

P

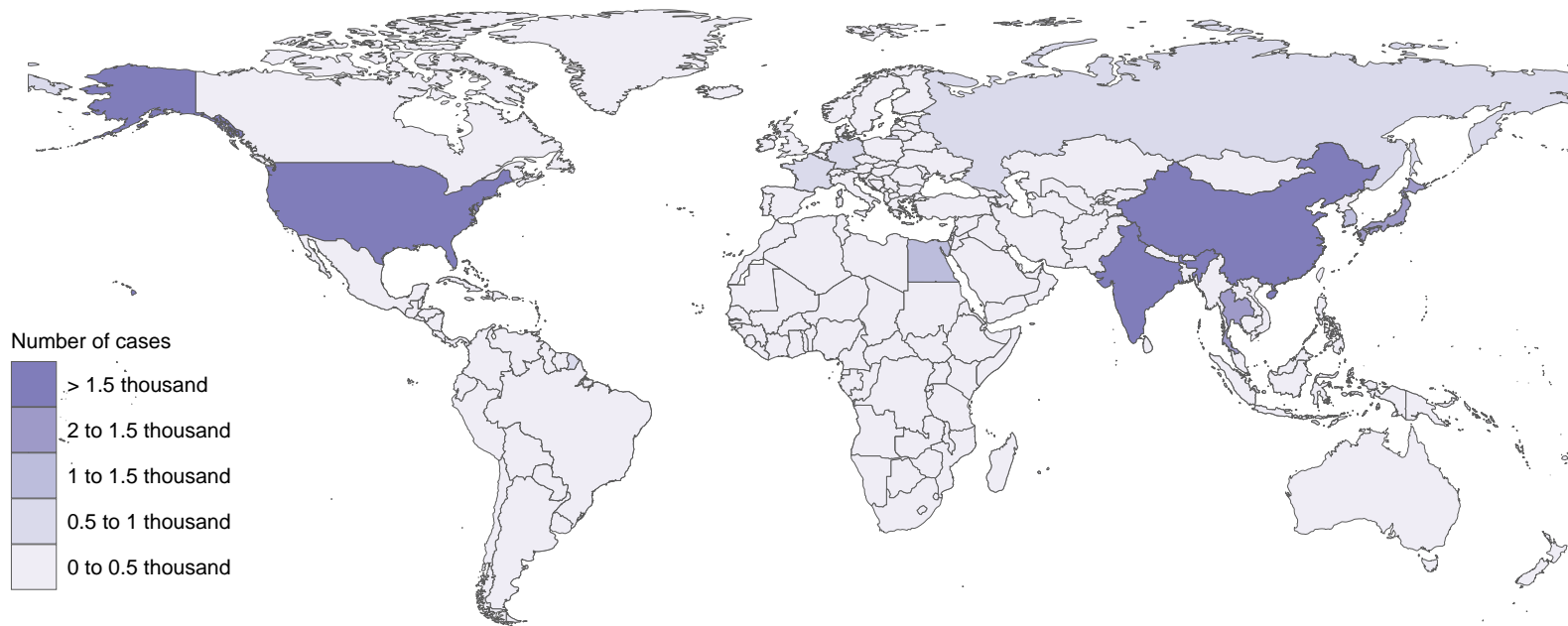

Q

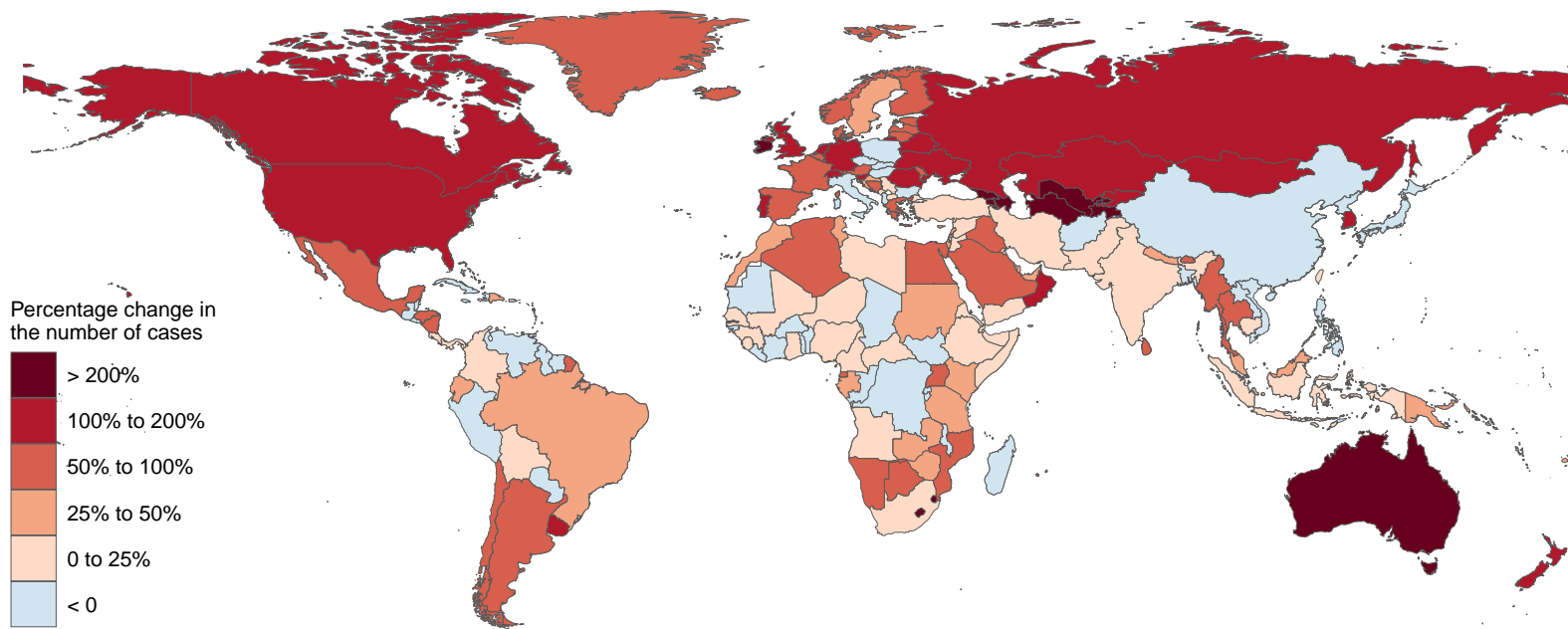

R

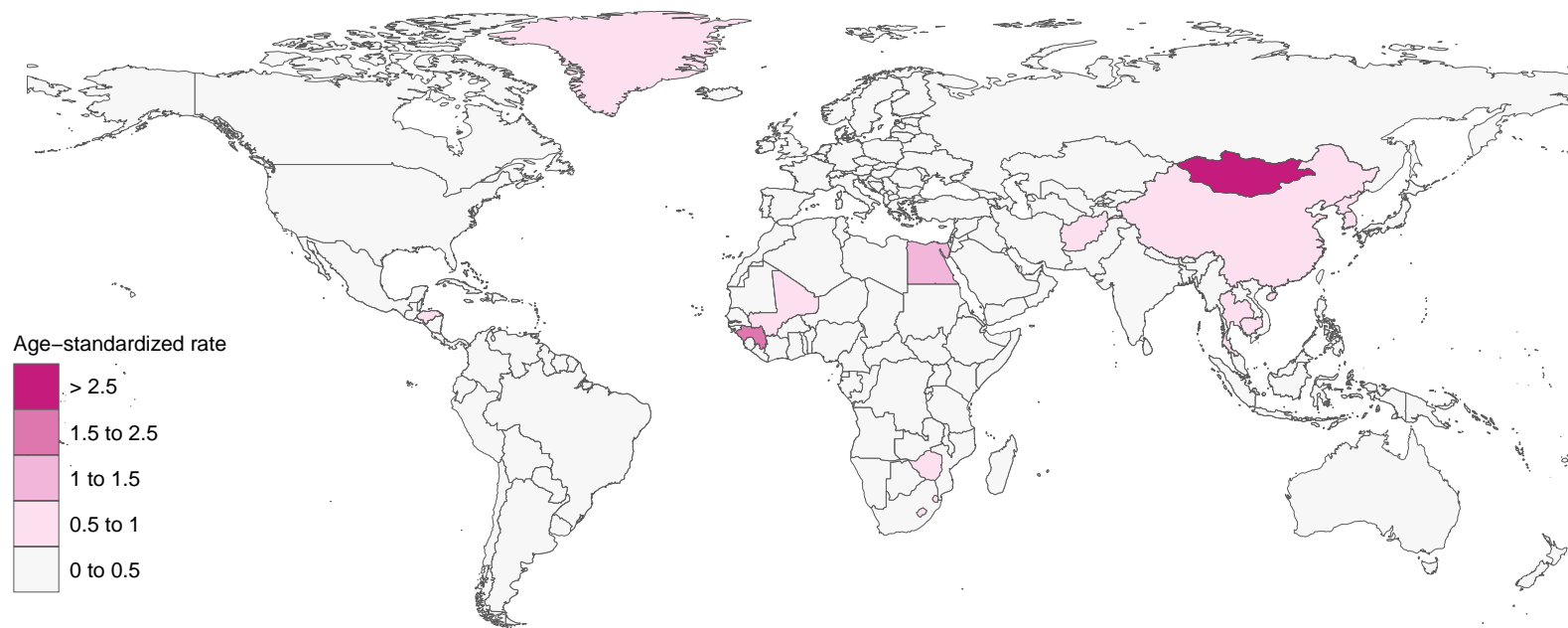

S

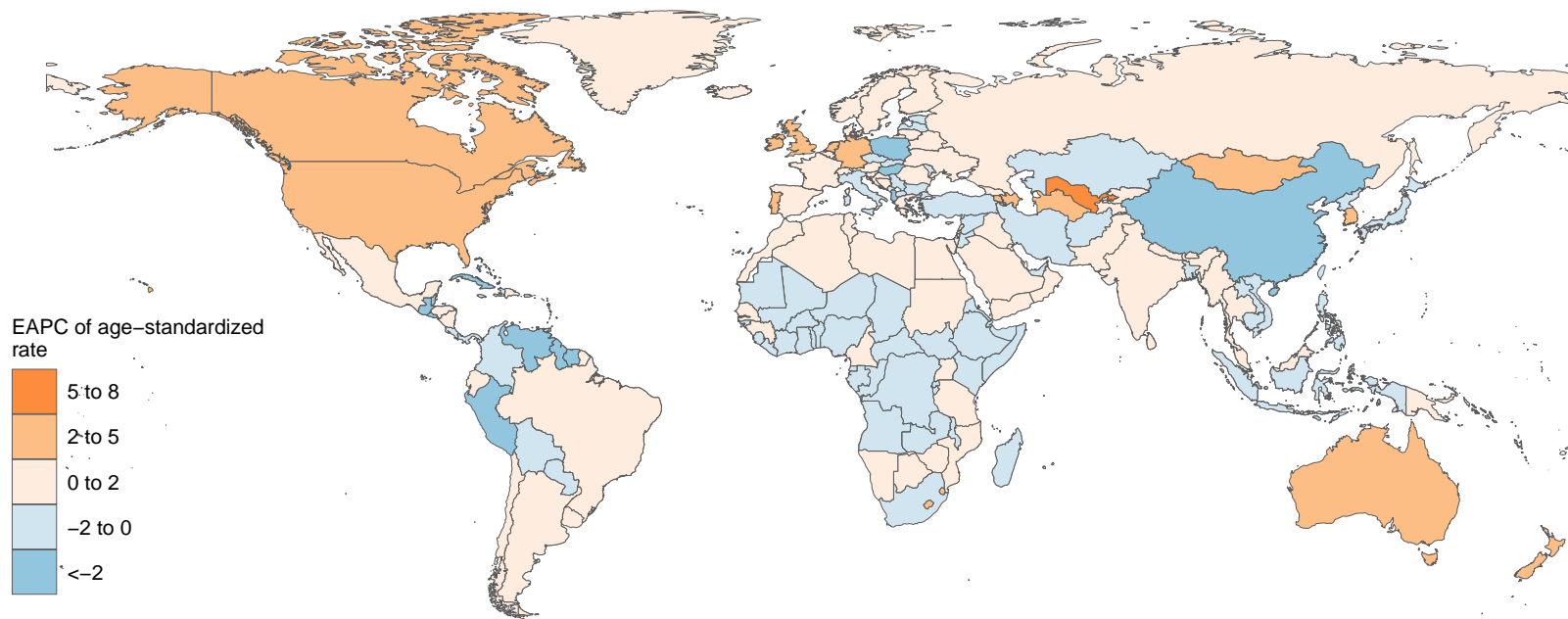

T

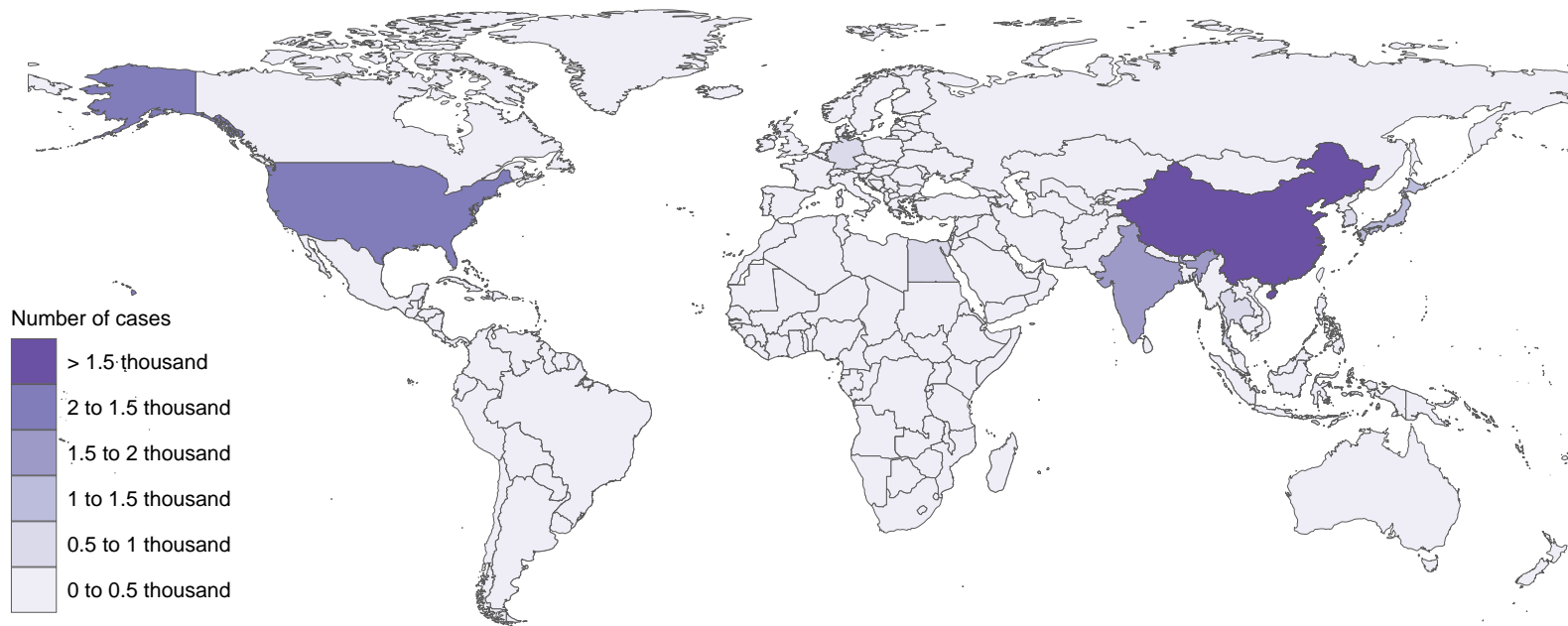

U

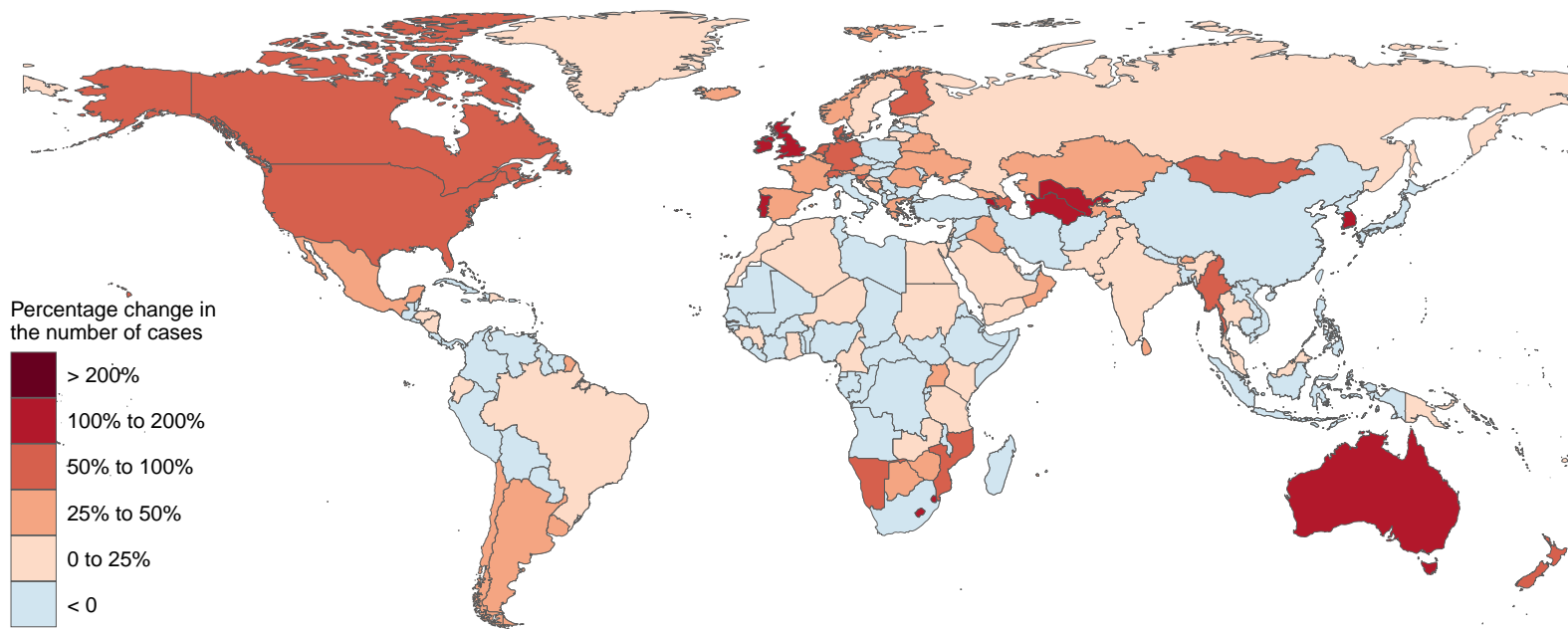

Supplement: Supplementary file 1 — Figure S1 [file CAM4-11-1310-s001.pdf]

A

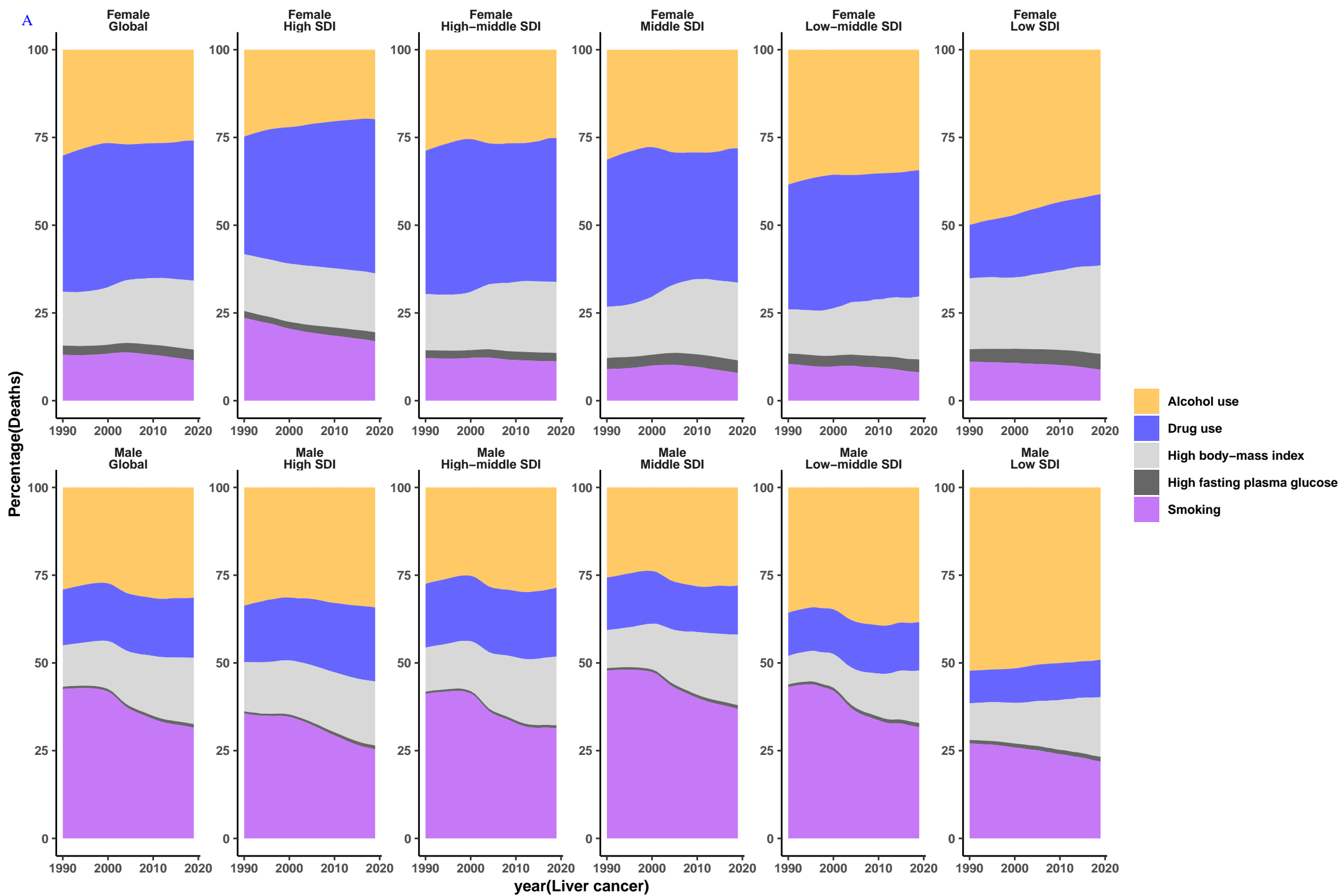

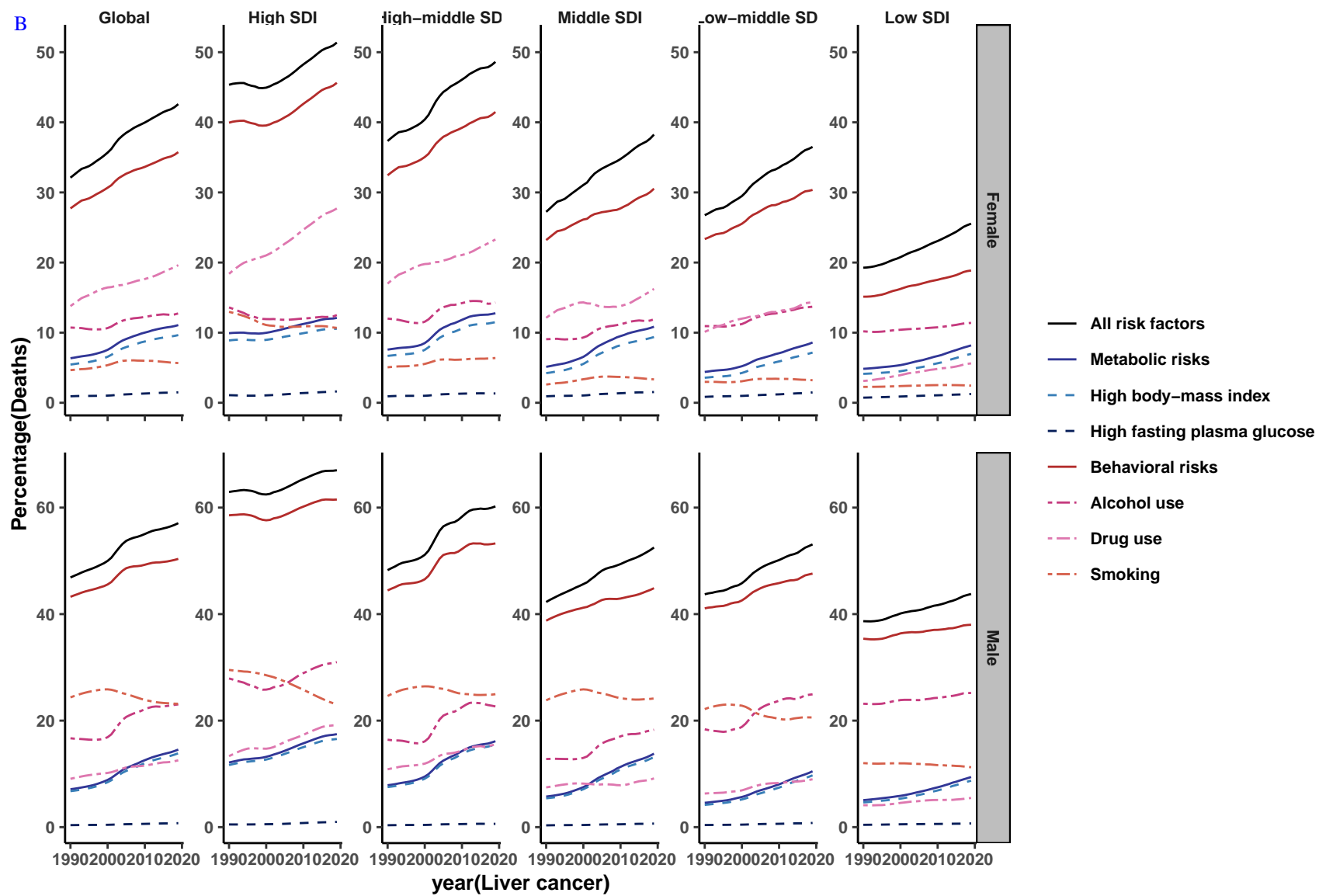

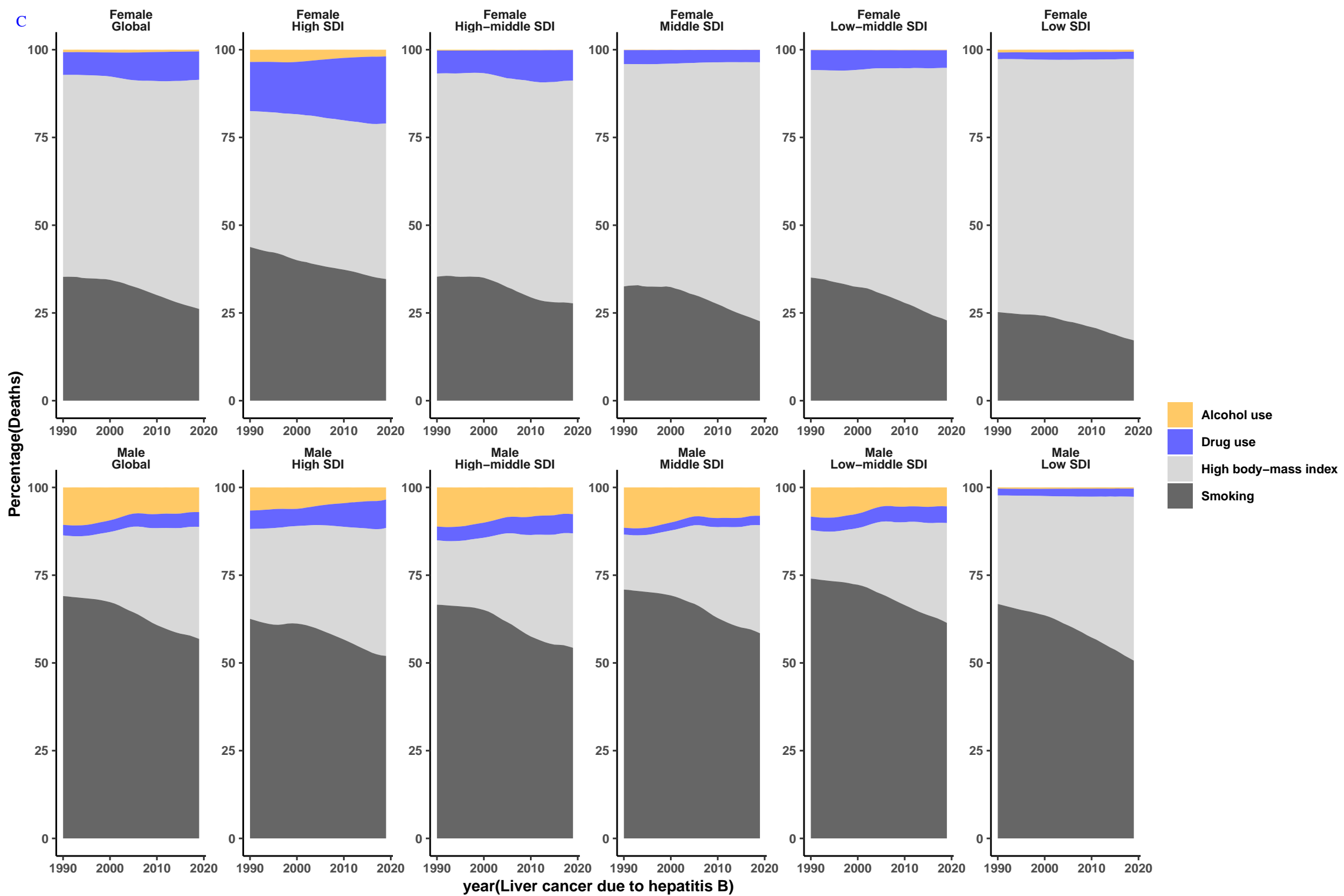

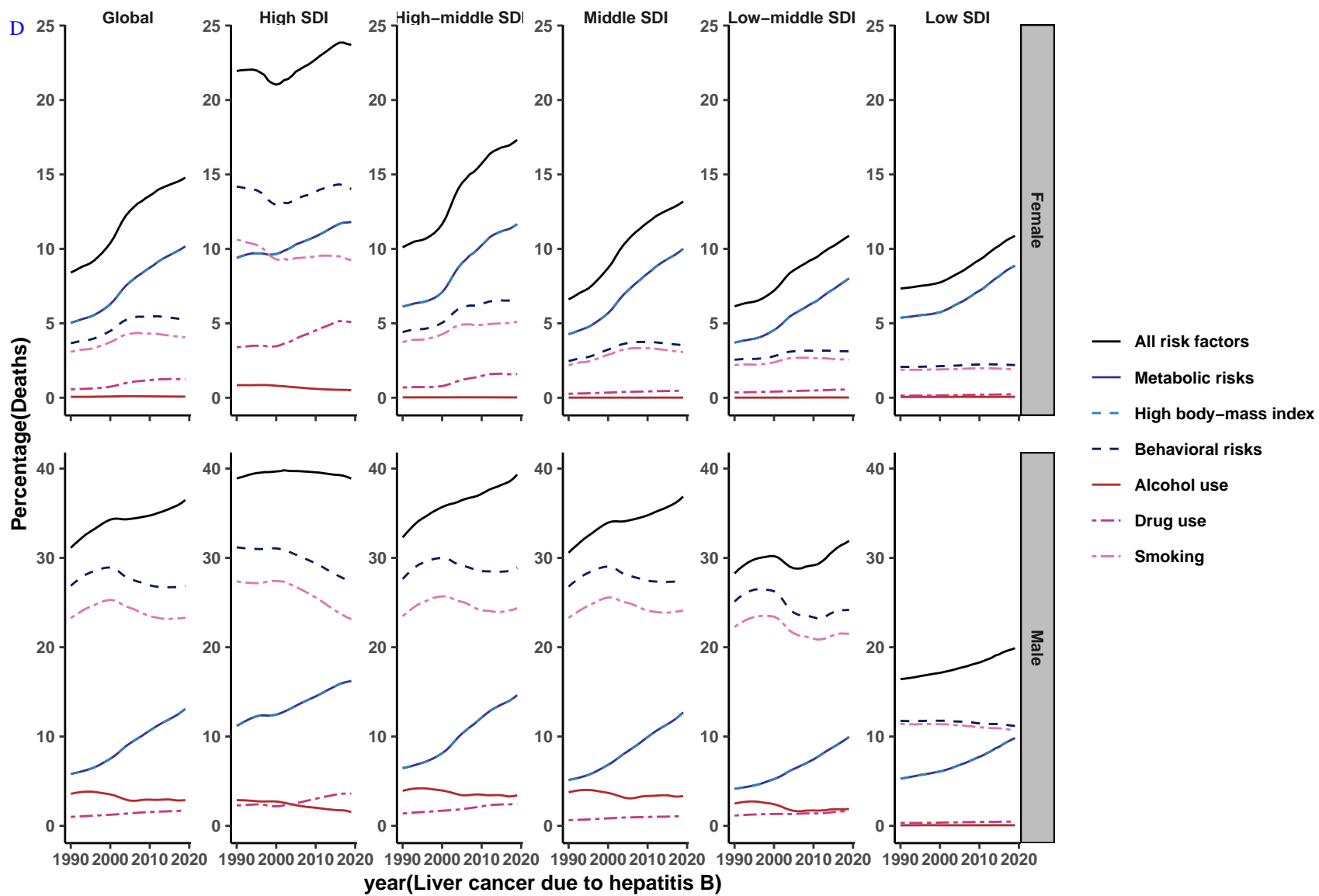

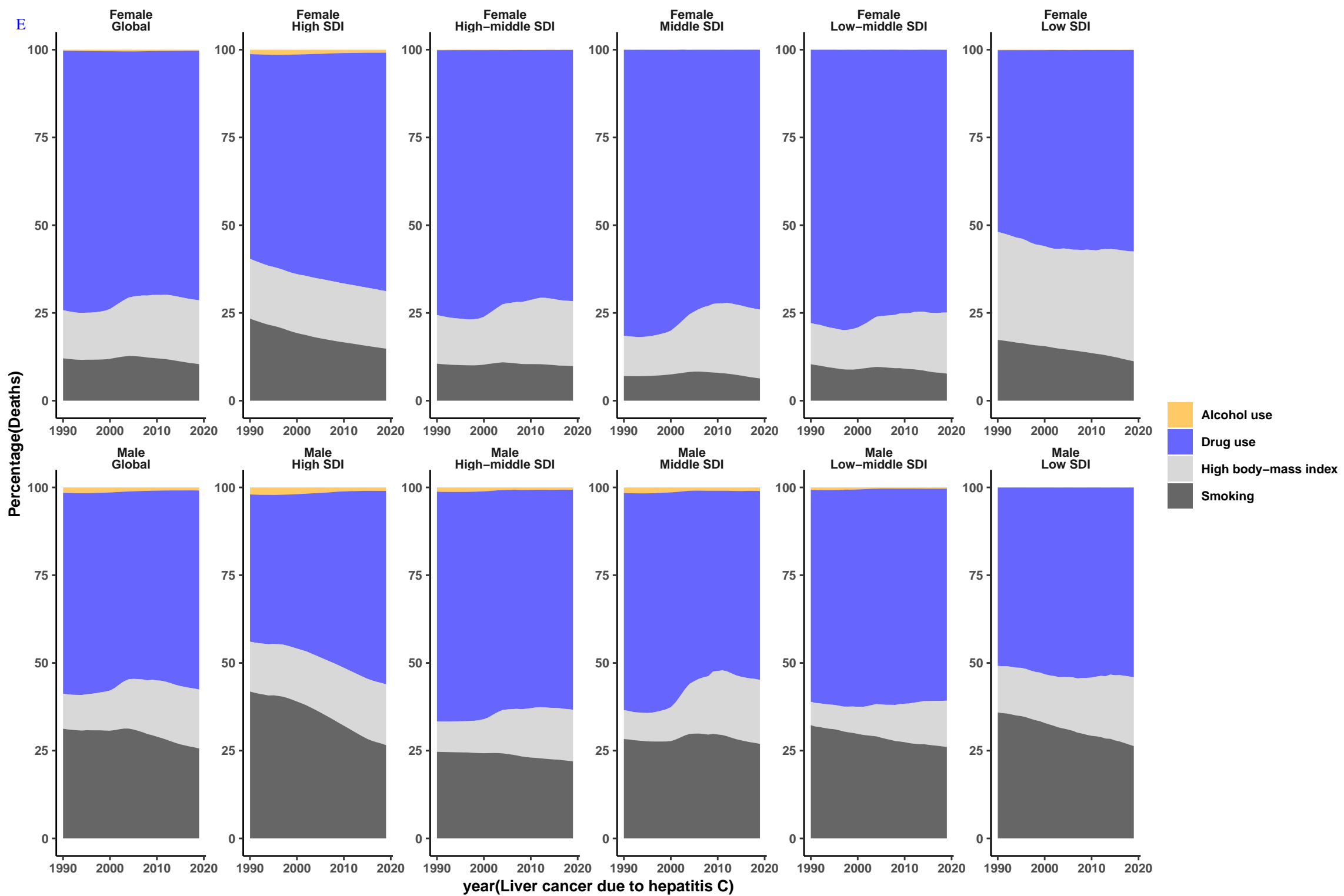

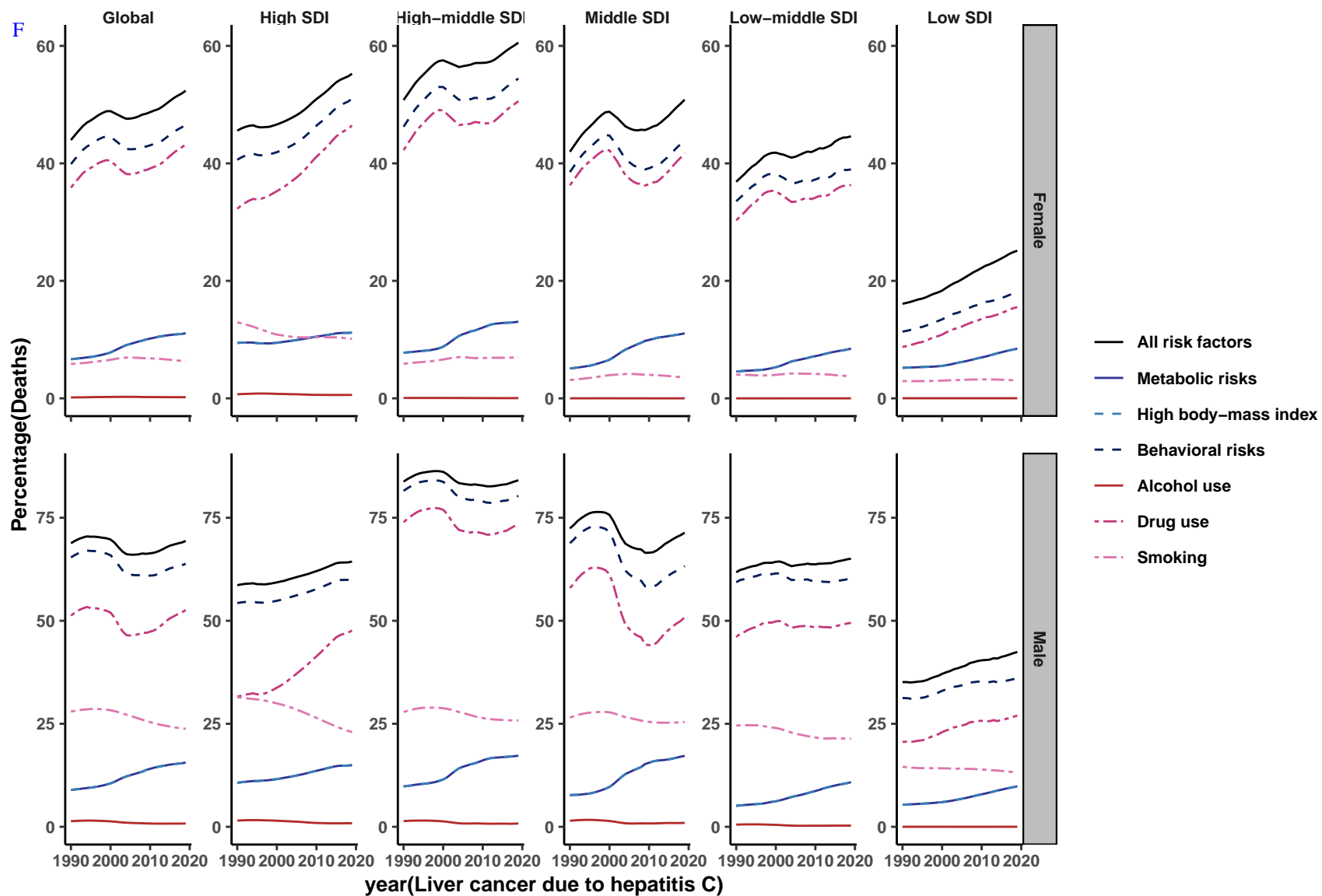

G

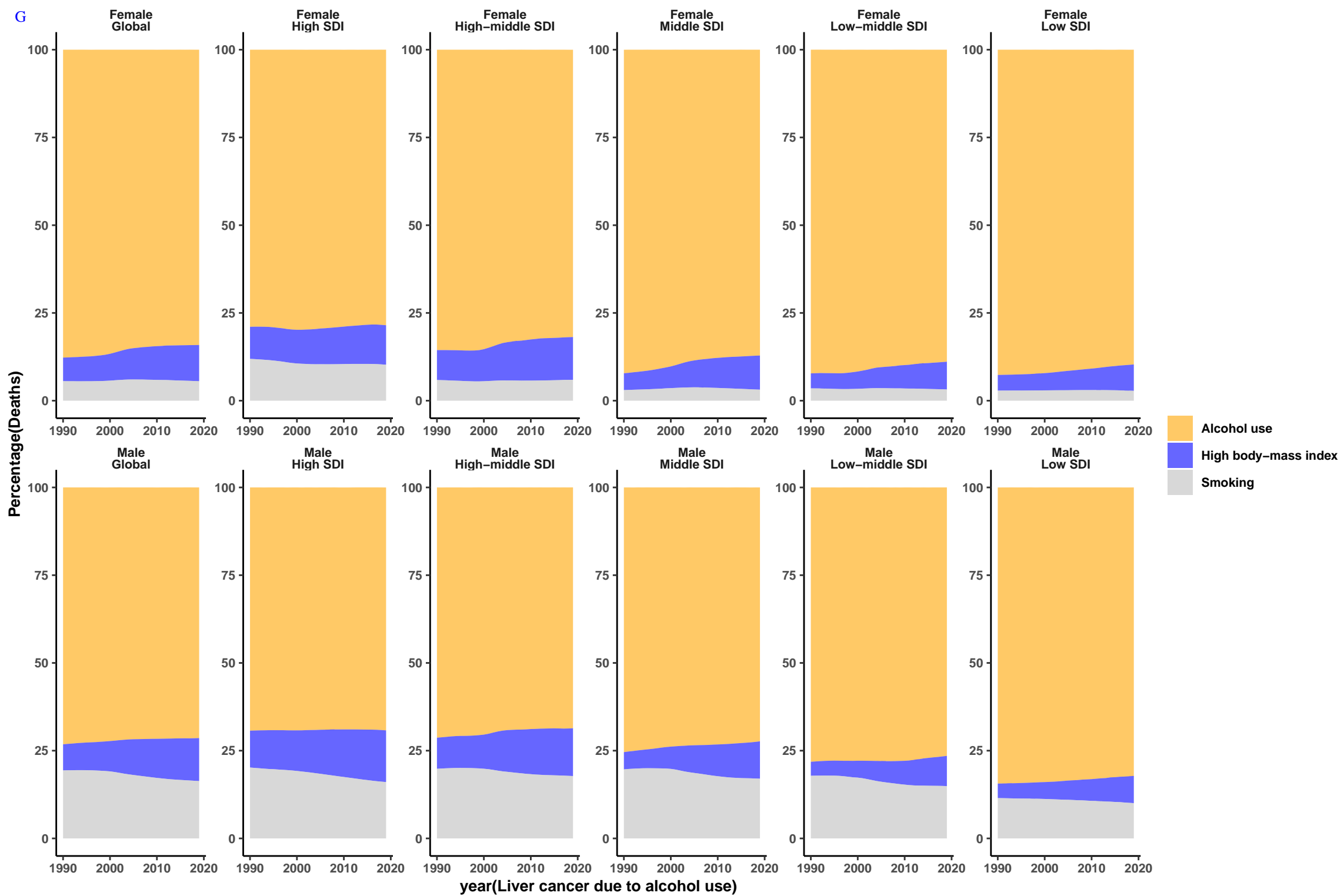

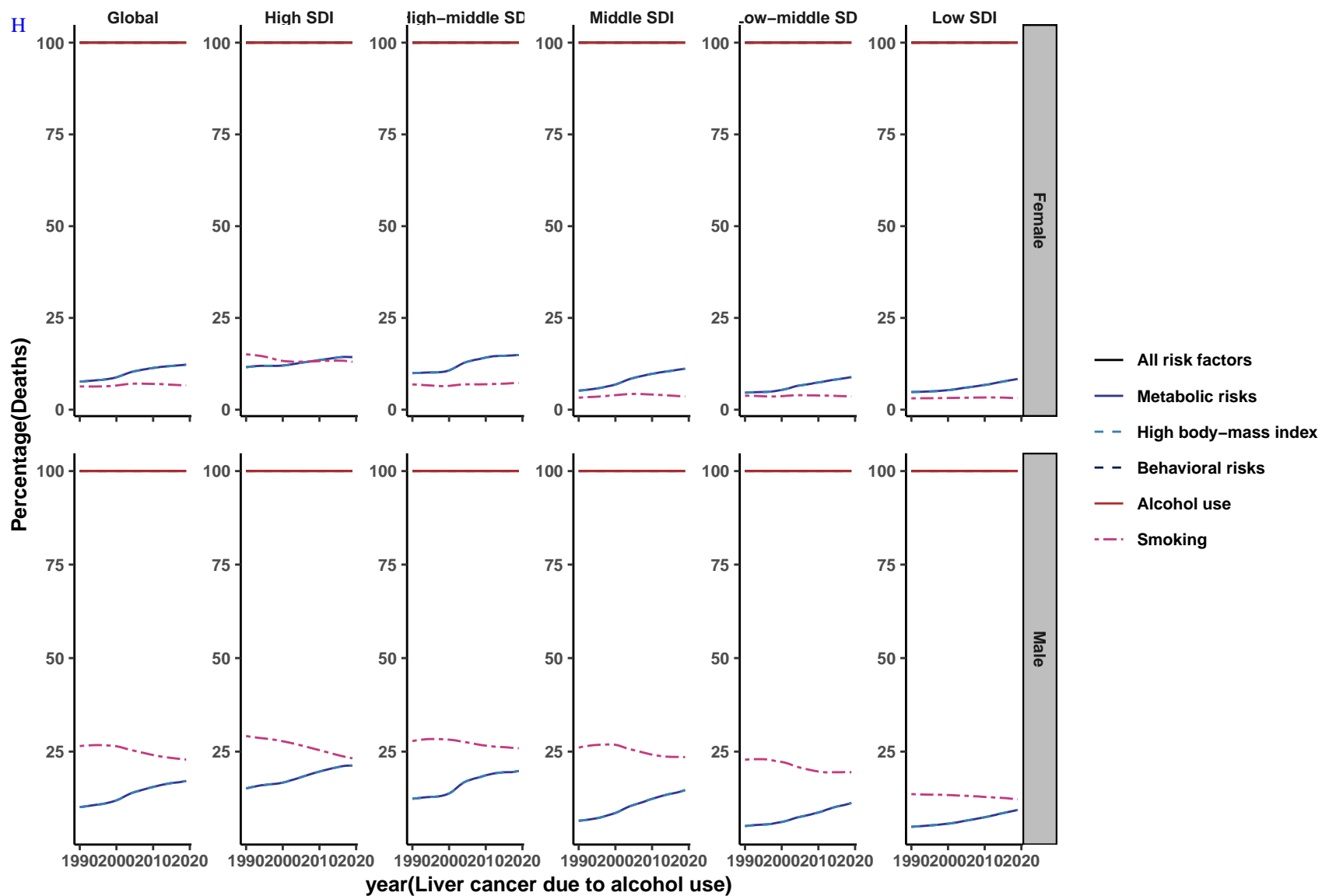

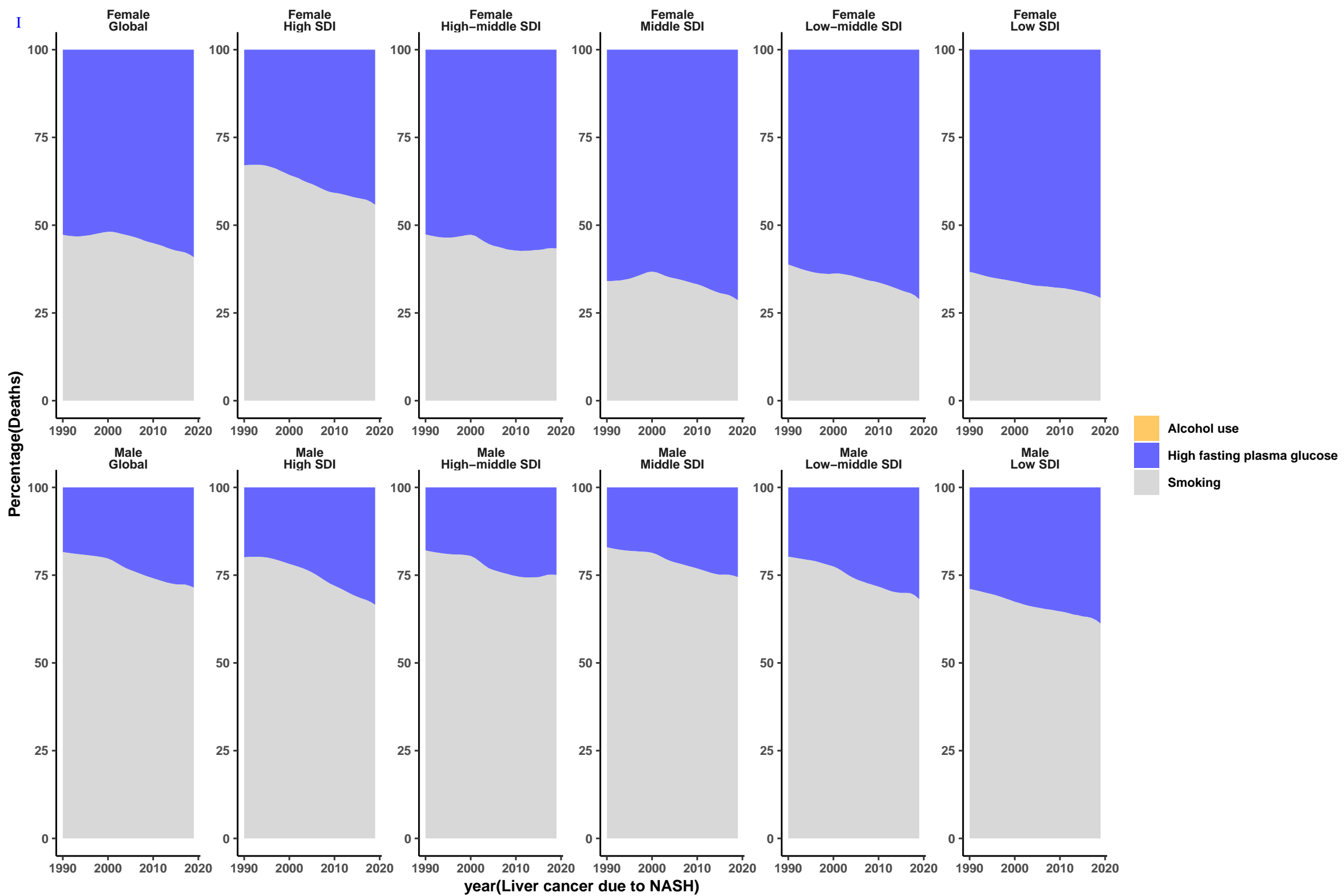

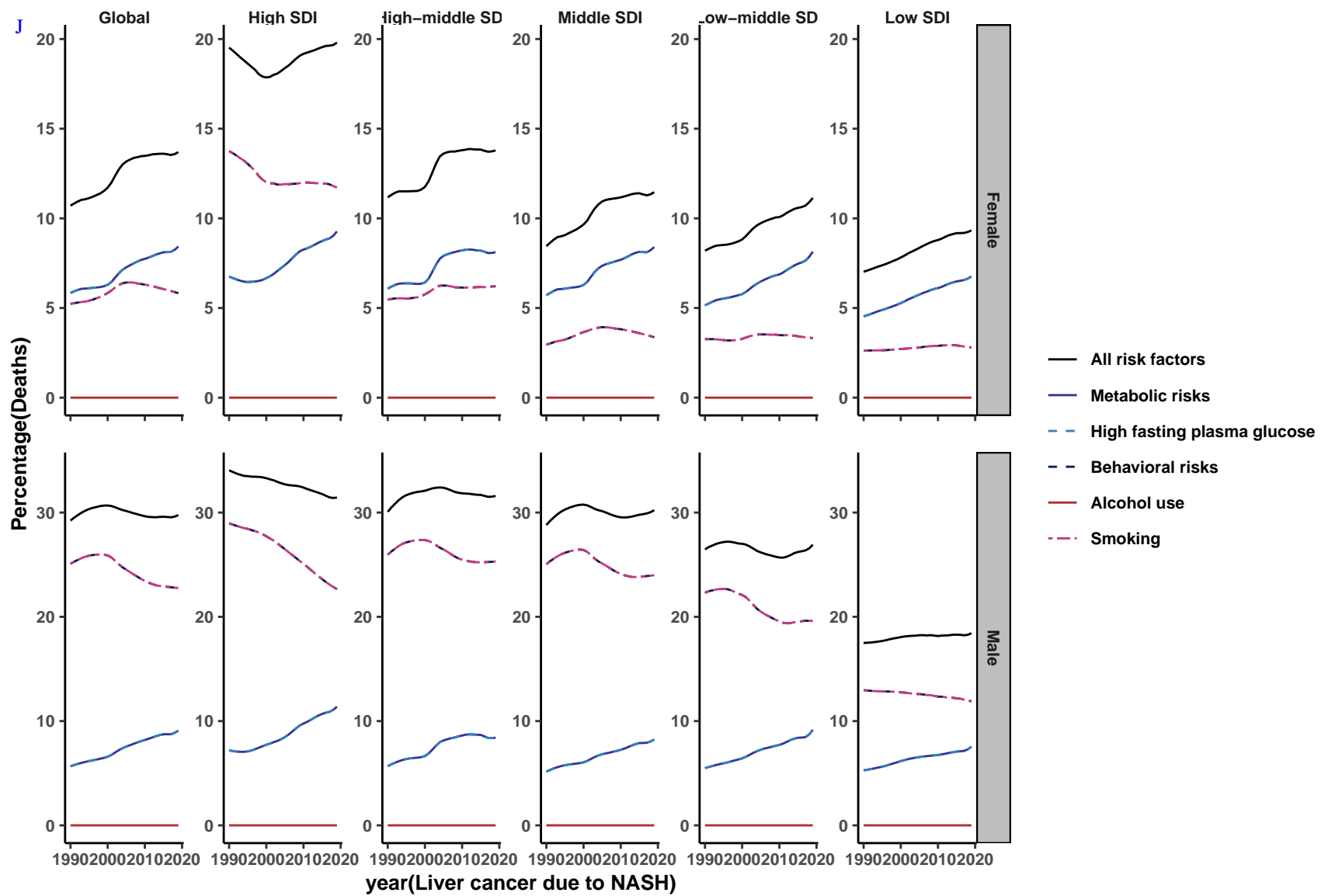

K

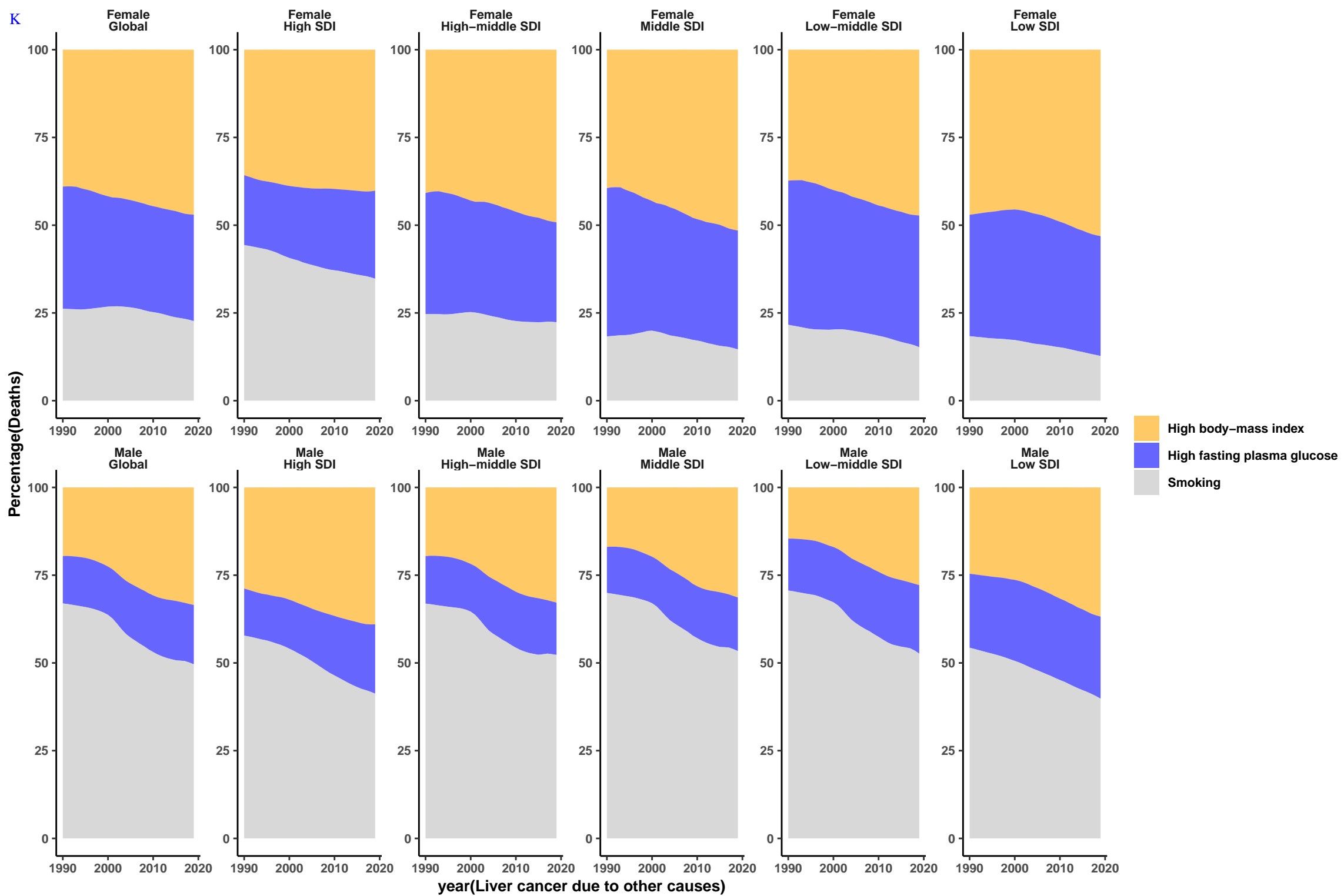

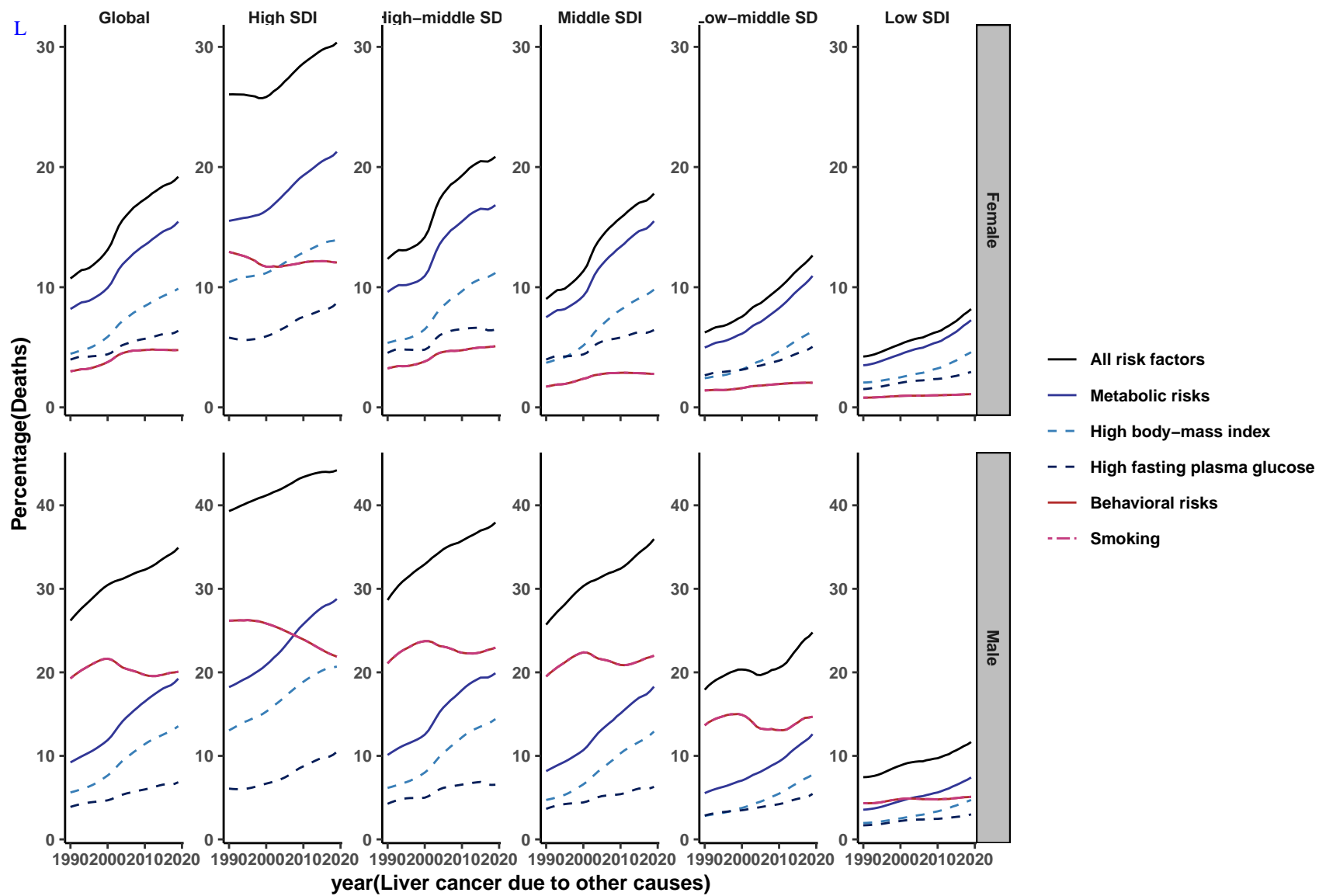

Supplement: Supplementary file 2 — Figure S2 [file CAM4-11-1310-s002.pdf]

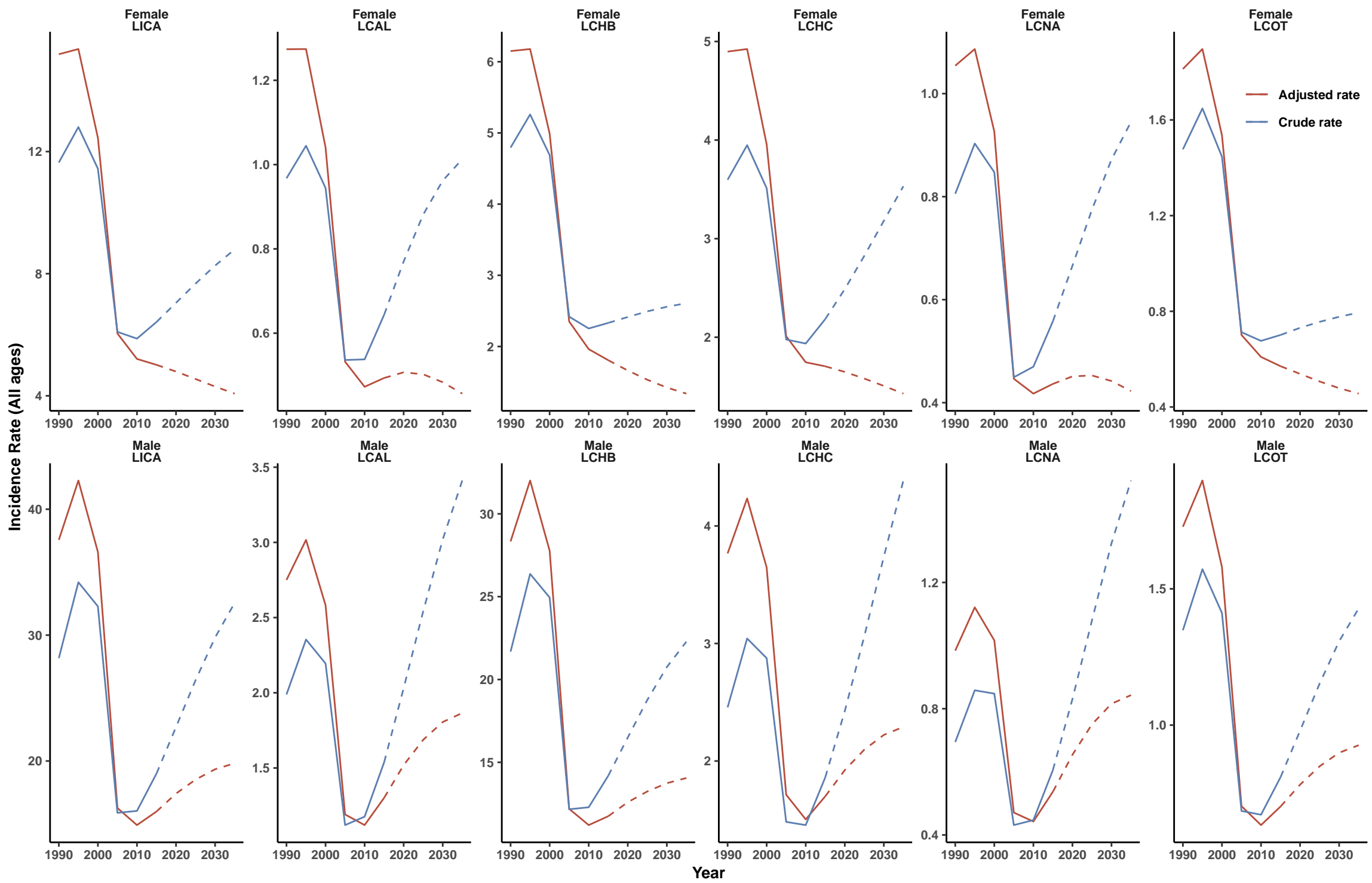

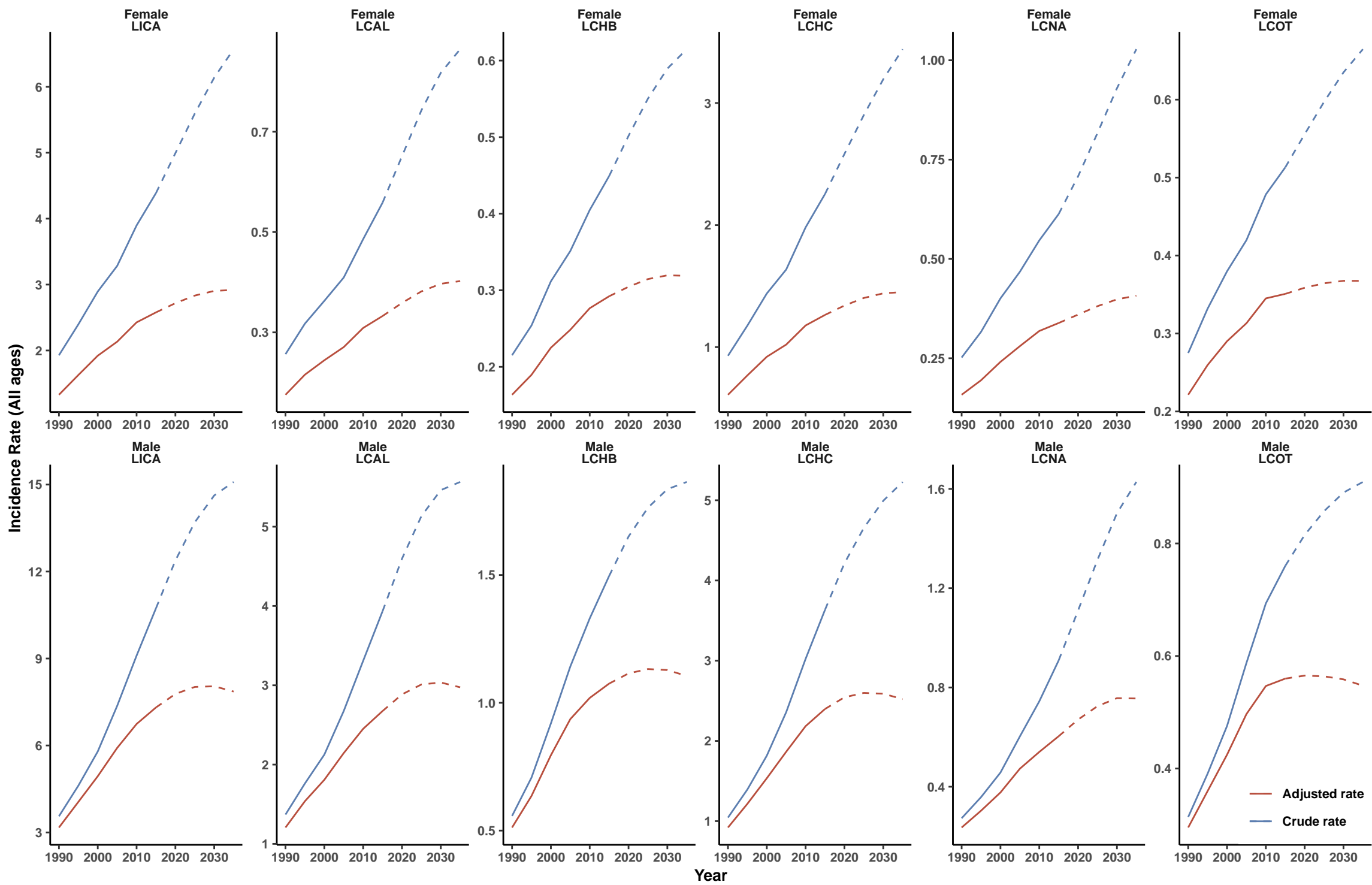

Supplement: Supplementary file 5 — Figure S5 [file CAM4-11-1310-s004.pdf]
