# Supplementary material for: Global burden of primary liver cancer by five etiologies and global prediction by 2035 based on global burden of disease study 2019
Source: Cancer Med. 2022 Feb 4;11(5):1310–23. doi: 10.1002/cam4.4551 (PMC8894698; doi:10.1002/cam4.4551)

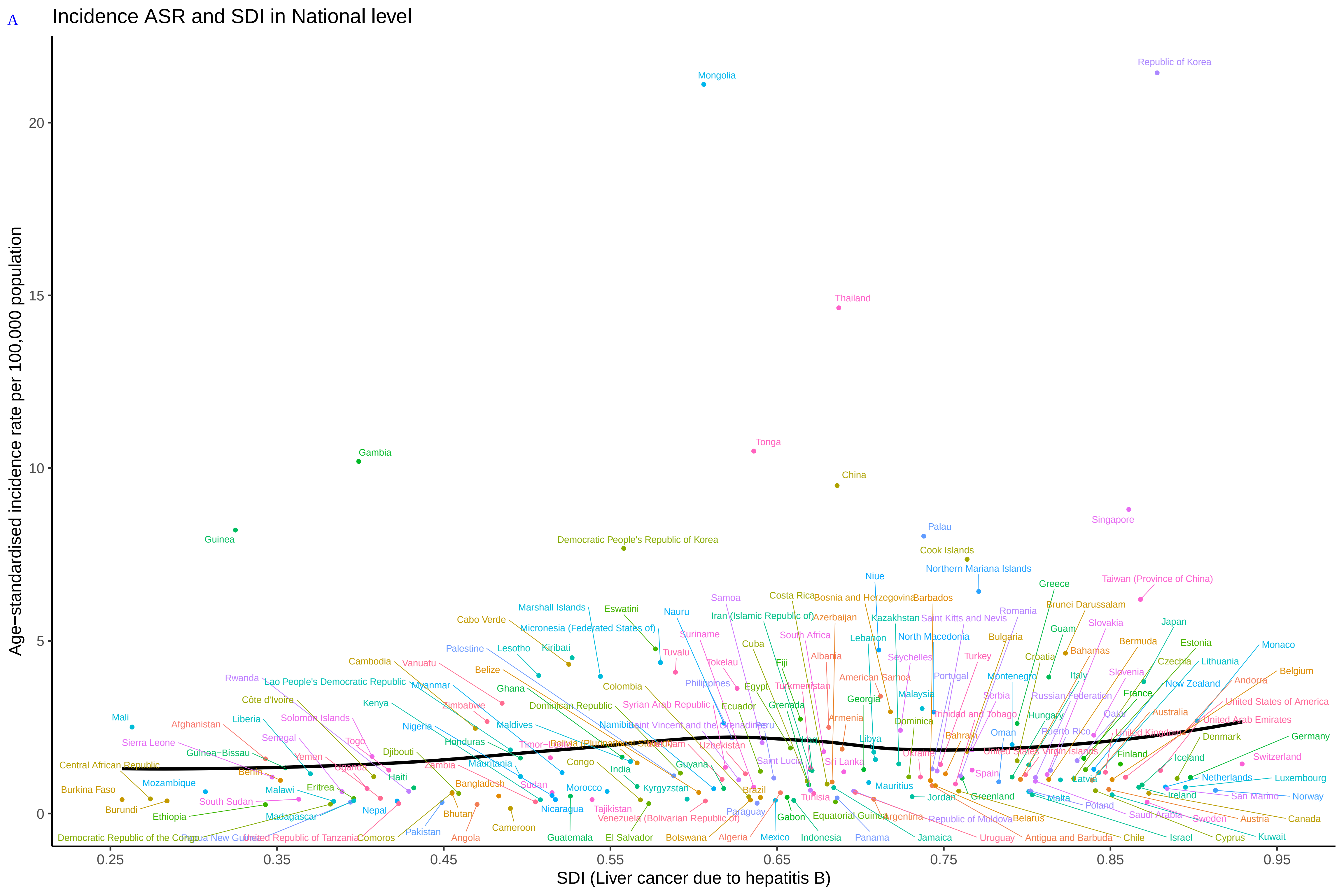
A
Incidence ASR and SDI in National level
Age−standardised incidence rate per 100,000 population
SDI (Liver cancer due to hepatitis B)
0
5
10
15
20
0.25
0.35
0.45
0.55
0.65
0.75
0.85
0.95
Republic of Korea
Mongolia
Thailand
Tonga
China
Gambia
Guinea
Singapore
Palau
Democratic People's Republic of Korea
Cook Islands
Northern Mariana Islands
Niue
Greece
Taiwan (Province of China)
Samoa
Costa Rica
Bosnia and Herzegovina
Barbados
Brunei Darussalam
Marshall Islands
Eswatini
Nauru
Romania
Cabo Verde
Iran (Islamic Republic of)
Azerbaijan
Kazakhstan
Saint Kitts and Nevis
Micronesia (Federated States of)
Suriname
South Africa
Lebanon
North Macedonia
Bulgaria
Guam
Slovakia
Japan
Palestine
Lesotho
Kiribati
Cuba
Bermuda
Estonia
Monaco
Bahamas
Tuvalu
Albania
Seychelles
Turkey
Croatia
Cambodia
Vanuatu
Fiji
Tokelau
Czechia
Lithuania
Belize
Italy
Slovenia
Belgium
American Samoa
Portugal
Montenegro
Philippines
Egypt
Turkmenistan
Andorra
Rwanda
Lao People's Democratic Republic
Myanmar
Ghana
New Zealand
Colombia
Georgia
Malaysia
Serbia
Russian Federation
France
Côte d'Ivoire
Kenya
Zimbabwe
Dominican Republic
Syrian Arab Republic
Ecuador
Grenada
United States of America
Trinidad and Tobago
Hungary
Qatar
Australia
Mali
Liberia
Solomon Islands
Maldives
Armenia
Dominica
Nigeria
Namibia
Saint Vincent and the Grenadines
Peru
United Arab Emirates
Afghanistan
Puerto Rico
Oman
Bahrain
United Kingdom
Germany
Togo
Senegal
Sierra Leone
Honduras
Libya
Denmark
Djibouti
Uzbekistan
Iraq
Guinea−Bissau
Ukraine
United States Virgin Islands
Finland
Yemen
Saint Lucia
Sri Lanka
Iceland
Switzerland
Central African Republic
Uganda
Mauritania
Zambia
Congo
Guyana
India
Benin
Haiti
Spain
Netherlands
Luxembourg
Mozambique
Sudan
Bangladesh
Kyrgyzstan
Brazil
Mauritius
Latvia
Eritrea
Malawi
Morocco
Burkina Faso
Tunisia
Jordan
Greenland
Ireland
San Marino
Norway
Malta
South Sudan
Poland
Burundi
Ethiopia
Madagascar
Nepal
Bhutan
Nicaragua
Tajikistan
Paraguay
Equatorial Guinea
Argentina
Saudi Arabia
Sweden
Austria
Canada
Gabon
Republic of Moldova
Belarus
Venezuela (Bolivarian Republic of)
Cameroon
Pakistan
Democratic Republic of the Congo
United Republic of Tanzania
Comoros
Angola
Guatemala
El Salvador
Botswana
Algeria
Mexico
Indonesia
Panama
Jamaica
Uruguay
Antigua and Barbuda
Chile
Israel
Cyprus
Kuwait

B

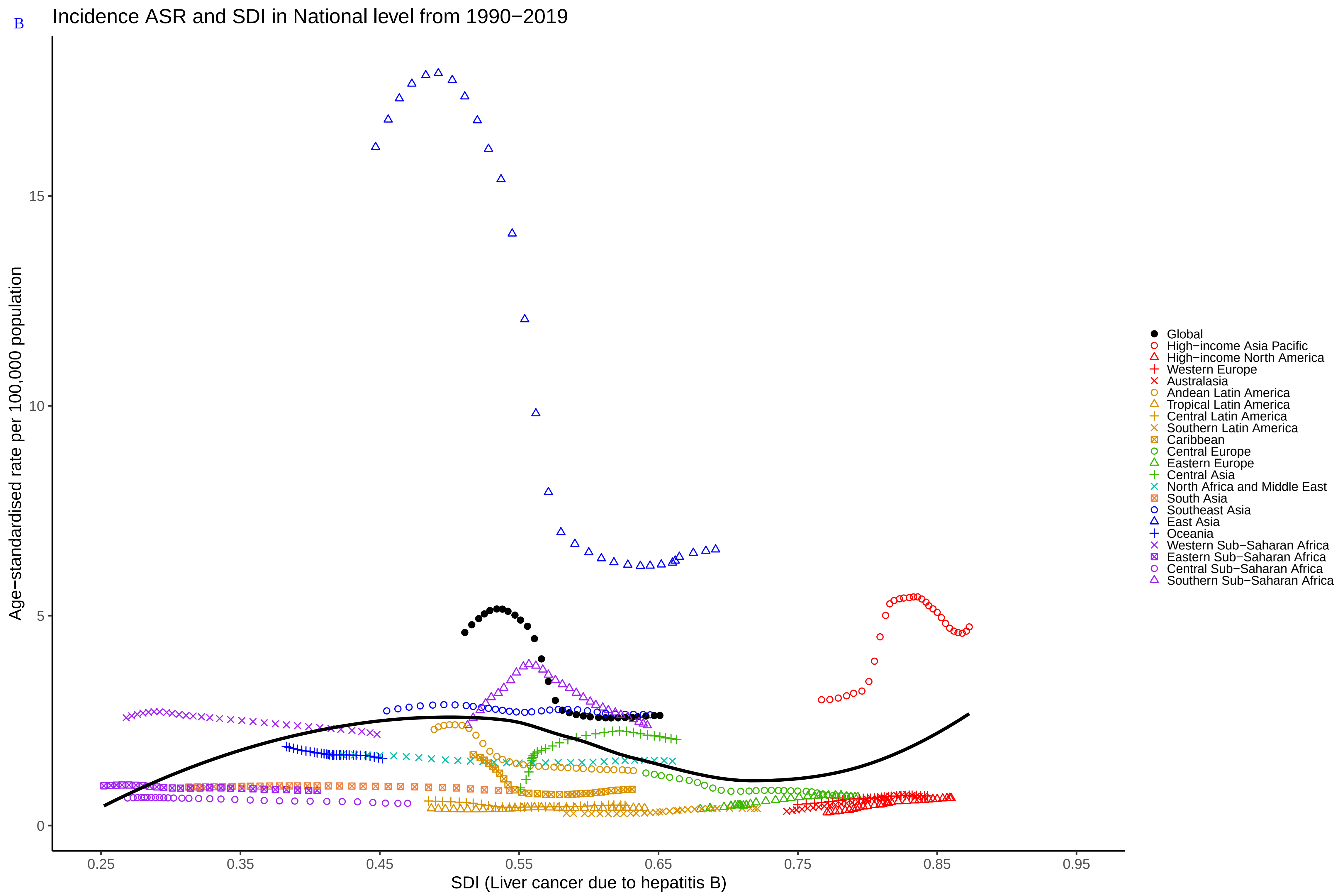

Incidence ASR and SDI in National level from 1990−2019
Age−standardised rate per 100,000 population
0
5
10
15
0.25
0.35
0.45
0.55
0.65
0.75
0.85
0.95
SDI (Liver cancer due to hepatitis B)
Global
High−income Asia Pacific
High−income North America
Western Europe
Australasia
Andean Latin America
Tropical Latin America
Central Latin America
Southern Latin America
Caribbean
Central Europe
Eastern Europe
Central Asia
North Africa and Middle East
South Asia
Southeast Asia
East Asia
Oceania
Western Sub−Saharan Africa
Eastern Sub−Saharan Africa
Central Sub−Saharan Africa
Southern Sub−Saharan Africa

C

Incidence ASR and SDI in National level

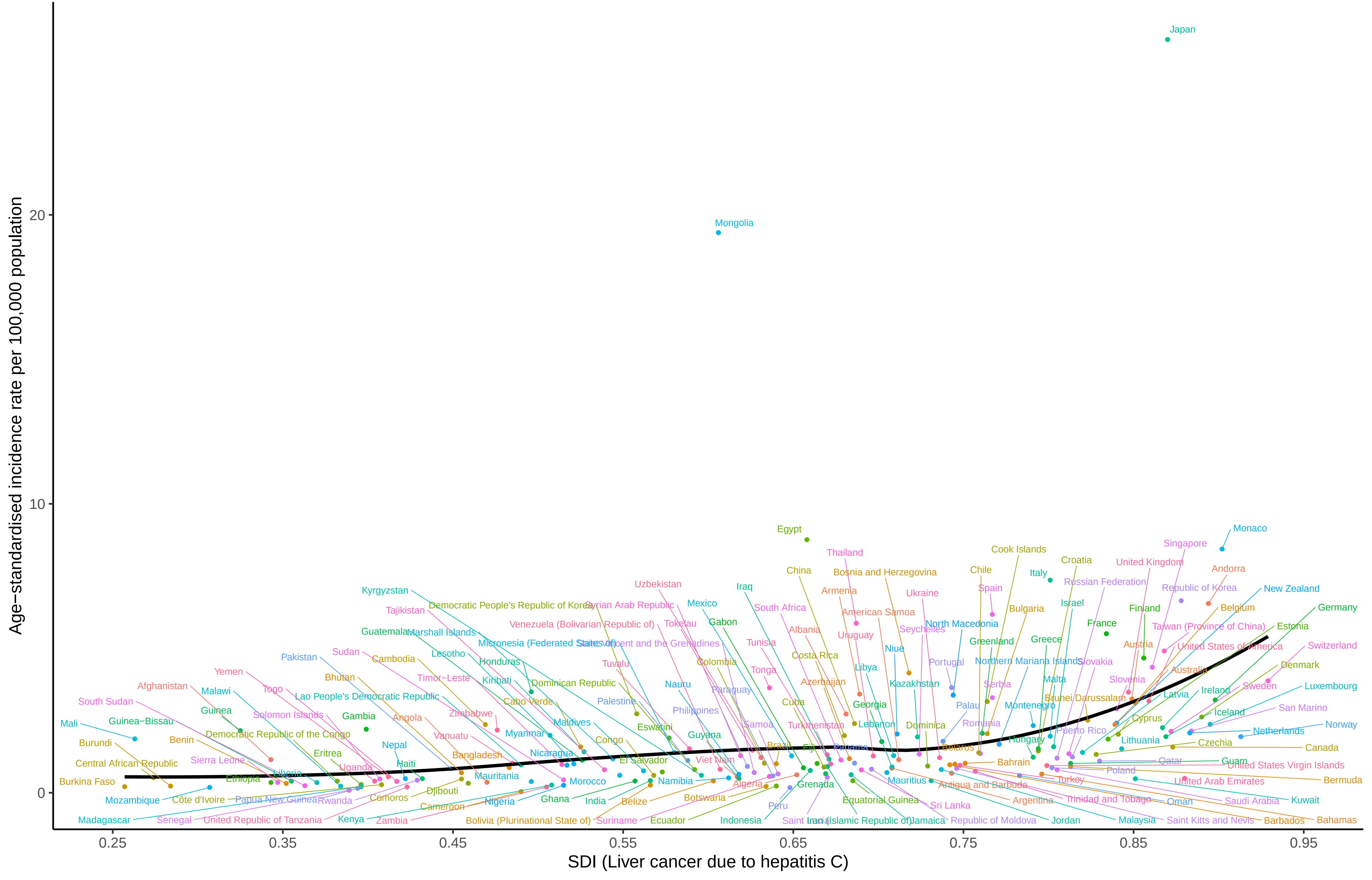

D

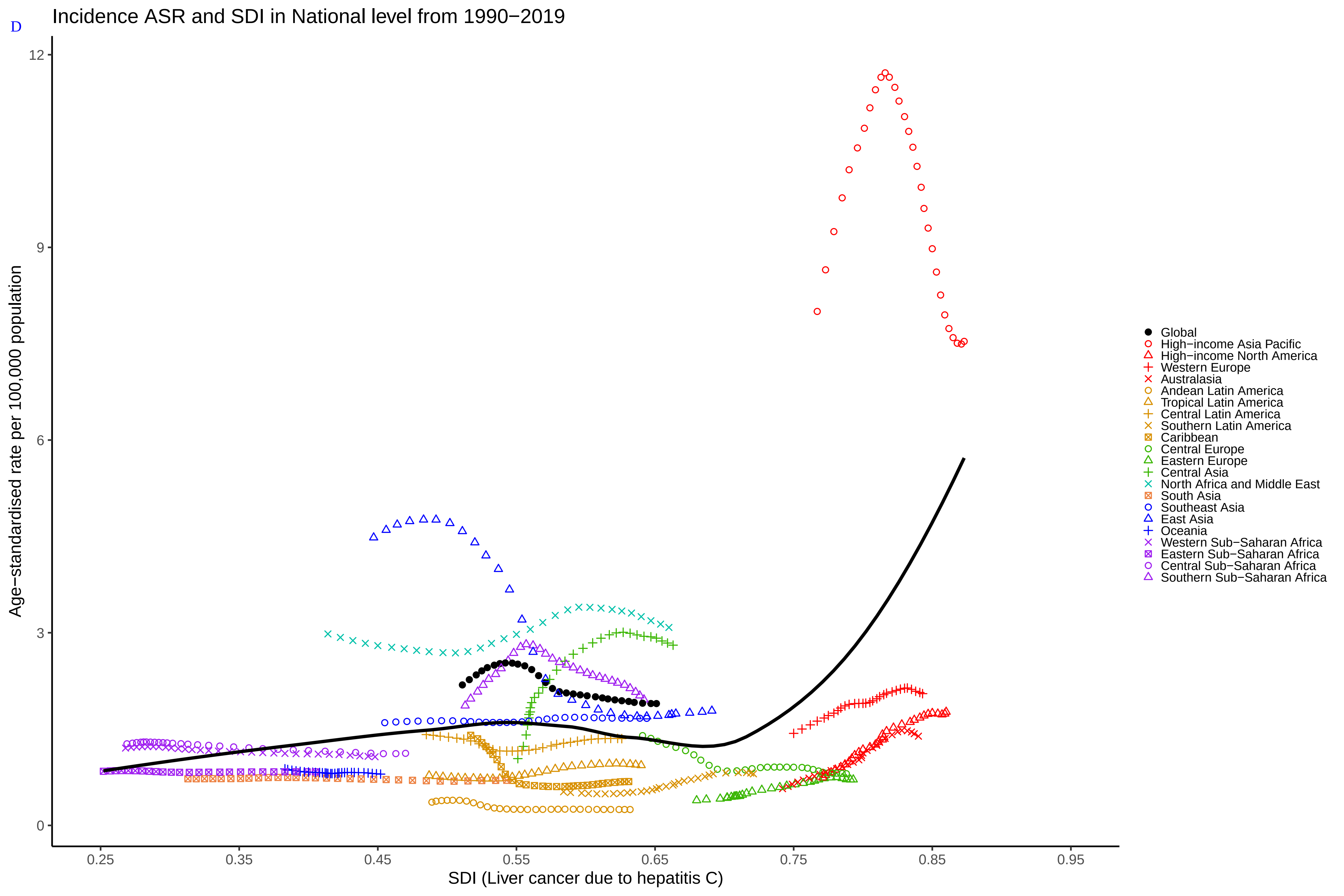
Incidence ASR and SDI in National level from 1990−2019
Age−standardised rate per 100,000 population
12
9
6
3
0
0.25
0.35
0.45
0.55
0.65
0.75
0.85
0.95
SDI (Liver cancer due to hepatitis C)
Global
High−income Asia Pacific
High−income North America
Western Europe
Australasia
Andean Latin America
Tropical Latin America
Central Latin America
Southern Latin America
Caribbean
Central Europe
Eastern Europe
Central Asia
North Africa and Middle East
South Asia
Southeast Asia
East Asia
Oceania
Western Sub−Saharan Africa
Eastern Sub−Saharan Africa
Central Sub−Saharan Africa
Southern Sub−Saharan Africa

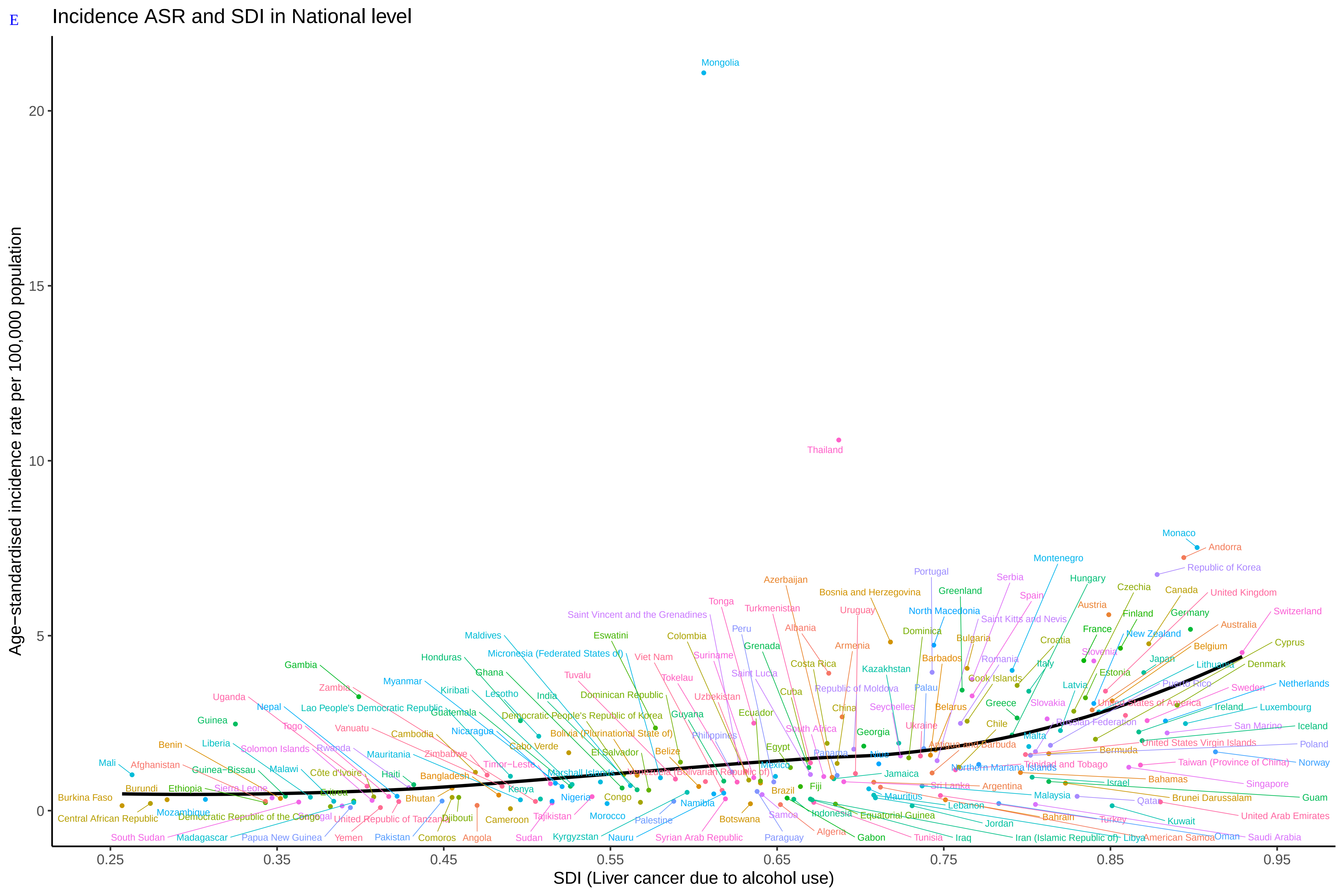

E
Incidence ASR and SDI in National level
Age−standardised incidence rate per 100,000 population
SDI (Liver cancer due to alcohol use)
0
5
10
15
20
0.25
0.35
0.45
0.55
0.65
0.75
0.85
0.95
Mongolia
Thailand
Monaco
Andorra
Republic of Korea
Montenegro
Portugal
Serbia
Hungary
Czechia
Canada
United Kingdom
Switzerland
Azerbaijan
Bosnia and Herzegovina
Greenland
Spain
Austria
Finland
Germany
Australia
Cyprus
Tonga
Turkmenistan
Uruguay
North Macedonia
Saint Kitts and Nevis
Dominica
France
New Zealand
Belgium
Denmark
Saint Vincent and the Grenadines
Peru
Albania
Bulgaria
Croatia
Slovenia
Japan
Lithuania
Maldives
Eswatini
Colombia
Grenada
Armenia
Barbados
Romania
Italy
Estonia
Puerto Rico
Sweden
Netherlands
Micronesia (Federated States of)
Viet Nam
Suriname
Costa Rica
Kazakhstan
Cook Islands
Latvia
United States of America
Ireland
Luxembourg
Gambia
Honduras
Ghana
Tuvalu
Tokelau
Saint Lucia
Republic of Moldova
Palau
Greece
Slovakia
Russian Federation
San Marino
Iceland
Zambia
Myanmar
Kiribati
Lesotho
India
Dominican Republic
Uzbekistan
Cuba
China
Seychelles
Belarus
Chile
Uganda
Nepal
Lao People's Democratic Republic
Guatemala
Democratic People's Republic of Korea
Guyana
Ecuador
Ukraine
Georgia
Malta
Bermuda
United States Virgin Islands
Poland
Guinea
Togo
Vanuatu
Cambodia
Nicaragua
Bolivia (Plurinational State of)
South Africa
Philippines
Antigua and Barbuda
Benin
Liberia
Solomon Islands
Rwanda
Cabo Verde
Egypt
Panama
Niue
Norway
Mauritania
Zimbabwe
El Salvador
Belize
Mexico
Trinidad and Tobago
Taiwan (Province of China)
Mali
Afghanistan
Guinea−Bissau
Malawi
Timor−Leste
Côte d'Ivoire
Haiti
Bangladesh
Marshall Islands
Venezuela (Bolivarian Republic of)
Jamaica
Northern Mariana Islands
Bahamas
Singapore
Burundi
Ethiopia
Sierra Leone
Eritrea
Kenya
Sri Lanka
Argentina
Israel
Burkina Faso
Bhutan
Nigeria
Congo
Fiji
Brazil
Mauritius
Malaysia
Brunei Darussalam
Guam
Mozambique
Namibia
Lebanon
Qatar
Central African Republic
Democratic Republic of the Congo
Senegal
United Republic of Tanzania
Djibouti
Cameroon
Tajikistan
Morocco
Palestine
Botswana
Samoa
Indonesia
Equatorial Guinea
Bahrain
Turkey
United Arab Emirates
Jordan
Kuwait
Algeria
South Sudan
Madagascar
Papua New Guinea
Yemen
Pakistan
Comoros
Angola
Sudan
Kyrgyzstan
Nauru
Syrian Arab Republic
Paraguay
Gabon
Tunisia
Iraq
Iran (Islamic Republic of)
Libya
American Samoa
Oman
Saudi Arabia

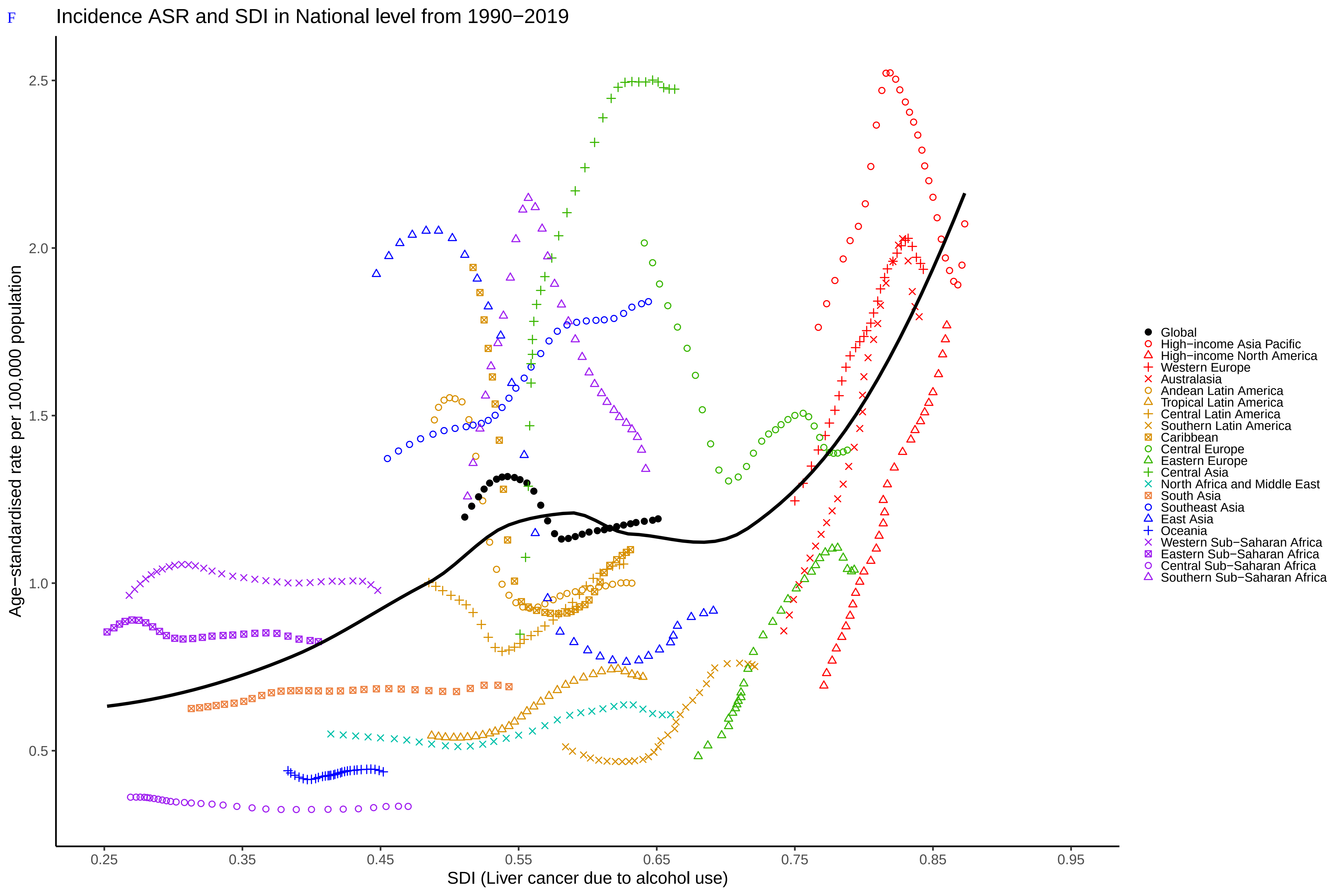
F
Incidence ASR and SDI in National level from 1990−2019
Age−standardised rate per 100,000 population
2.5
2.0
1.5
1.0
0.5
0.25
0.35
0.45
0.55
0.65
0.75
0.85
0.95
SDI (Liver cancer due to alcohol use)
Global
High−income Asia Pacific
High−income North America
Western Europe
Australasia
Andean Latin America
Tropical Latin America
Central Latin America
Southern Latin America
Caribbean
Central Europe
Eastern Europe
Central Asia
North Africa and Middle East
South Asia
Southeast Asia
East Asia
Oceania
Western Sub−Saharan Africa
Eastern Sub−Saharan Africa
Central Sub−Saharan Africa
Southern Sub−Saharan Africa

G

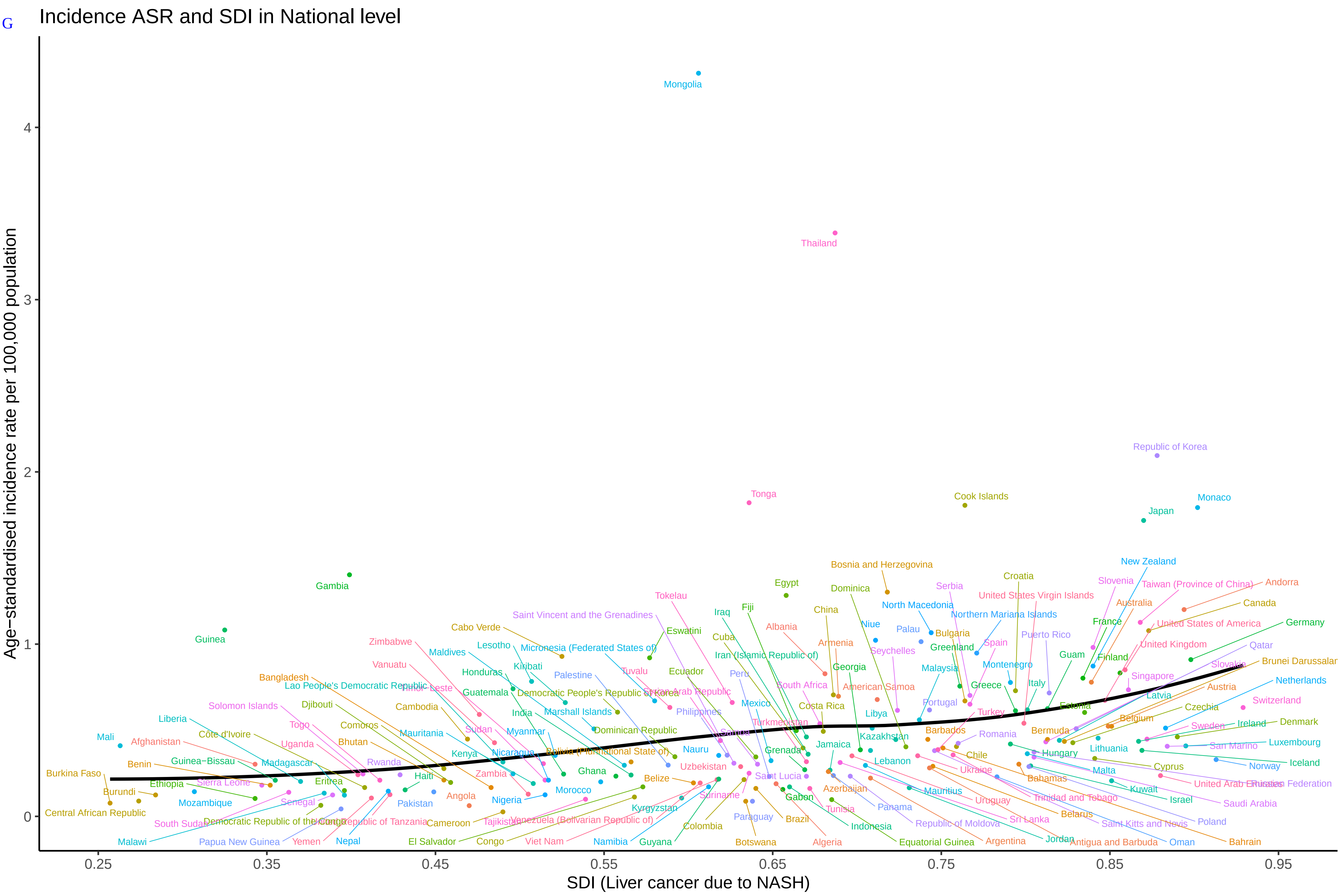

Incidence ASR and SDI in National level
Age-standardised incidence rate per 100,000 population
SDI (Liver cancer due to NASH)
0
1
2
3
4
0.25
0.35
0.45
0.55
0.65
0.75
0.85
0.95
Mongolia
Thailand
Tonga
Cook Islands
Republic of Korea
Monaco
Japan
Gambia
Guinea
New Zealand
Bosnia and Herzegovina
Croatia
Egypt
Dominica
Serbia
Slovenia
Taiwan (Province of China)
Andorra
Tokelau
United States Virgin Islands
Australia
Canada
Fiji
China
North Macedonia
Iraq
Northern Mariana Islands
France
United States of America
Germany
Saint Vincent and the Grenadines
Albania
Niue
Palau
Bulgaria
Puerto Rico
Cabo Verde
Eswatini
Cuba
Armenia
Seychelles
Greenland
Spain
Guam
United Kingdom
Qatar
Zimbabwe
Lesotho
Micronesia (Federated States of)
Iran (Islamic Republic of)
Finland
Maldives
Kiribati
Georgia
Malaysia
Montenegro
Slovakia
Brunei Darussalam
Vanuatu
Honduras
Tuvalu
Ecuador
Singapore
Bangladesh
Palestine
Peru
South Africa
Greece
Italy
Austria
Netherlands
Lao People's Democratic Republic
Timor-Leste
Syrian Arab Republic
American Samoa
Latvia
Guatemala
Democratic People's Republic of Korea
Mexico
Portugal
Estonia
Switzerland
Solomon Islands
Djibouti
Cambodia
Costa Rica
Czechia
India
Marshall Islands
Philippines
Libya
Turkey
Belgium
Liberia
Turkmenistan
Sweden
Ireland
Denmark
Togo
Sudan
Myanmar
Dominican Republic
Samoa
Barbados
Romania
Bermuda
Mali
Comoros
Mauritania
Kazakhstan
Côte d'Ivoire
Afghanistan
Bhutan
Uganda
Kenya
Nicaragua
Bolivia (Plurinational State of)
Nauru
Grenada
Jamaica
Chile
Hungary
Lithuania
Luxembourg
San Marino
Guinea-Bissau
Madagascar
Rwanda
Uzbekistan
Lebanon
Ukraine
Malta
Cyprus
Norway
Iceland
Benin
Zambia
Ghana
Saint Lucia
Burkina Faso
Haiti
Bahamas
Ethiopia
Sierra Leone
Eritrea
Belize
Azerbaijan
Kuwait
United Arab Emirates
Russian Federation
Burundi
Angola
Morocco
Suriname
Gabon
Mauritius
Trinidad and Tobago
Israel
Mozambique
Senegal
Pakistan
Nigeria
Uruguay
Saudi Arabia
Central African Republic
Kyrgyzstan
Paraguay
Brazil
Tunisia
Panama
Belarus
Sri Lanka
South Sudan
Democratic Republic of the Congo
United Republic of Tanzania
Cameroon
Tajikistan
Venezuela (Bolivarian Republic of)
Colombia
Indonesia
Republic of Moldova
Saint Kitts and Nevis
Poland
Malawi
Papua New Guinea
Yemen
Nepal
El Salvador
Congo
Viet Nam
Namibia
Guyana
Botswana
Algeria
Equatorial Guinea
Argentina
Jordan
Antigua and Barbuda
Oman
Bahrain

H

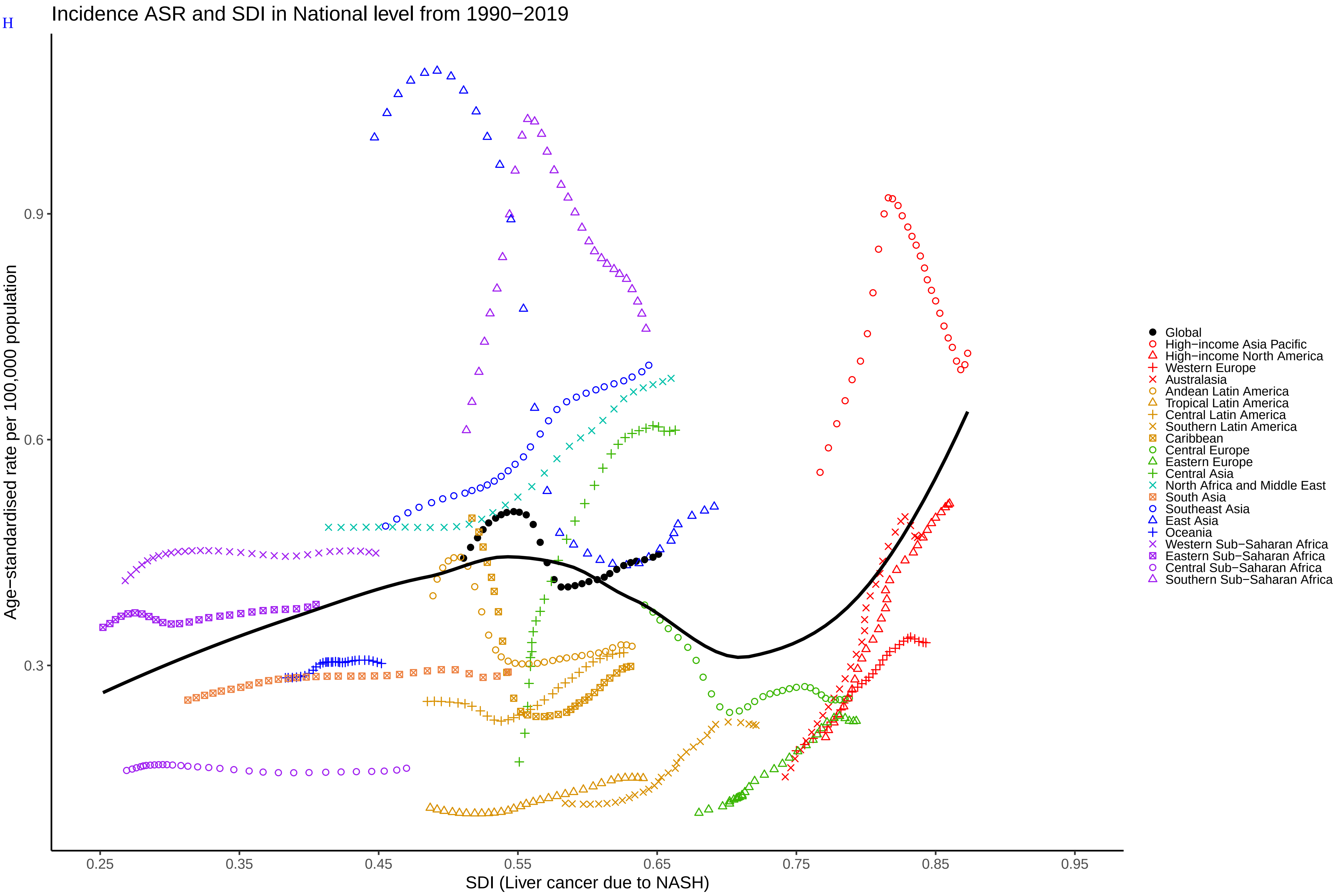

Incidence ASR and SDI in National level from 1990−2019
Age−standardised rate per 100,000 population
0.3
0.6
0.9
0.25
0.35
0.45
0.55
0.65
0.75
0.85
0.95
SDI (Liver cancer due to NASH)
Global
High−income Asia Pacific
High−income North America
Western Europe
Australasia
Andean Latin America
Tropical Latin America
Central Latin America
Southern Latin America
Caribbean
Central Europe
Eastern Europe
Central Asia
North Africa and Middle East
South Asia
Southeast Asia
East Asia
Oceania
Western Sub−Saharan Africa
Eastern Sub−Saharan Africa
Central Sub−Saharan Africa
Southern Sub−Saharan Africa

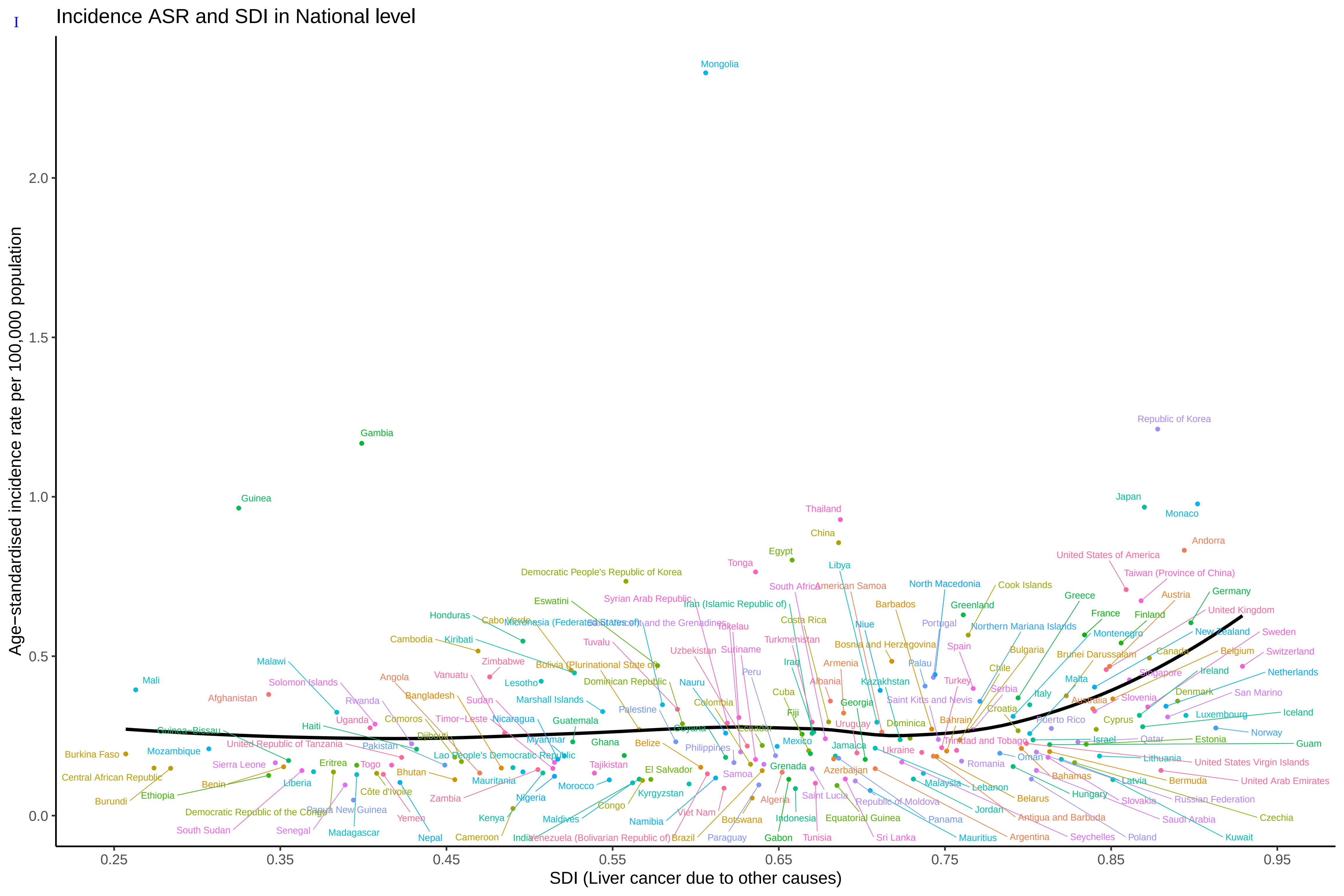
I
Incidence ASR and SDI in National level
Age−standardised incidence rate per 100,000 population
2.0
1.5
1.0
0.5
0.0
0.25
0.35
0.45
0.55
0.65
0.75
0.85
0.95
SDI (Liver cancer due to other causes)
Mongolia
Gambia
Guinea
Mali
Republic of Korea
Japan
Monaco
Thailand
China
Andorra
Egypt
United States of America
Tonga
Libya
Taiwan (Province of China)
Democratic People's Republic of Korea
South Africa
American Samoa
North Macedonia
Cook Islands
Germany
Austria
Eswatini
Syrian Arab Republic
Iran (Islamic Republic of)
Barbados
Greenland
Greece
United Kingdom
Honduras
Cabo Verde
Micronesia (Federated States of)
Saint Vincent and the Grenadines
Costa Rica
Niue
Portugal
Northern Mariana Islands
France
Finland
New Zealand
Sweden
Montenegro
Tokelau
Cambodia
Kiribati
Tuvalu
Turkmenistan
Bosnia and Herzegovina
Spain
Bulgaria
Brunei Darussalam
Canada
Belgium
Switzerland
Uzbekistan
Suriname
Malawi
Zimbabwe
Bolivia (Plurinational State of)
Iraq
Armenia
Palau
Chile
Ireland
Netherlands
Singapore
Vanuatu
Angola
Lesotho
Dominican Republic
Nauru
Peru
Albania
Kazakhstan
Turkey
Malta
Solomon Islands
Serbia
Denmark
San Marino
Afghanistan
Bangladesh
Marshall Islands
Cuba
Georgia
Saint Kitts and Nevis
Italy
Australia
Slovenia
Rwanda
Sudan
Colombia
Palestine
Fiji
Croatia
Luxembourg
Iceland
Uganda
Comoros
Timor−Leste
Nicaragua
Guatemala
Uruguay
Dominica
Bahrain
Puerto Rico
Cyprus
Haiti
Guyana
Ecuador
Norway
Guinea−Bissau
Djibouti
Myanmar
Ghana
Belize
Mexico
Qatar
Estonia
Israel
Guam
Burkina Faso
Mozambique
United Republic of Tanzania
Pakistan
Philippines
Jamaica
Ukraine
Trinidad and Tobago
Lao People's Democratic Republic
Oman
Lithuania
United States Virgin Islands
Sierra Leone
Eritrea
Togo
Tajikistan
Grenada
Romania
Central African Republic
Bhutan
Mauritania
El Salvador
Samoa
Azerbaijan
Bahamas
Latvia
Bermuda
United Arab Emirates
Benin
Liberia
Morocco
Malaysia
Lebanon
Ethiopia
Côte d'Ivoire
Zambia
Nigeria
Kyrgyzstan
Algeria
Saint Lucia
Belarus
Hungary
Slovakia
Russian Federation
Burundi
Democratic Republic of the Congo
Papua New Guinea
Congo
Viet Nam
Republic of Moldova
Jordan
Czechia
Yemen
Kenya
Maldives
Namibia
Botswana
Indonesia
Equatorial Guinea
Panama
Antigua and Barbuda
Saudi Arabia
South Sudan
Senegal
Madagascar
Nepal
Cameroon
India
Venezuela (Bolivarian Republic of)
Brazil
Paraguay
Gabon
Tunisia
Sri Lanka
Mauritius
Argentina
Seychelles
Poland
Kuwait

J

Incidence ASR and SDI in National level from 1990−2019

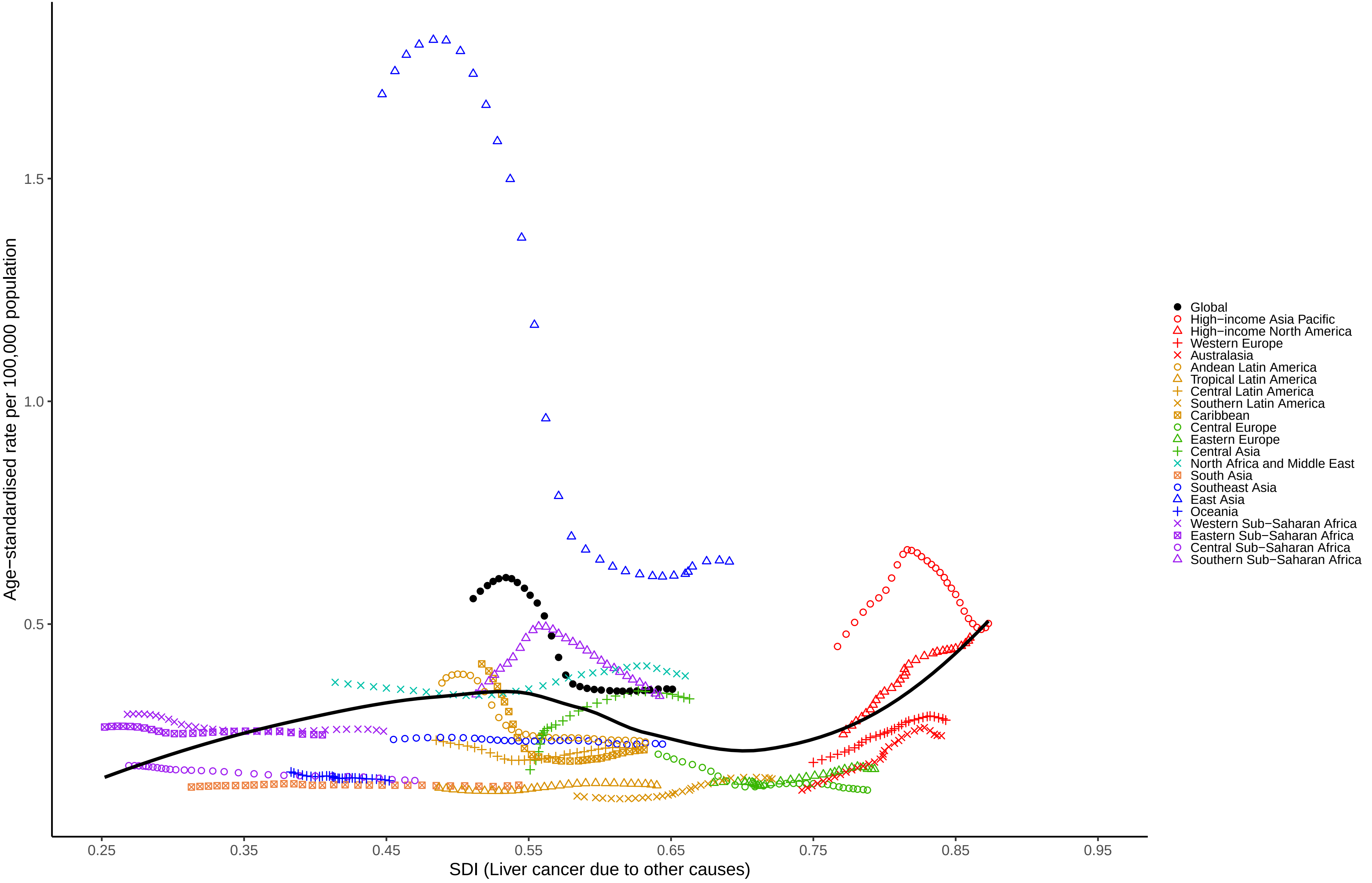

Supplement: Supplementary file 3 — Figure S3 [file CAM4-11-1310-s003.pdf]
