# Supplementary material for: Global burden of primary liver cancer by five etiologies and global prediction by 2035 based on global burden of disease study 2019
Source: Cancer Med. 2022 Feb 4;11(5):1310–23. doi: 10.1002/cam4.4551 (PMC8894698; doi:10.1002/cam4.4551)
Supplement: Supplementary file 4 — Figure S4 [file CAM4-11-1310-s005.pdf]

A

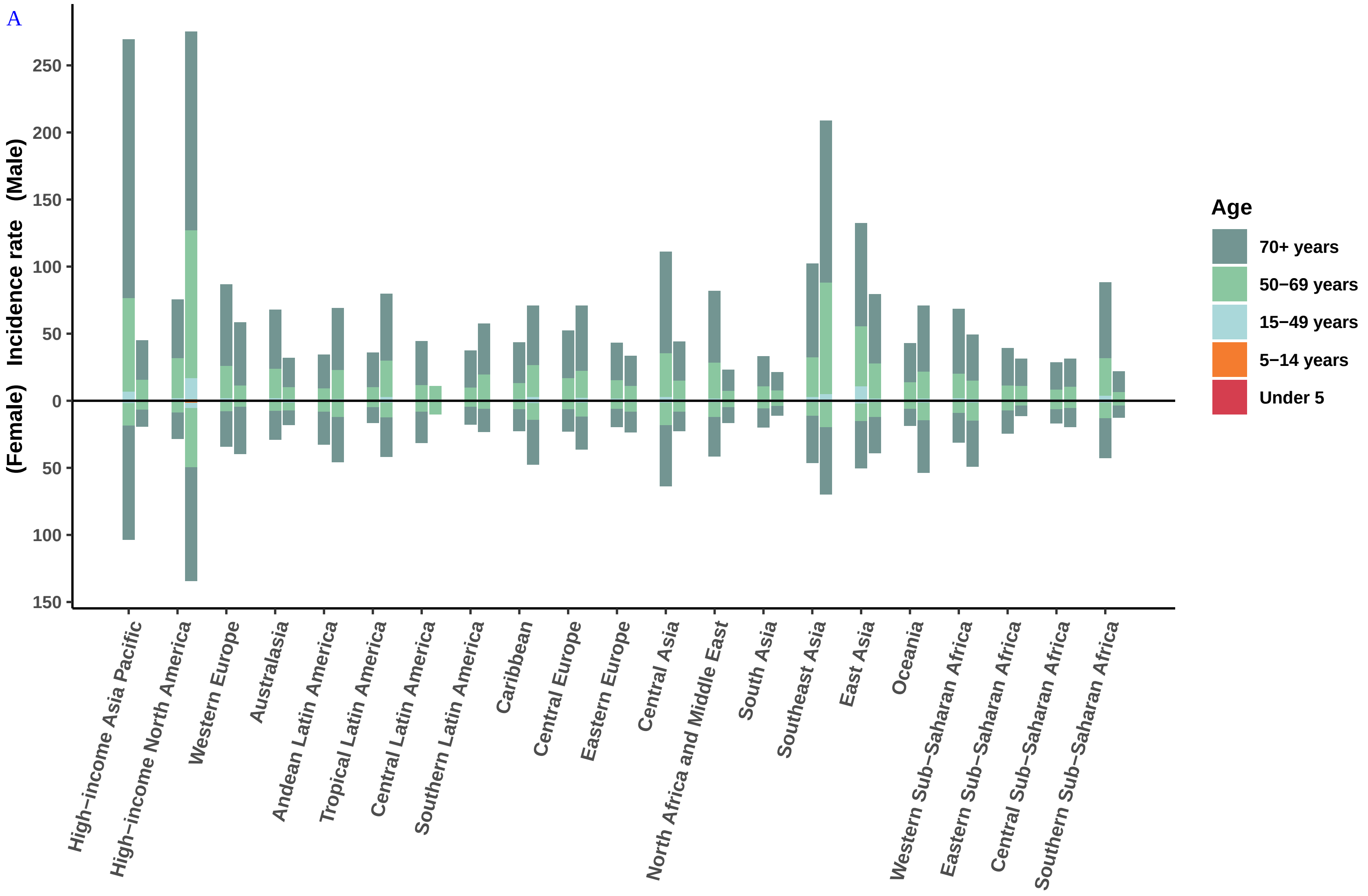

250
200
150
100
50
0
50
100
150
(Female) Incidence rate (Male)
Age
70+ years
50−69 years
15−49 years
5−14 years
Under 5
High−income Asia Pacific
High−income North America
Western Europe
Australasia
Andean Latin America
Tropical Latin America
Central Latin America
Southern Latin America
Caribbean
Central Europe
Eastern Europe
Central Asia
North Africa and Middle East
South Asia
Southeast Asia
East Asia
Oceania
Western Sub−Saharan Africa
Eastern Sub−Saharan Africa
Central Sub−Saharan Africa
Southern Sub−Saharan Africa

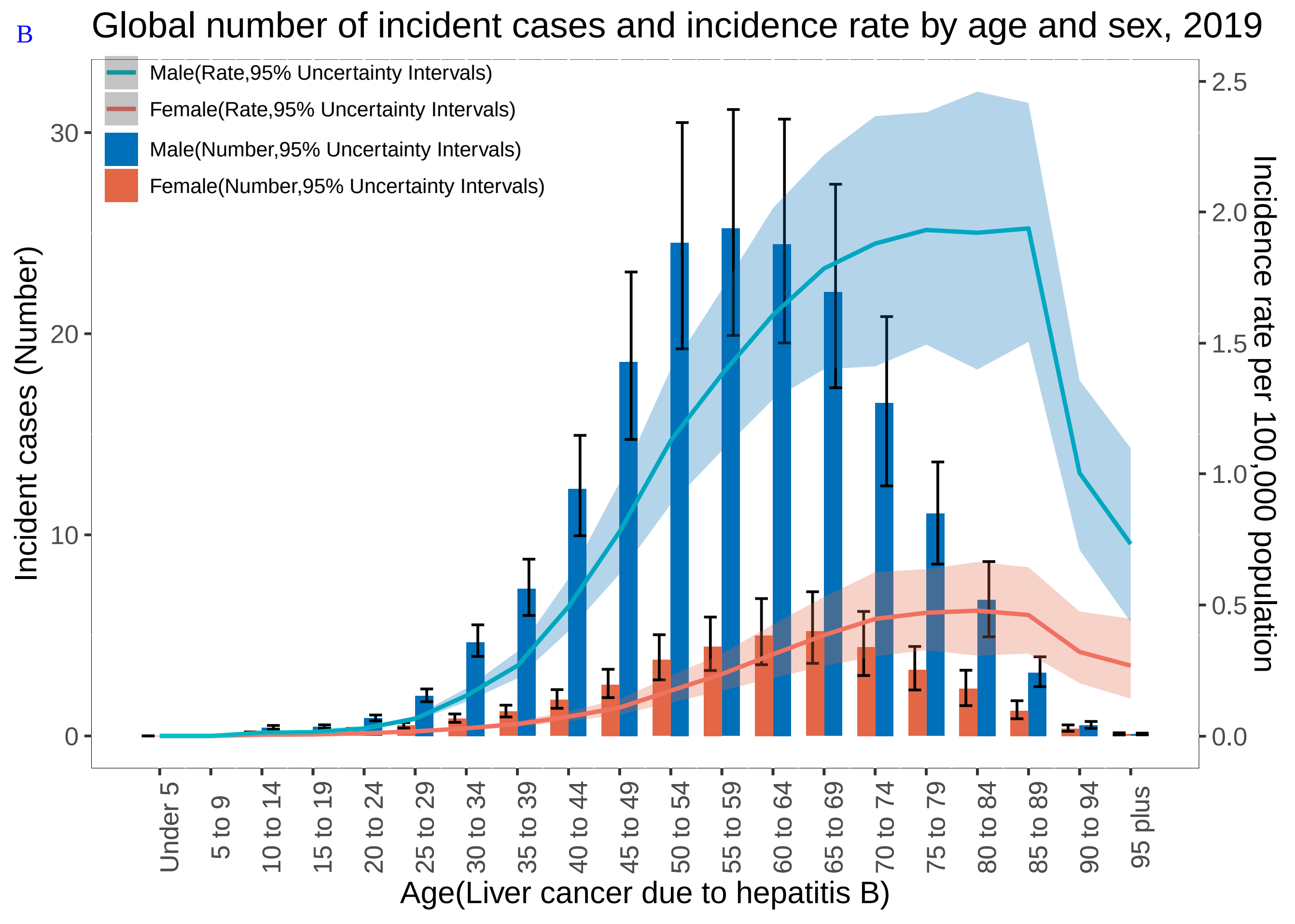
B
Global number of incident cases and incidence rate by age and sex, 2019
Male(Rate,95% Uncertainty Intervals)
Female(Rate,95% Uncertainty Intervals)
Male(Number,95% Uncertainty Intervals)
Female(Number,95% Uncertainty Intervals)
Incident cases (Number)
Incidence rate per 100,000 population
0
10
20
30
0.0
0.5
1.0
1.5
2.0
2.5
Under 5
5 to 9
10 to 14
15 to 19
20 to 24
25 to 29
30 to 34
35 to 39
40 to 44
45 to 49
50 to 54
55 to 59
60 to 64
65 to 69
70 to 74
75 to 79
80 to 84
85 to 89
90 to 94
95 plus
Age(Liver cancer due to hepatitis B)

C

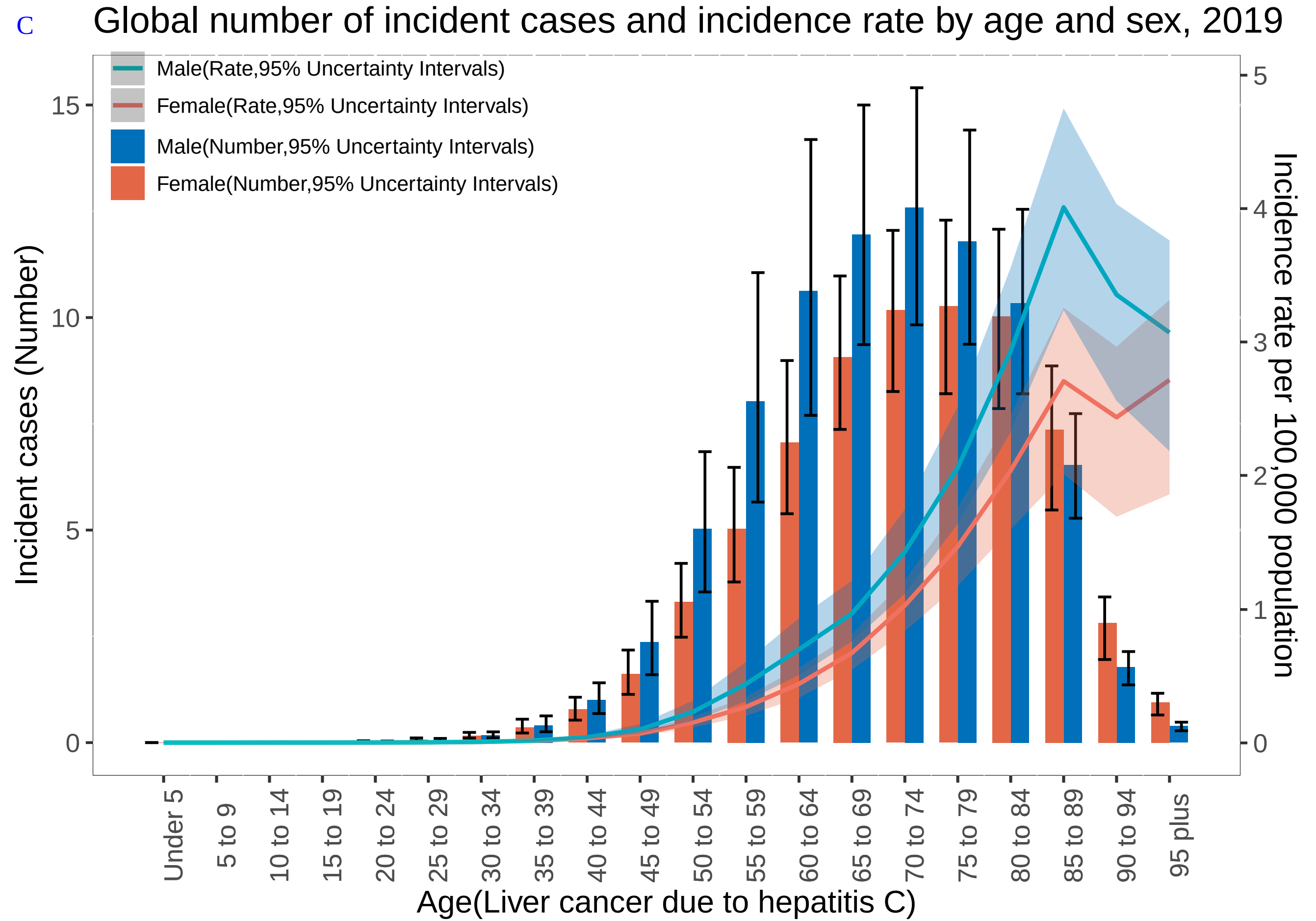
Global number of incident cases and incidence rate by age and sex, 2019
Male(Rate,95% Uncertainty Intervals)
Female(Rate,95% Uncertainty Intervals)
Male(Number,95% Uncertainty Intervals)
Female(Number,95% Uncertainty Intervals)
Incident cases (Number)
Incidence rate per 100,000 population
0
5
10
15
0
1
2
3
4
5
Under 5
5 to 9
10 to 14
15 to 19
20 to 24
25 to 29
30 to 34
35 to 39
40 to 44
45 to 49
50 to 54
55 to 59
60 to 64
65 to 69
70 to 74
75 to 79
80 to 84
85 to 89
90 to 94
95 plus
Age(Liver cancer due to hepatitis C)

D

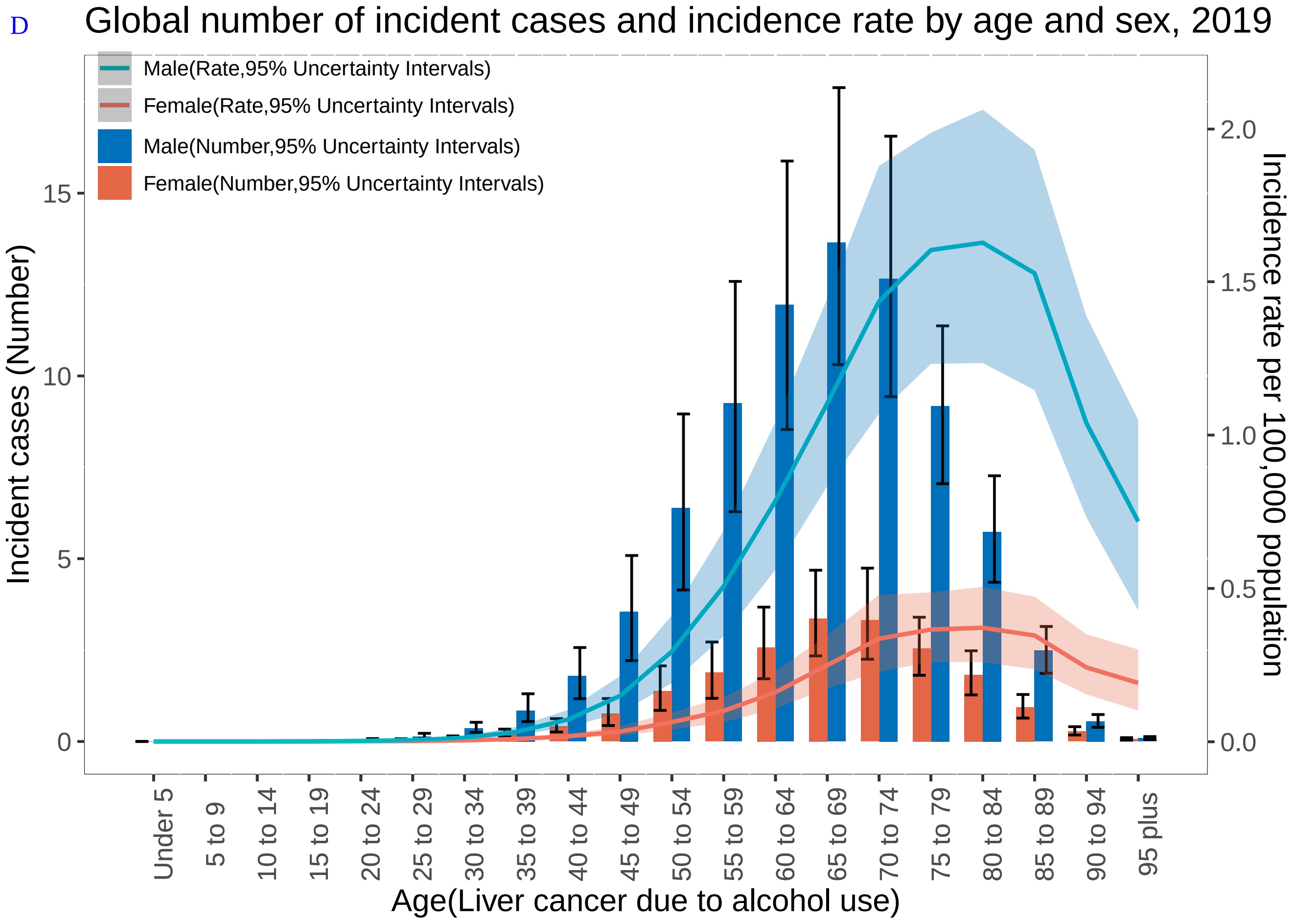

Global number of incident cases and incidence rate by age and sex, 2019
Male(Rate,95% Uncertainty Intervals)
Female(Rate,95% Uncertainty Intervals)
Male(Number,95% Uncertainty Intervals)
Female(Number,95% Uncertainty Intervals)
Incident cases (Number)
Incidence rate per 100,000 population
0
5
10
15
0.0
0.5
1.0
1.5
2.0
Under 5
5 to 9
10 to 14
15 to 19
20 to 24
25 to 29
30 to 34
35 to 39
40 to 44
45 to 49
50 to 54
55 to 59
60 to 64
65 to 69
70 to 74
75 to 79
80 to 84
85 to 89
90 to 94
95 plus
Age(Liver cancer due to alcohol use)

E

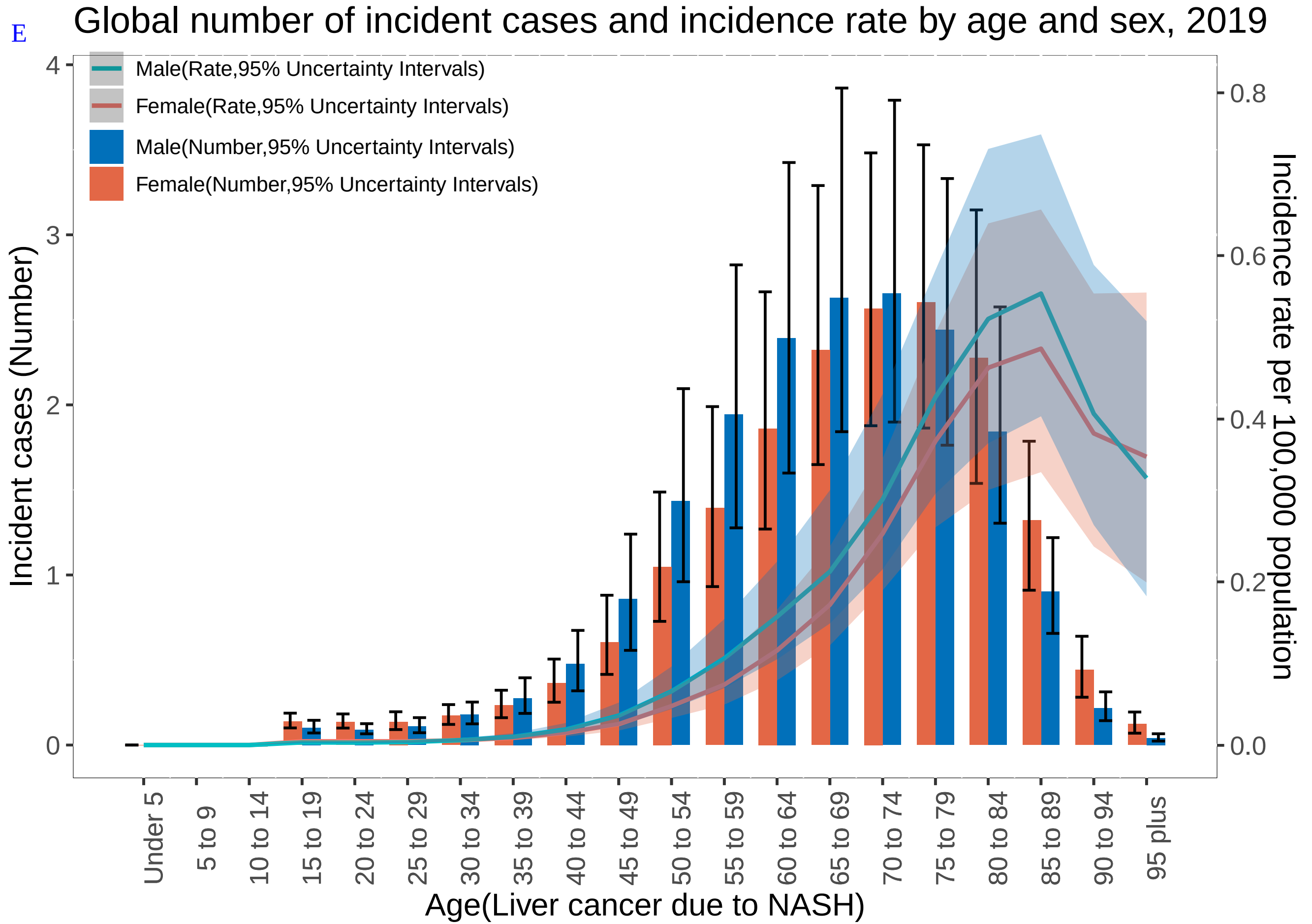
Global number of incident cases and incidence rate by age and sex, 2019
Male(Rate,95% Uncertainty Intervals)
Female(Rate,95% Uncertainty Intervals)
Male(Number,95% Uncertainty Intervals)
Female(Number,95% Uncertainty Intervals)
Incident cases (Number)
Incidence rate per 100,000 population
Age(Liver cancer due to NASH)
0
1
2
3
4
0.0
0.2
0.4
0.6
0.8
Under 5
5 to 9
10 to 14
15 to 19
20 to 24
25 to 29
30 to 34
35 to 39
40 to 44
45 to 49
50 to 54
55 to 59
60 to 64
65 to 69
70 to 74
75 to 79
80 to 84
85 to 89
90 to 94
95 plus

F

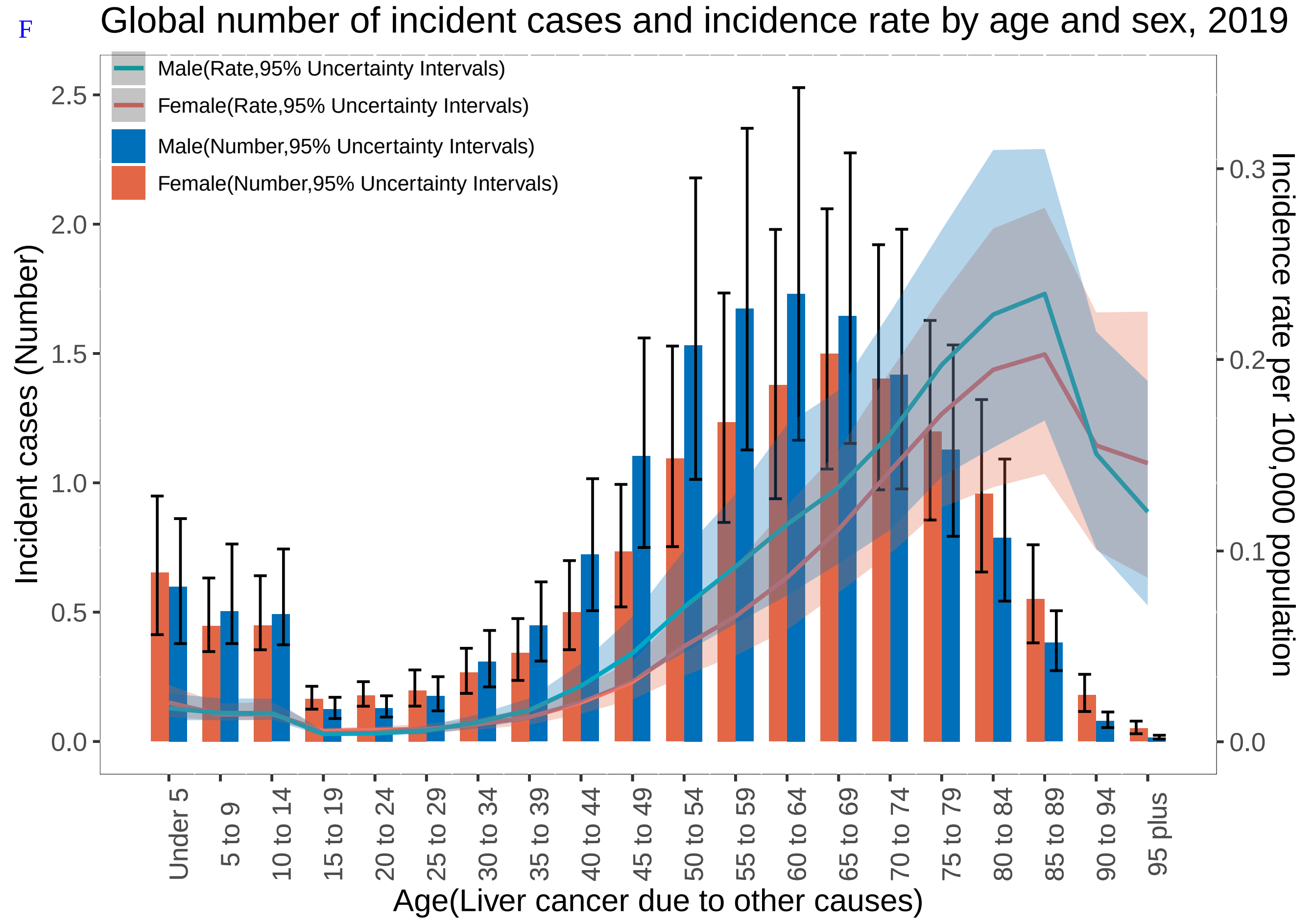

Global number of incident cases and incidence rate by age and sex, 2019
Male(Rate,95% Uncertainty Intervals)
Female(Rate,95% Uncertainty Intervals)
Male(Number,95% Uncertainty Intervals)
Female(Number,95% Uncertainty Intervals)
Incident cases (Number)
Incidence rate per 100,000 population
0.0
0.5
1.0
1.5
2.0
2.5
0.1
0.2
0.3
Under 5
5 to 9
10 to 14
15 to 19
20 to 24
25 to 29
30 to 34
35 to 39
40 to 44
45 to 49
50 to 54
55 to 59
60 to 64
65 to 69
70 to 74
75 to 79
80 to 84
85 to 89
90 to 94
95 plus
Age(Liver cancer due to other causes)

G

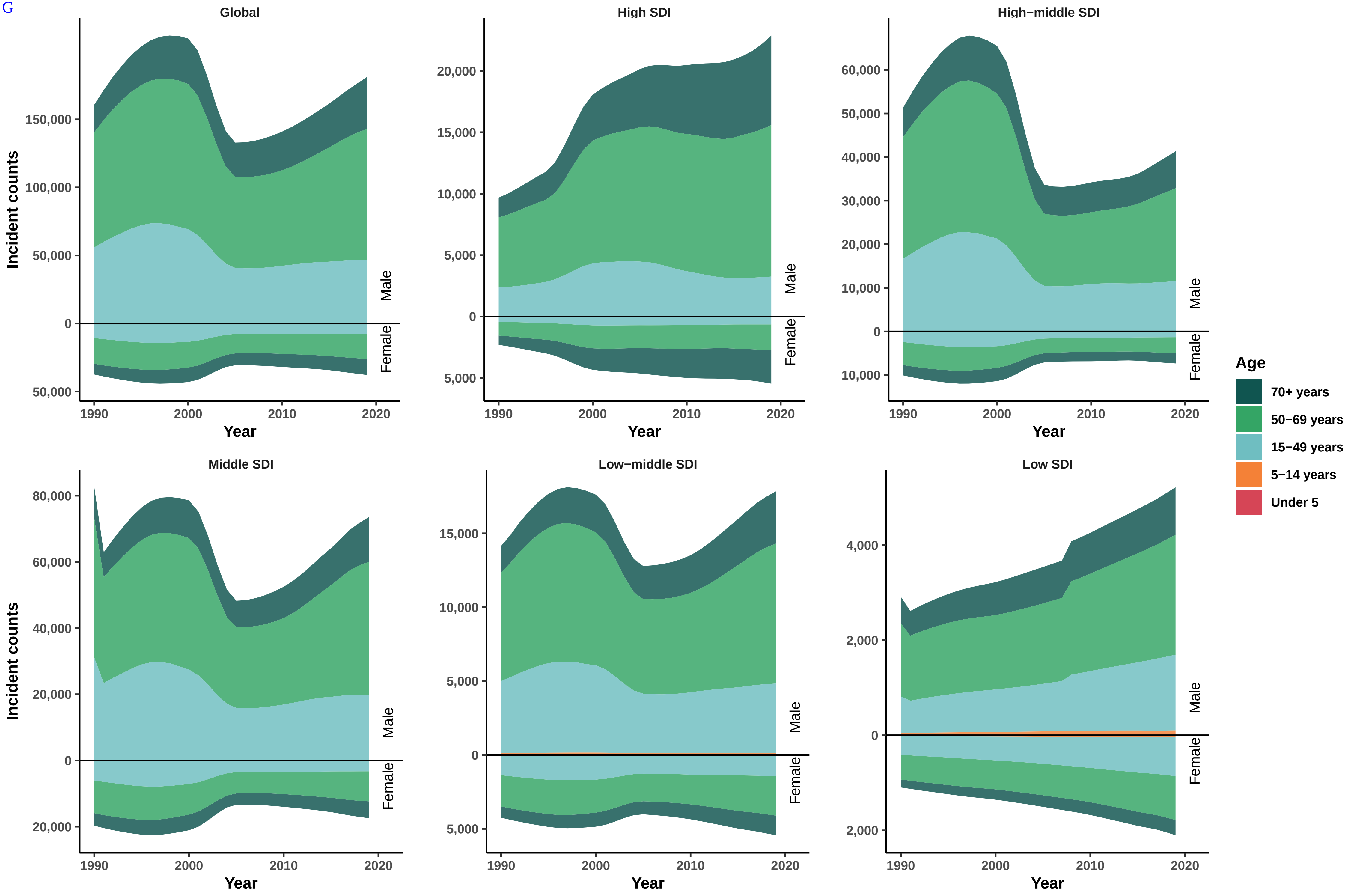
Global
High−SDI
High−middle SDI
Middle SDI
Low−middle SDI
Low SDI
Incident counts
Year
Male
Female
150,000
100,000
50,000
0
20,000
15,000
10,000
5,000
60,000
40,000
30,000
80,000
4,000
2,000
1990
2000
2010
2020
Age
70+ years
50−69 years
15−49 years
5−14 years
Under 5

H

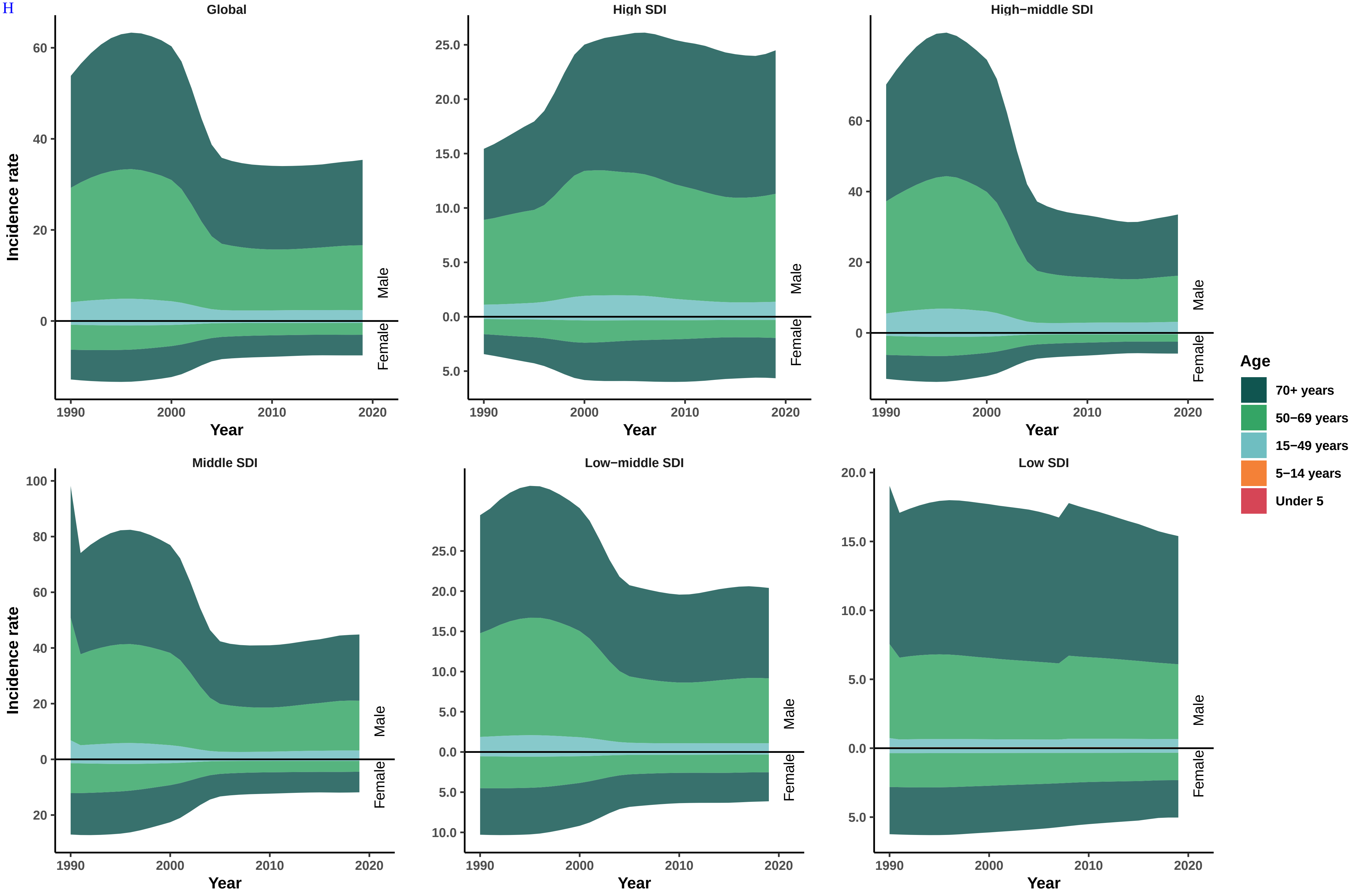
Global
High SDI
High−middle SDI
Middle SDI
Low−middle SDI
Low SDI
Incidence rate
Year
Male
Female
Age
70+ years
50−69 years
15−49 years
5−14 years
Under 5

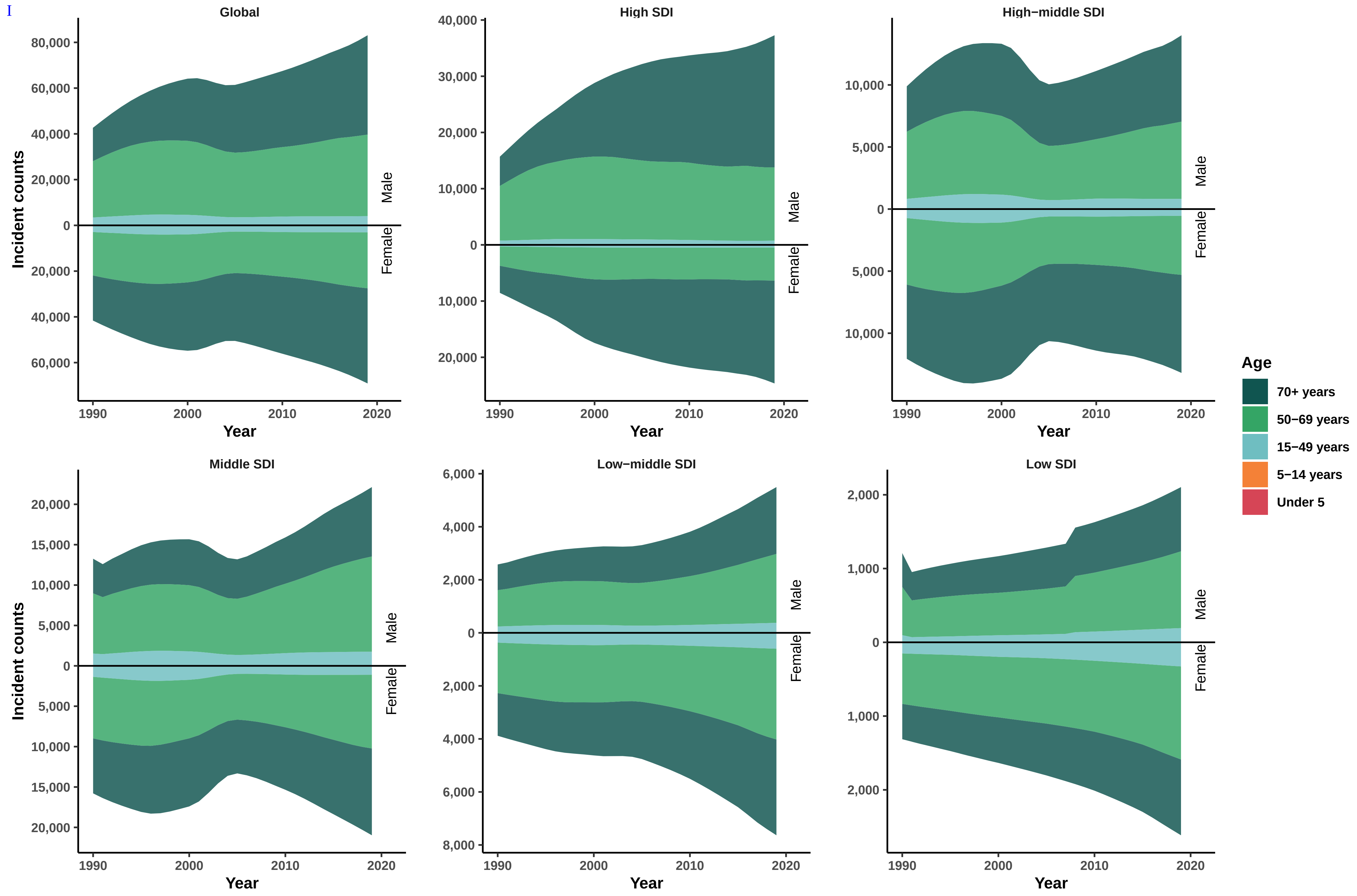
I
Global
High SDI
High−middle SDI
Middle SDI
Low−middle SDI
Low SDI
Incident counts
Year
Male
Female
Age
70+ years
50−69 years
15−49 years
5−14 years
Under 5

J

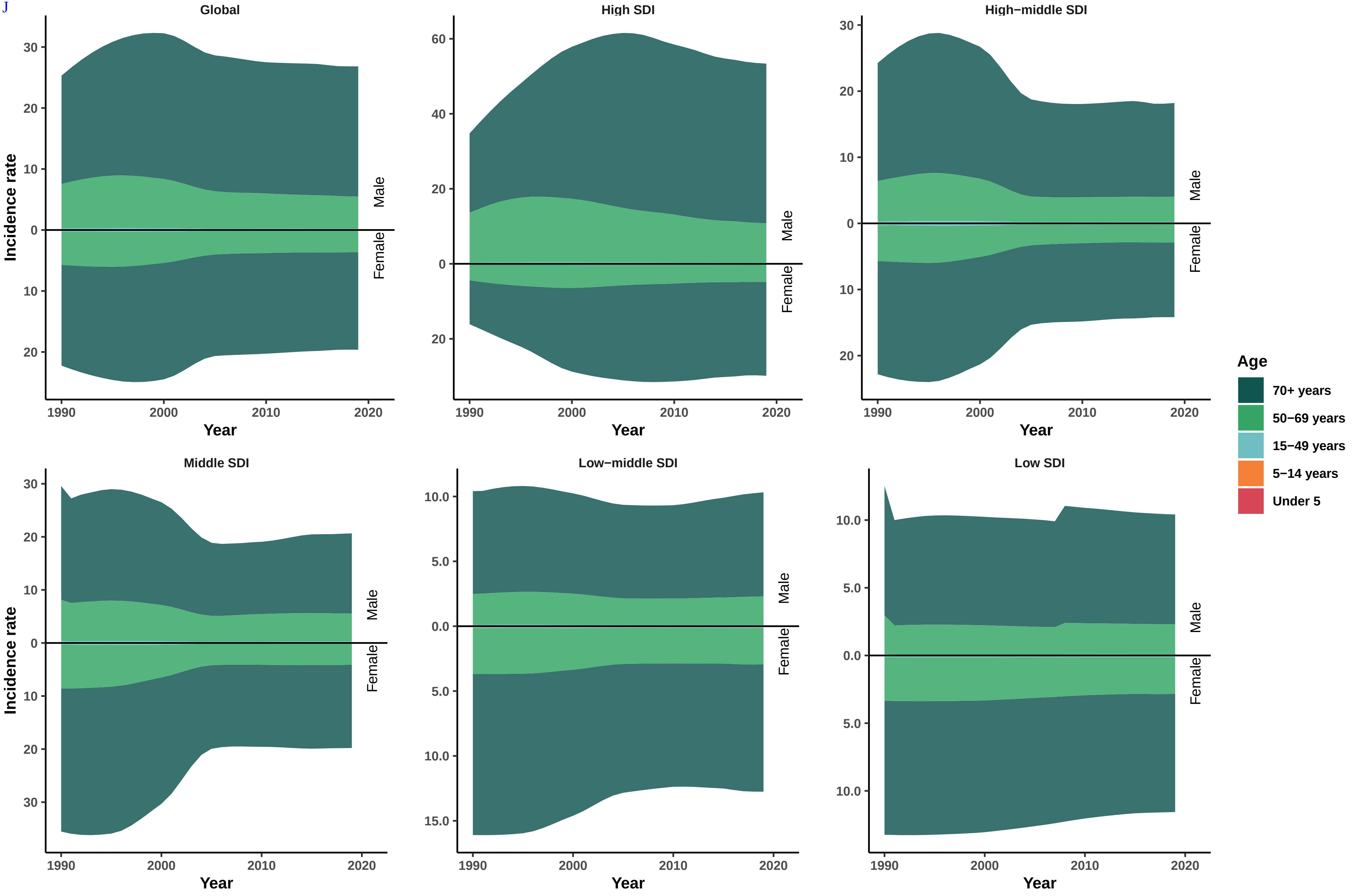

Global
High SDI
High−middle SDI
Middle SDI
Low−middle SDI
Low SDI
Incidence rate
Year
Male
Female
Age
70+ years
50−69 years
15−49 years
5−14 years
Under 5

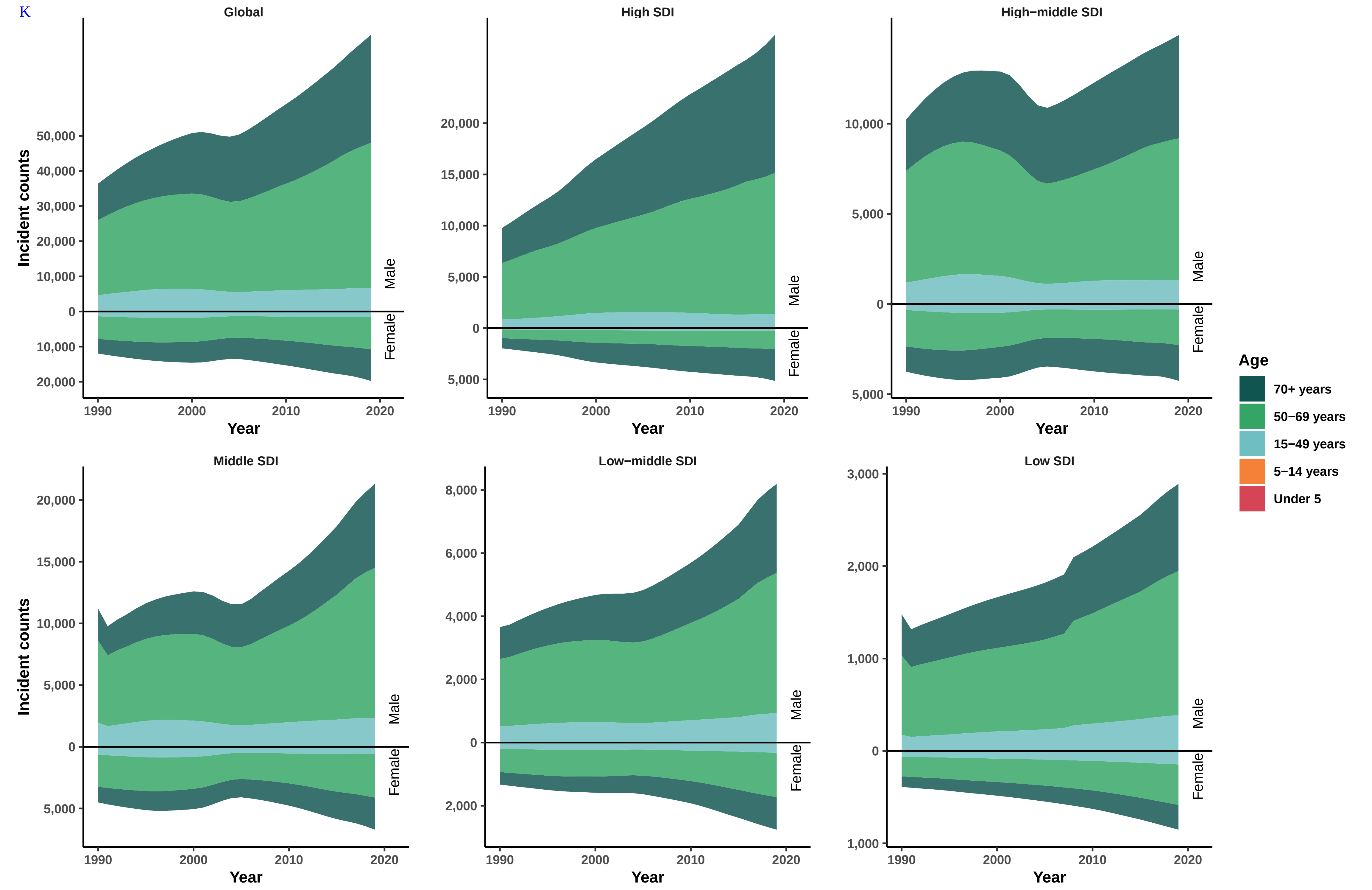
K
Global
High SDI
High−middle SDI
Middle SDI
Low−middle SDI
Low SDI
Incident counts
Year
Male
Female
Age
70+ years
50−69 years
15−49 years
5−14 years
Under 5

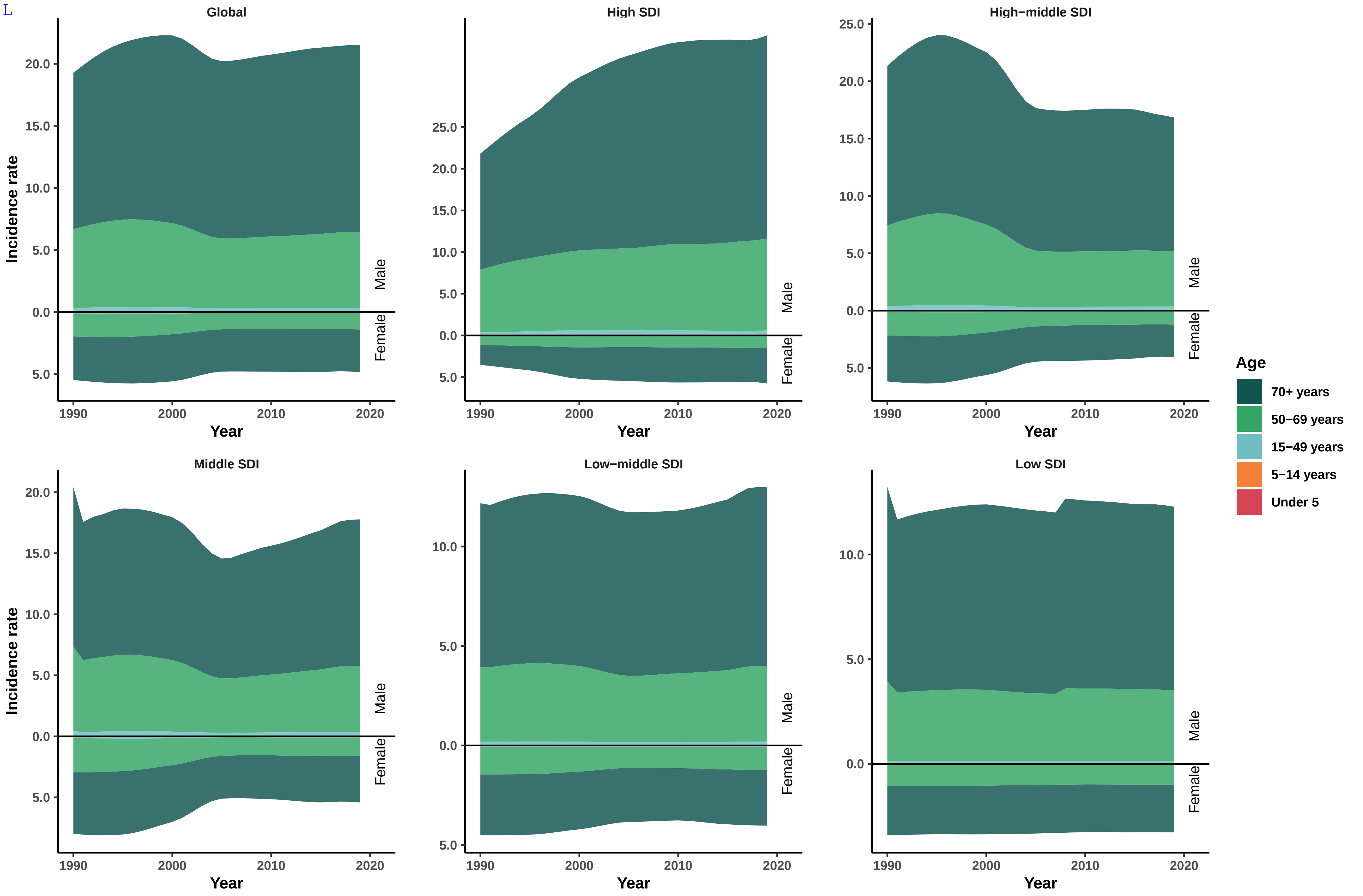
L
Global
High SDI
High−middle SDI
Middle SDI
Low−middle SDI
Low SDI
Incidence rate
Year
Male
Female
Age
70+ years
50−69 years
15−49 years
5−14 years
Under 5

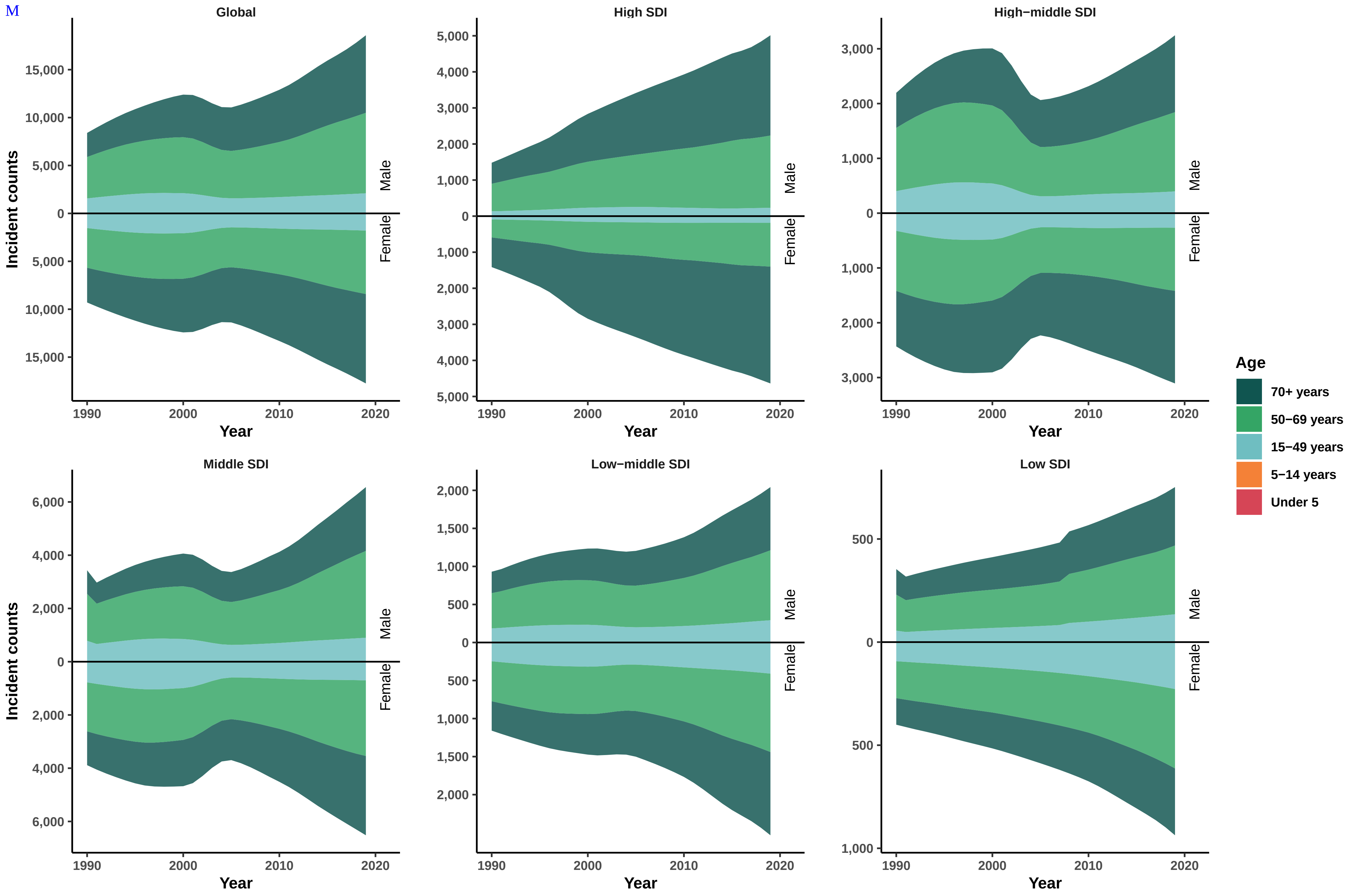
M
Global
High SDI
High−middle SDI
Middle SDI
Low−middle SDI
Low SDI
Incident counts
Year
Male
Female
Age
70+ years
50−69 years
15−49 years
5−14 years
Under 5

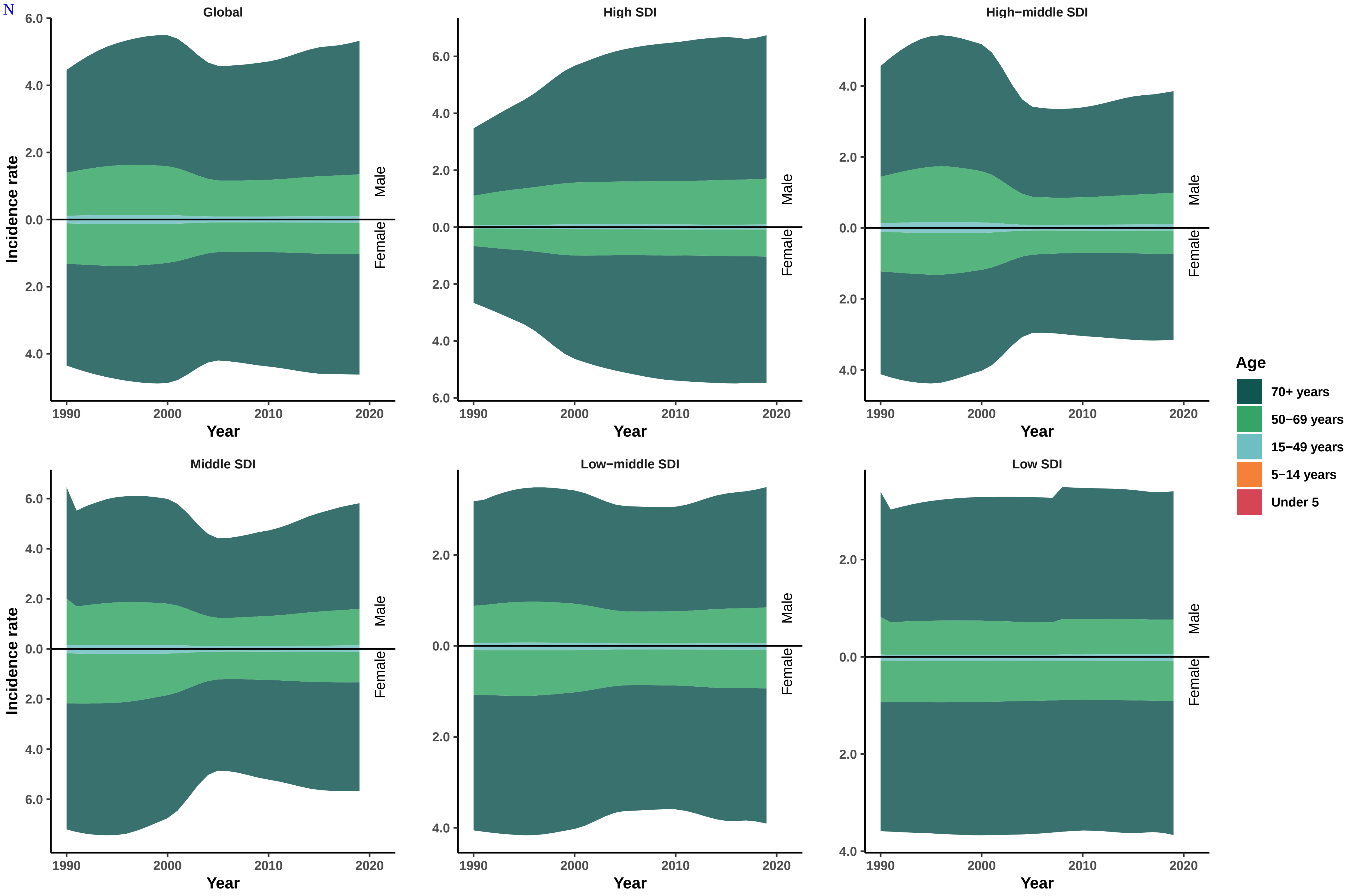

N
Global
High SDI
High−middle SDI
Middle SDI
Low−middle SDI
Low SDI
Incidence rate
Year
Male
Female
1990
2000
2010
2020
0.0
2.0
4.0
6.0
Age
70+ years
50−69 years
15−49 years
5−14 years
Under 5

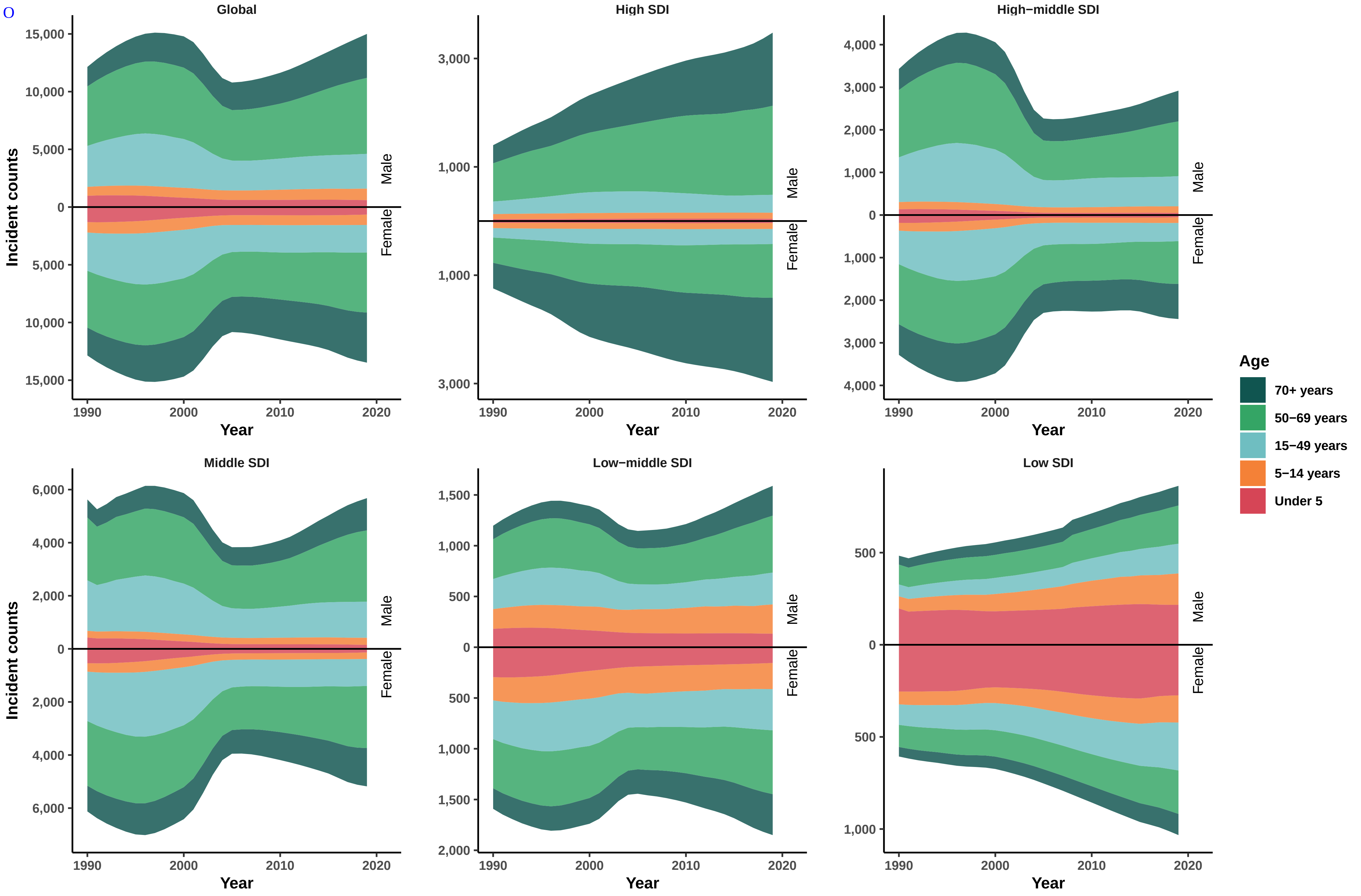
O
Global
High SDI
High−middle SDI
Middle SDI
Low−middle SDI
Low SDI
Incident counts
Year
Male
Female
Age
70+ years
50−69 years
15−49 years
5−14 years
Under 5

P

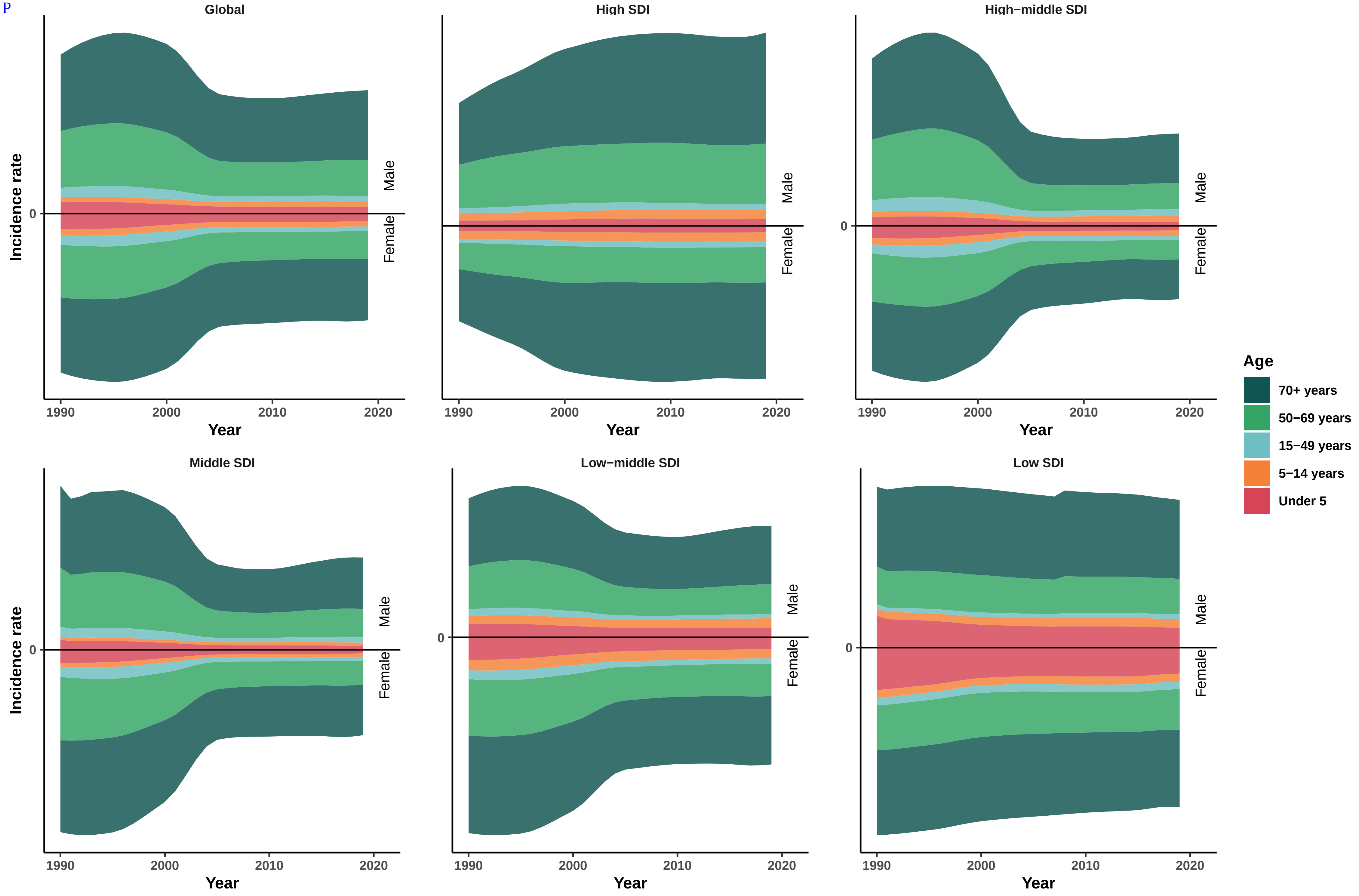
Global
High SDI
High−middle SDI
Middle SDI
Low−middle SDI
Low SDI
Incidence rate
0
Male
Female
1990
2000
2010
2020
Year
Age
70+ years
50−69 years
15−49 years
5−14 years
Under 5
